# Reconstructing the Evolutionary History of a Highly Conserved Operon Cluster in *Gammaproteobacteria* and *Bacilli*

**DOI:** 10.1093/gbe/evab041

**Published:** 2021-03-02

**Authors:** Gerrit Brandis

**Affiliations:** Department of Cell and Molecular Biology, Uppsala University, Biomedical Center, Sweden

**Keywords:** bacterial evolution, gene order, proteobacteria, firmicutes, SNAP, transposon

## Abstract

The evolution of gene order rearrangements within bacterial chromosomes is a fast process. Closely related species can have almost no conservation in long-range gene order. A prominent exception to this rule is a >40 kb long cluster of five core operons (*secE*-*rpoBC*-*str*-*S10*-*spc*-*alpha*) and three variable adjacent operons (*cysS*, *tufB*, and *ecf*) that together contain 57 genes of the transcriptional and translational machinery. Previous studies have indicated that at least part of this operon cluster might have been present in the last common ancestor of bacteria and archaea. Using 204 whole genome sequences, ∼2 Gy of evolution of the operon cluster were reconstructed back to the last common ancestors of the *Gammaproteobacteria* and of the *Bacilli*. A total of 163 independent evolutionary events were identified in which the operon cluster was altered. Further examination showed that the process of disconnecting two operons generally follows the same pattern. Initially, a small number of genes is inserted between the operons breaking the concatenation followed by a second event that fully disconnects the operons. While there is a general trend for loss of gene synteny over time, there are examples of increased alteration rates at specific branch points or within specific bacterial orders. This indicates the recurrence of relaxed selection on the gene order within bacterial chromosomes. The analysis of the alternation events indicates that segmental genome duplications and/or transposon-directed recombination play a crucial role in rearrangements of the operon cluster.

**Significance**

The linear order of genes within the bacterial chromosome is fluid, everchanging as life evolves, but little is known about the underlying mechanisms that drive chromosomal rearrangements on an evolutionary time scale. Here, the evolutionary history of a cluster of 57 genes was reconstructed for >2 billion years and 163 events were identified in which the linear order of the genes was altered. The analysis of these events indicates that gene duplications and transposon-directed recombination are potential forces that drive chromosome fluidity.

## Introduction

It is generally accepted that all life on earth has evolved from a universal common ancestor which would entail that all life forms share a single ancestral gene order (Woese 2000; Koonin 2003, 2014; Forterre 2015; Booth et al. 2016; Weiss et al. 2018). As life evolved, the order of genes on the chromosome changed over time until selection, genetic drift and horizontal gene transfer (HGT) removed almost all traces of the last common gene order from modern chromosomes (Koonin et al. 1996; Tatusov et al. 1996). Despite this general trend there are a few genes that display a significant degree of synteny across the bacterial domain of life which suggests that this gene order was present in at least the last common ancestor of all bacteria. This higher degree of synteny has been used to identify functional groups of genes, to supplement traditional phylogenetic analysis methods, and to reconstruct the organization of ancestral genomes (Overbeek et al. 1999; Snel et al. 2002; Wang et al. 2012; Anselmetti et al. 2015; Rajaraman and Ma 2016). These genes are generally organized within operons and gene synteny could be driven by selection for coregulation and/or the ability of horizontal transfer of fully functional units (Moreno-Hagelsieb et al. 2001; Price et al. 2005; Rocha 2006). Conservation on a higher order, operon synteny, is virtually absent in bacteria (Tamames 2001).

A prominent exception to this rule is the *secE*-*rpoBC*-*str*-*S10*-*spc*-*alpha* operon cluster (Watanabe et al. 1997; Wachtershauser 1998; Barloy-Hubler et al. 2001; Coenye and Vandamme 2005; Brandis et al. 2019). In the *Proteobacteria*, this cluster is ∼33 kb long and contains seven operons (*tufB*, *secE*, *rpoBC*, *str*, *S10*, *spc*, and *alpha*) that encode up to 50 genes of the transcriptional and translational machinery (precise numbers vary between species) and operon concatenation is maintained due to operon coregulation (Brandis et al. 2019). At least part of this operon cluster is similarly organized in archaea indicating that it might have been present in the last common ancestor of bacteria and archaea (Coenye and Vandamme 2005). The potential ancestral operon cluster included the genes encoding the main subunits of the RNA polymerase (RpoA, RpoB, and RpoC) (Zhang et al. 1999), up to 31 out of the 33 universal ribosomal proteins (Ban et al. 2014), three of the five translation initiation and elongation factors (IF-1, EF-Tu, and EF-G) (Ramakrishnan 2002), and two subunits of the Sec translocase (du Plessis et al. 2011). This indicates that the operon cluster could be a remnant of a primordial operon cluster encoding the full transcriptional and translational machinery. The synteny between genes in the operon cluster is among the most highly conserved in bacteria but even this cluster can be altered (Watanabe et al. 1997; Itoh et al. 1999; Tamames 2001; Coenye and Vandamme 2005). The exceptional degree of operon synteny conservation makes the *secE*-*rpoBC*-*str*-*S10*-*spc*-*alpha* operon cluster an ideal tool to study chromosomal reorganization on an evolutionary time scale.

Here, the organization of the *secE*-*rpoBC*-*str*-*S10*-*spc*-*alpha* operon cluster was compared in 204 modern species belonging to the *Proteobacteria*, *Acidobacteria*, *Firmicutes*, and *Tenericutes*. Using gene synteny and maximum parsimony, the ancestral operon cluster was reconstructed for the last common ancestor of the *Gammaproteobacteria* and the last common ancestor of the *Bacilli*. The evolutionary history from the ancestral *secE*-*rpoBC*-*str*-*S10*-*spc*-*alpha* operon clusters to the organization within the modern species was reconstructed. It was previously estimated that this corresponds to 2.5 Gy of evolution for the families of *Gammaproteobacteria* and 2.0 Gy of evolution for the families of *Bacilli* included in this study (Marin et al. 2017). A total of 163 independent evolutionary events (115 rearrangements and 48 deletions) were identified in which the operon cluster was altered. Ten events were further analyzed and molecular mechanisms that could be responsible for the operon alterations are discussed.

## Materials and Methods

### Software

Sequence alignments and tree constructions were performed using CLC Main Workbench version 8.1 (QIAGEN, Aarhus) and PhyML version 3.3.20190321 (Guindon et al. 2009).

### Phylogenetic Analysis

**Fig. 1.**
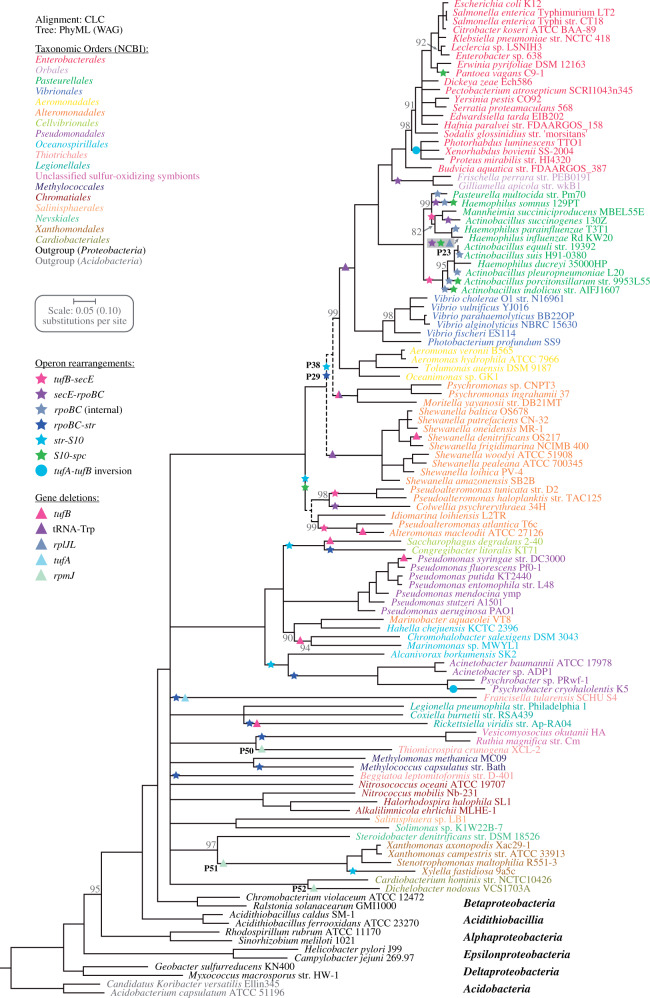
Phylogeny of the *Gammaproteobacteria*. A maximum likelihood phylogeny tree was produced using the PhyML algorithm (WAG substitution model) based on the concatenated CLC alignments of 39 proteins within the *secE*-*rpoBC*-*str*-*S10*-*spc*-*alpha* operon cluster (supplementary table S2, Supplementary Material online). Support for each node was evaluated by bootstrapping and nodes with a bootstrap value below 80% were collapsed. Support values for nodes are shown when these are below 100%. Two additional nodes that were identified based on the gene synteny analysis are indicated with dashed lines. Branch lengths for the outgroup species was reduced by a factor of two (see scale value in parenthesis). Taxonomic orders are designated according to NCBI and evolutionary events are indicated in the tree. See supplementary figure S9, Supplementary Material online and supplementary table S6, Supplementary Material online for event details.

**Fig. 2.**
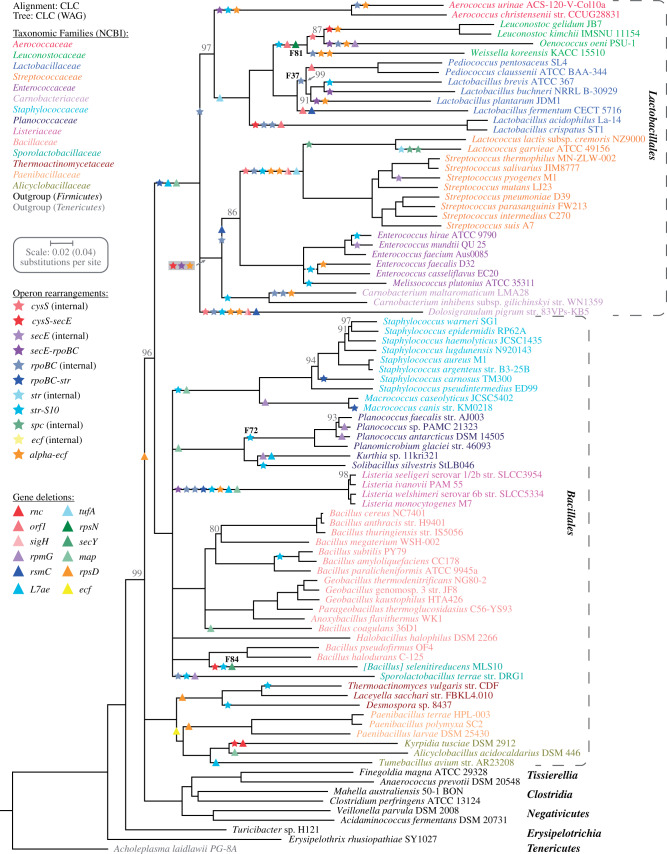
Phylogeny of the *Bacilli*. A maximum likelihood phylogeny tree was produced using the CLC algorithm (WAG substitution model) based on the concatenated CLC alignments of 44 proteins within the *secE*-*rpoBC*-*str*-*S10*-*spc*-*alpha* operon cluster (supplementary table S3, Supplementary Material online). Support for each node was evaluated by bootstrapping and nodes with a bootstrap value below 80% were collapsed. Support values for nodes are shown when these are below 100%. Branch lengths for the outgroup species was reduced by a factor of two (see scale value in parenthesis). Taxonomic orders and families are designated according to NCBI and evolutionary events are indicated in the tree. See supplementary figure S11 and supplementary table S6, Supplementary Material online for event details.

A total of 204 annotated genomes were downloaded from the NCBI database for the analysis (supplementary table S1, Supplementary Material online). Genes that were not annotated at their expected genetic locations within the operon cluster were manually checked to rule out annotation errors which were common for tRNAs and the small *rpmJ* gene. The genomes were split into two groups: 1) *Proteobacteria* and *Acidobacteria* (115 species) to reconstruct the evolution to the last common ancestor of the *Gammaproteobacteria*, and 2) *Firmicutes* and *Tenericutes* (89 species) for the reconstruction to the last common ancestor of the *Bacilli*. Protein alignments were performed with CLC using the CLC progressive alignment algorithm using the standard settings (Gap open cost: 10.0; Gap extension cost: 1.0; End gap cost: As any other; Alignment: Very accurate) and the MUSCLE alignment algorithm (Edgar 2004). No masking or local realignments were performed and all relevant protein alignments were concatenated. The *tufB* gene (duplicate of *tufA*) within the *Gammaproteobacteria* and *Acidobacteria* and genes that were absent in at least one modern genome were excluded from the analyses. For the *Proteobacteria* and *Acidobacteria* the concatenated alignment included 39 proteins with a total length of 9,429 amino acids (based on the mean length of each protein) and the concatenated alignment for the *Firmicutes* and *Tenericutes* included 44 proteins corresponding to 10,199 amino acids (supplementary tables S2 and S3, Supplementary Material online). Maximum likelihood phylogenetic trees based on the concatenated protein alignments were constructed using the CLC maximum likelihood phylogeny algorithm with standard settings (Tree construction method: maximum likelihood [Felsenstein 1981]; Protein substitution model: WAG [Whelan and Goldman 2001]; estimate topology [Felsenstein 1981]; Bootstrapping: 100 replicates [Efron 1982]) and the PhyML software with standard settings (Model of amino-acids substitution: LG [Le and Gascuel 2008] or WAG [Whelan and Goldman 2001]; Amino acid frequencies: model; Proportion of invariable sites: fixed [*P*-invar = 0.00]; One category of substitution rate: no; Number of substitution rate categories: 4; Gamma distribution rates across sites: yes; Gamma distribution parameter: estimated; Optimize tree topology: yes; Starting tree: BioNJ; Tree topology search operations: SPR moves; Add random starting trees: no; Nonparametric bootstrap analysis: yes [100 replicates]; Approximate likelihood ratio test: no). Bootstrapping was performed to provide support values and nodes with a bootstrap value below 80% were collapsed. Each tree was rooted between the two phyla that were part of the respective analysis and trees that did not properly separate the outgroups were removed from the further analysis. In total, three trees were produced to reconstruct the evolution to the last common ancestor of the *Gammaproteobacteria* and two for the reconstruction to the last common ancestor of the *Bacilli* (figs. 1 and 2, supplementary figs. S8–S12, Supplementary Material online).

### Reconstruction of Ancestral Operon Cluster Organization

Reconstruction of the ancestral operon cluster was performed using gene synteny and maximum parsimony. Each gene pair could take one of three states: 1) connected, 2) disconnected, and 3) one of the two genes is deleted. No distinction was made for the distance between two disconnected gene pairs (supplementary fig. S13, Supplementary Material online). For each potentially connected gene pair the number of minimal state changes was determined for the case that the gene pair is connected in the ancestral operon cluster (*N*_connected_) and for the case that the gene pair is not connected within the ancestral operon cluster (*N*_disconnected_). A gene pair was defined to be likely connected within the ancestral operon cluster if 1) *N*_connected_ ≤ *N*_disconnected_ and 2) the organization of the gene pair within the respective outgroup species agrees with the connection. The decision trees for all gene pairs that are not fully conserved throughout the *Gammaproteobacteria* or *Bacilli* are shown in supplementary figures S14 and S15, Supplementary Material online.

### Operon Assignments

**Fig. 3.**
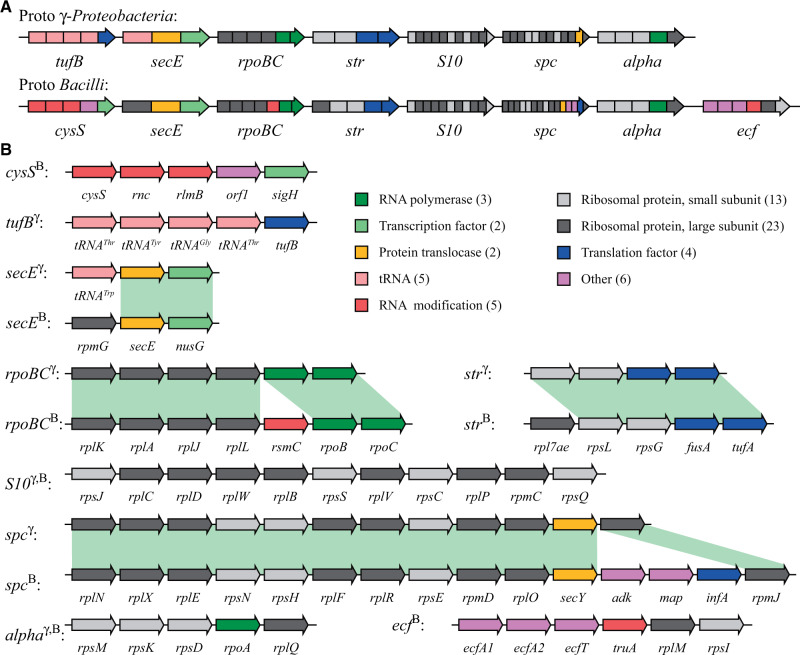
Reconstructed ancestral *secE*-*rpoBC*-*str*-*S10*-*spc*-*alpha* operon cluster in the last common ancestor of the *Gammaproteobacteria* and the last common ancestor of the *Bacilli*. (*A*) Overview over the operon concatenation. (*B*) Overview over the operon content. Operons present in the last common ancestor of the *Gammaproteobacteria* are indicated by a “γ” and operons present in the last common ancestor of the *Bacilli* are indicated by a “B.”

Operons in the ancestral operon clusters were defined according to the *E. coli* nomenclature. For three genes (*rpmG*, *tRNA-Trp* and *rpl7ae*) this assignment was ambiguous because they are not part of the operon cluster in *E. coli* and are located between two operons. The *rpmG* and *tRNA-Trp* genes were assigned to the *secE* operon and *rpl7ae* to the *str* operon according to the operon structure of the *Bacilli*. The ancestral operon cluster in the *Bacilli* contained five additional genes upstream (*cysS*-*rnc*-*rlmB*-*orf1*-*sigH*) and six additional genes downstream (*ecfA1*-*ecfA2*-*ecfT*-*truA*-*rplM*-*rpsI*) (fig. 3, supplementary table S5, Supplementary Material online). The additional genes identified upstream and downstream of the operon cluster in the *Bacilli* were grouped into two operons and named after their respective first gene: *cysS* and *ecf* (fig. 3).

### Identification of Evolutionary Events

Evolutionary events that alter the organization of the operon cluster (deletions or rearrangements) were identified by comparing the organization within modern species to the reconstructed ancestral organization. Initially, a gene synteny analysis was performed for all genomic regions that deviated from the proposed ancestral operon cluster organization. For regions with insertions (<10 kb sequence between two genes of the operon cluster), all inserted genes were included in the analysis. For gene pairs that were fully disconnected within a modern species, only the five first genes adjacent to the two respective disconnected genes were included (supplementary fig. S16*A* and supplementary tables S4, S5, Supplementary Material online). Novel gene neighborhoods were initially identified based on the annotated protein function and comparison of sequence lengths. Uncertain gene neighborhoods (based on a single proteins or common protein functions) were further confirmed by protein sequence alignments using the CLC alignment algorithm as described above. Evolutionary events were then identified with all five constructed trees (supplementary figs. S8–S12, Supplementary Material online) using maximum parsimony and the results of the gene synteny analysis. Novel gene neighborhoods that were identified were set to be dominant so that not all descendent species were required to contain the specific rearrangement (e.g., due to loss of the inserted genes). A layout for the identification process is shown in supplementary figure S16*B*, Supplementary Material online. The trees that described the evolutionary events with the least alterations and highest bootstrap values were chosen for further analysis (figs. 1 and 2).

### Test for Long Distance HGT

Each of the deletions and rearrangements that were part of the in-depth analysis were tested for the contribution of HGT from distantly related species. Long distance HGT was tested by comparison of the gene trees of the potentially transferred genes with their associated species trees (figs. 1 and 2). For deletion events, potentially transferred genes were defined as the corresponding copy of the gene located outside the operon cluster. For the rearrangement events, potentially transferred genes were defined as the genes included in the minimal inserted segment (fig. 6). Species were included in the analysis based on two parameters: 1) species must include the full set of genes to be analyzed, and 2) the chosen set must include closely related species before and after the potential HGT event. Maximum likelihood phylogenetic trees based on the concatenated alignments of the proteins to be tested were constructed using the identical settings that were used for constructing the respective species trees. The generated gene trees were then compared with their associated species trees and the RF distance was calculated based on an 80% bootstrap threshold (Robinson and Foulds 1981).

## Results and Discussion

### Reconstruction of the Ancestral Operon Clusters

A total of 204 bacterial genomes within the *Proteobacteria*, *Acidobacteria*, *Firmicutes*, and *Tenericutes* were chosen for the analysis of the operon cluster (supplementary table S1, Supplementary Material online). The majority of genomes chosen belong to the *Gammaproteobacteria* (103 genomes) and the *Bacilli* (80 genomes) to support a detailed analysis within these two important classes of bacteria and to include both Gram-negative and Gram-positive bacteria in the study. Based on a phylogenetic analysis (figs. 1 and 2), the most likely ancestral *secE*-*rpoBC*-*str*-*S10*-*spc*-*alpha* operon cluster was reconstructed for the last common ancestor of the *Gammaproteobacteria* and the last common ancestor of the *Bacilli* (fig. 3, supplementary tables S4 and S5, Supplementary Material online). Operons in the ancestral operon clusters were defined according to the *Escherichia coli* nomenclature to be consistent with previous studies. The *tufB* operon was absent from the operon cluster in all *Bacilli* but the ancestral operon cluster in the *Bacilli* contained two additional operons, the *cysS* operon upstream (*cysS*-*rnc*-*rlmB*-*orf1*-*sigH*) and the *ecf* operon downstream (*ecfA1*-*ecfA2*-*ecfT*-*truA*-*rplM*-*rpsI*) (fig. 3, supplementary table S5, Supplementary Material online). Overall, the two reconstructed ancestral operon clusters can be divided into a core cluster (*secE*-*rpoBC*-*str*-*S10*-*spc*-*alpha*) that is almost identical in both ancestors (40 out of 47 genes, 85% are present in both) and the variable tail operons (*tufB*, *cysS*, and *ecf*) that differ between the two ancestors (fig. 3). Interestingly, the outgroup species in the phylogenetic analysis of the *Gammaproteobacteria* strongly suggest that the *rpmG*, *adk*, *map* and *infA* genes were present in the *secE* and *spc* operons of the last common ancestor of the *Proteobacteria* (supplementary table S4, Supplementary Material online). Furthermore, the outgroup species in the phylogenetic analysis of the *Bacilli* suggest that the *tufB* operon was present in the last common ancestor of the *Firmicutes* (supplementary table S5, Supplementary Material online) which agrees with a previous study that suggests that the *tuf* duplication precedes the evolution of the *Firmicutes* (Lathe and Bork 2001). The potential ancestral operon cluster that was present in the last common ancestor of the *Proteobacteria* and the *Firmicutes* is shown in supplementary figure S1, Supplementary Material online.

### Identification of Evolutionary Events

The organization of the genes within the operon cluster of the 204 modern genomes included in this study was compared with their respective reconstructed ancestral organizations. All alterations within the organization were classified into two classes: deletions and rearrangement. “Deletions” were defined as alterations that remove a single gene from an operon but leave the operon–operon concatenation intact (the gene might be lost from the chromosome or effectively translocated), and “rearrangements” as changes that affect the operon structure and/or the operon–operon concatenation (inversions, gene insertions and loss of concatenation). Two species within the *Gammaproteobacteria* (*Legionella pneumophila* and *Halorhodospira halophila*) contained the full ancestral operon cluster and a further 17 species that belong to nine different orders only contained changes within the tRNA genes of the *tufB* operon. Overall, the seven operons of the ancestral operon cluster remained connected (<10 kb sequence between any two genes) in 38 out of 103 (37%) *Gammaproteobacteria* within thirteen orders (supplementary table S4, Supplementary Material online). Among the Bacilli, no modern species contained the full ancestral operon cluster but thirteen species among the *Bacillaceae* and *Thermoactinomycetaceae* differ only by a *rpsD* deletion and one species of the *Alicyclobacillaceae* differs only by an *ecfA1A2T* deletion from the ancestral operon cluster. The eight operons of the ancestral operon cluster remained connected in 34 out of 80 (43%) *Bacilli* within seven bacterial families (supplementary table S5, Supplementary Material online).

Next, the phylogenetic analysis was combined with the identified operon alterations to pinpoint evolutionary events that led to the modern operon organization. The four tRNA genes encoded in the *tufB* operon (*tRNA^Thr^*-*tRNA^Tyr^*-*tRNA^Gly^*-*tRNA^Thr^*) displayed a high degree of variability (e.g., frequent deletions of one or both *tRNA^Thr^* genes) and were therefore disregarded for this analysis. Overall, a total of 163 evolutionary events were identified, consisting of 48 deletions and 115 rearrangements (figs. 1 and 2, supplementary table S6, Supplementary Material online). The majority of the rearrangement events (75 out of 115, 65%) disrupt operon–operon concatenation but leave the operons intact. All operon pairs are affected except for the *spc*-*alpha* operon pair which remains concatenated in every genome that was analyzed. Two deletions and two rearrangements each were chosen from the *Gammaproteobacteria* and the *Bacilli* for further analysis (representing 10 independent events). For the deletions the focus was on deletions that are present in a small number of modern species and where the remaining operon organization is intact. Rearrangement events were selected based on the formation of conserved novel gene neighborhoods. Each of the eight deletions and rearrangements were tested for the influence of HGT from distantly related species that might transfer a rearranged operon structure into the chromosome (closely related species share the operon organization) and potential alternative mechanisms of operon cluster alterations are discussed.

### Deletions of *rplJL* (P23) and *rpmJ* (P50, P51, P52) within the *Gammaproteobacteria*

**Fig. 4.**
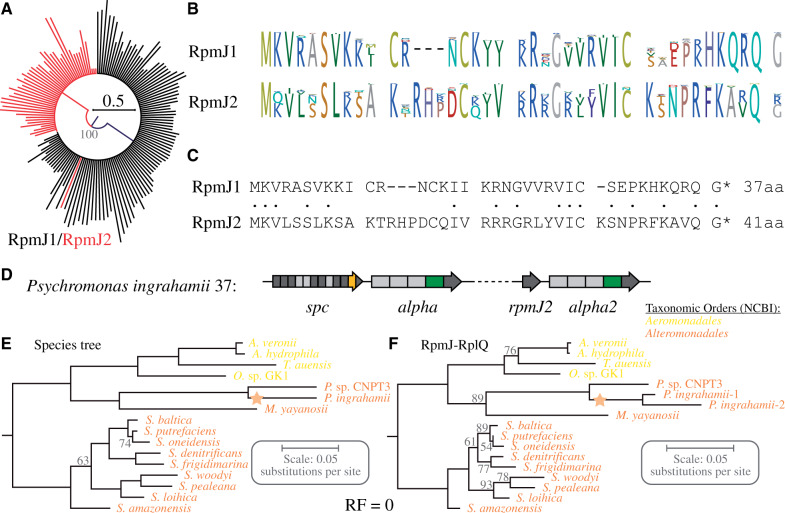
Analysis of *rpmJ* deletion events within the *Gammaproteobacteria* (events P50, P51, and P52). (*A*) Circular maximum likelihood phylogram (PhyML algorithm with WAG substitution model based on CLC alignments, nodes with bootstrap value below 80% were collapsed) of RpmJ proteins encoded within (RpmJ1, black) and outside (RpmJ2, red) the *spc* operon. (*B*) Logo of alignments of all RpmJ1 and all RpmJ2 proteins within the *Proteobacteria* and *Acidobacteria*. (*C*) Alignment of consensus sequences of RpmJ1 and RpmJ2 proteins within the *Proteobacteria* and *Acidobacteria*. Identical amino acids are indicated by a dot. (*D*) Organization of duplicate *spc*-*alpha* operons within *Psychromonas ingrahamii*. (*E*, *F*) Maximum likelihood phylogeny trees were produced using the PhyML algorithm (WAG substitution model) based on the concatenated CLC alignments of (*E*) 39 proteins within the *secE*-*rpoBC*-*str*-*S10*-*spc*-*alpha* operon cluster (supplementary table S2, Supplementary Material online) and (*F*) RpmJ, RpsM, RpsK, RpsD, RpoA, and RpsQ. Support for each node was evaluated by bootstrapping. The duplication event is indicated in the phylogenetic trees by a yellow star. RF value between the trees was calculated based on an 80% bootstrap threshold.

A single species within the *Gammaproteobacteria* (*Haemophilus influenzae* Rd KW20) carries a deletion (event P23) of the *rplJ* and *rplL* genes, encoding ribosomal proteins RplJ (L10) and RplL (L7), from the *rpoBC* operon (figs. 1 and 4). Both proteins are essential for bacterial growth (Baba et al. 2006) and the *H. influenzae* Rd KW20 genome contains a *rplJL* gene pair at a different genomic location. A test for HGT based on the Robinson–Foulds distance (Robinson and Foulds 1981) indicates that no HGT occurred from a distantly related species (RF = 0 based on an 80% bootstrap threshold, supplementary fig. S2, Supplementary Material online) leaving HGT from closely related species and intrachromosomal rearrangements as possible explanations for the *rplJL* deletion event.

The ribosomal protein RpmJ (L36) was deleted from the operon cluster in three independent events (P50, P51, and P52) resulting in six modern *Gammaproteobacteria* that lack the *rpmJ* gene within the operon cluster (fig. 1). RpmJ is not essential for bacterial growth in *E. coli* K-12 (Baba et al. 2006) indicating that the *Gammaproteobacteria* that lack the *rpmJ* gene within the operon cluster might produce RpmJ-free ribosomes. However, each of the six genomes contains a copy of the *rpmJ* gene outside the operon cluster. A screening of the genomes of all *Proteobacteria* and *Acidobacteria* included in this study revealed that 35% (40/115) of all genomes contain two copies of *rpmJ* (supplementary table S7, Supplementary Material online), suggesting a duplication or HGT event early in the evolution of the *Proteobacteria*. The *rpmJ* genes of the 115 *Proteobacteria* and *Acidobacteria* were classified as type 1 (*rpmJ1*, located within the *spc* operon) and type 2 (*rpmJ2*, located outside the *spc* operon) and a phylogenetic analysis was conducted to test if the *rpmJ* genes within the six modern species that lack the gene within the operon cluster are related to a novel duplication event (*rpmJ1*) or to the pre-existing duplication (*rpmJ2*). Only a single node that separates the *rpmJ1* and *rpmJ2* genes into two distinct clades displayed a bootstrap value >80% (with the exception of one *rpmJ2* gene which is discussed below, fig. 4*A*). The amino acid consensus sequence of the two *rpmJ* types shows 40% sequence identity and a three amino acid insertion that all *rpmJ2* genes share (fig. 4*B*, *C*). Two of the *rpmJ2* genes were identified in *Helicobacter pylori* J99 and *Campylobacter jejuni* 269.97, suggesting that the *rpmJ2* gene was present outside the operon cluster in the last common ancestor of the *Epsilonproteobacteria* and *Gammaproteobacteria*. Although this data do not reveal the origin of the *rpmJ2* gene (duplication or HGT), it shows that the three deletion events of the *rpmJ1* genes from the *spc* operon in the *Gammaproteobacteria* represent the deletion of a duplicate gene resulting in a genome with a single copy *rpmJ*.

A single *rpmJ2* gene (found in *Psychromonas ingrahamii*) was closely related to the *rpmJ1* genes (fig. 4*A*). Further analysis of the genomic location of the gene revealed that it was located together with a second copy of the *alpha* operon (*rpmJ2*-*rpsM2*-*rpsK2*-*rpsD2*-*rpoA2*-*rplQ2*) (fig. 4*D*). A phylogenetic analysis based on the six proteins indicates that the duplication is most likely a result of an intrachromosomal duplication event within the *P. ingrahamii* chromosome or was acquired by horizontal transfer from a closely related species (RF = 0 based on an 80% bootstrap threshold, fig. 4*E*, *F*).

### Deletions of *rpsN* (F81) and *secY* (F84) within the *Bacilli*

**Fig. 5.**
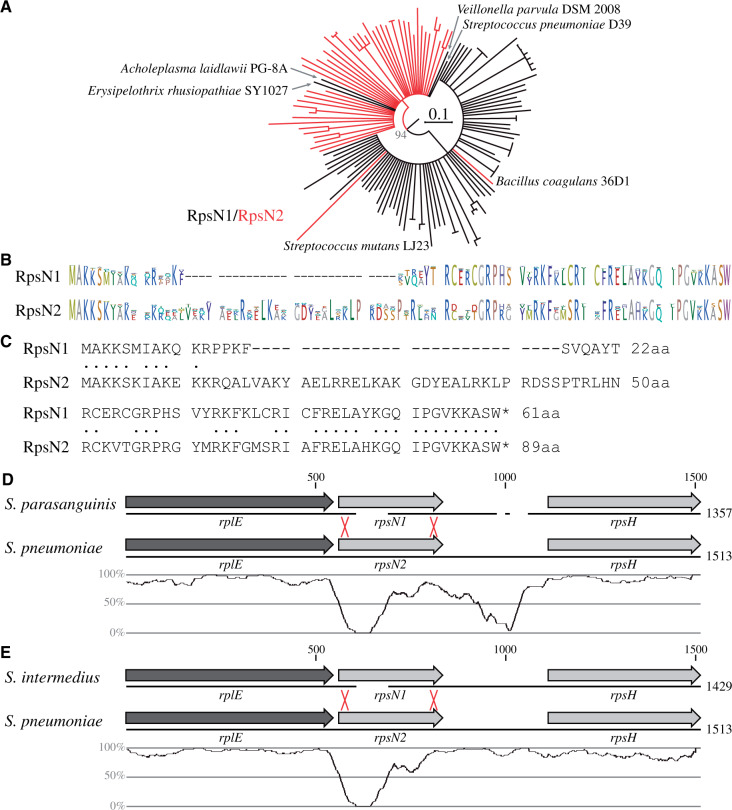
Analysis of *rpsN* deletion events within the *Bacilli* (events F81). (*A*) Circular maximum likelihood phylogram (CLC algorithm with WAG substitution model based on CLC alignments, nodes with bootstrap value below 80% were collapsed) of RpsN proteins encoded within (RpsN1, black) and outside (RpsN2, red) the *spc* operon. (*B*) Logo of alignments of all RpmJ1 and all RpmJ2 proteins within the *Firmicutes* and *Tenericutes*. (*C*) Alignment of consensus sequences of RpsN1 and RpsN2 proteins within the *Firmicutes* and *Tenericutes*. Identical amino acids are indicated by a dot. (*D*, *E*) Nucleotide sequence alignment of the *rplE*-*rpsN*-*rpsH* segment in the *spc* operon of *Streptococcus pneumoniae* with the corresponding segments in (*D*) *Streptococcus parasanguinis* and (*E*) *Streptococcus intermedius*. The black line indicates the DNA and coding sequences are shown above. The nucleotide sequence identity (averaged over 50 nt) is shown below. The red crosses indicate the likely location of a recombination event that inserted the *rpsN2* gene into the spc operon of *S*. *pneumoniae*.

The species within the *Leuconostocaceae* clade are the only *Bacilli* that lack the *rpsN* gene within the *spc* operon of the operon cluster (event F81). The *rpsN* gene encodes ribosomal protein RpsN (S14) that is essential for growth in *E. coli* (Baba et al. 2006). As expected, each of the four species within the *Leuconostocaceae* clade carry a copy of *rpsN* in the chromosome outside the *spc* operon. Screening the *Firmicutes* and *Tenericutes* included in this study for second copies of the *rpsN* gene showed that 46 out of the 89 genomes (52%) carry two copies of *rpsN* (supplementary table S8, Supplementary Material online). The *rpsN* genes within the *Firmicutes* and *Tenericutes* were classified as type 1 (*rpsN1*, located within the *spc* operon) and type 2 (*rpsN2*, located outside the *spc* operon) and a phylogenetic analysis based on the amino acid alignments was conducted (fig. 5). The results show two distinct versions of RpsN, a short isoform that is located within the *spc* operon (RpsN1, 31 aa) and a long isoform that is found outside the *spc* operon (RpsN2, 89 aa). There are six exceptions to this classification. Two species (*Bacillus coagulans* and *Streptococcus mutans*) carry a second copy of *rpsN1* outside the *spc* operon and four species carry the *rpsN2* isoform within the *spc* operon (fig. 5*A*). Interestingly, although three of the four species with the *rpsN2* isoform within the *spc* operon are members of the outgroup species (*Acholeplasma laidlawii*, *Erysipelothrix rhusiopathiae*, and *Veillonella parvula*) there is also a single species within the *Bacilli* (*Streptococcus pneumoniae*) that carries the longer *rpsN2* isoform within the *spc* operon. An alignment of the *rplE*-*rpsN*-*rpsH* segment of the *spc* operon within *S. pneumoniae* and the two closest related species (*Streptococcus parasanguinis* and *Streptococcus intermedius*) indicates that only the *rpsN* coding sequence was changed within *S. pneumoniae* whereas the neighboring genes remained unchanged (fig. 5*D*, *E*). The two RpsN isoforms display a high degree of protein sequence identity at the beginning (10 out of the first 11 amino acids) and the end (20 out of the last 21 amino acids) of the coding sequence (fig. 5*B*, *C*). Since the last common ancestor of the *Streptococcaceae* most likely carried both *rpsN* isoforms in its chromosome (supplementary table S8, Supplementary Material online) it is possible that the changed *rpsN* sequence in *S. pneumoniae* is the result of an intrachromosomal gene conversion event as seen for the duplicate *tuf* genes (Abdulkarim and Hughes 1996; Brandis et al. 2018). Alternatively, the novel *rpsN2* sequence within the *spc* operon of *S. pneumoniae* could be the result of an HGT event in which only the *rpsN* coding sequence was exchanged. Independent of the precise mechanism, these results show that the coding sequences of a single gene within the operon cluster can be exchanged to a significantly different isoform without affecting the neighboring genes or the intergenic sequences.

*Bacillus selenitireducens* is the only species within this study that has the *secY* gene, encoding the essential Sec translocon subunit SecY (Baba et al. 2006), located outside the *spc* operon (event F84). A phylogenetic analysis of the SecY proteins in the *Listeriaceae*, *Bacillaceae*, and *Sporolactobacillaceae* was conducted to test if the deletion of the *secY* gene from the *spc* operon in *B. selenitireducens* is the result of a long-distance HGT event. The resulting tree is fully compatible with the species tree of the respective species based on the full operon cluster (RF = 0 based on an 80% bootstrap threshold, supplementary fig. S3, Supplementary Material online). This indicates that no long-distance HGT event seemed to complement the deletion of the gene from the *spc* operon.

### Rearrangements of *rpoBC*-*Str* (P29) and *str*-*S10* (P38) within the *Gammaproteobacteria*

**Fig. 6.**
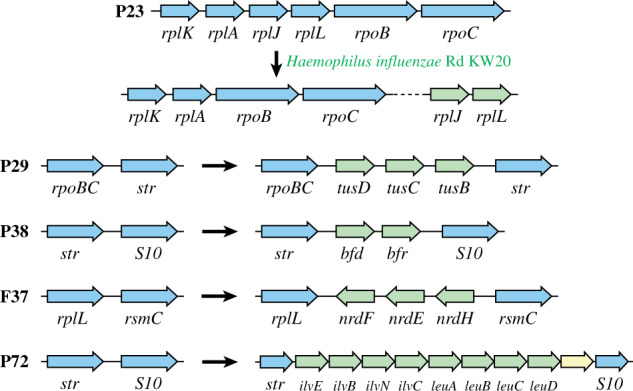
Overview over the four rearrangement events (P29, P38, F37, and F72) and one of the deletion events (P23) selected for further analysis. Genes/operons that are colinear at the time of the insertion are shown in blue and genes that represent the minimal inserted segment are green. The last gene of the insertion event F72 (yellow) is a putative amino acid transporter and was not part of the further analysis.

Two rearrangement events were chosen for the *Gammaproteobacteria* that most likely consisted of the insertion of a small number of genes between the operon pairs (fig. 6). The *tusDCB* genes were inserted between the *rpoBC* and *str* operon pair (event P29), and *bfd*-*bfr* between the *str* and *S10* operons (event P38). The *bfr* gene encodes a bacterioferritin that is responsible for iron storage within the bacterial cell (Andrews et al. 1989; Sevcenco et al. 2011) and *bfd* encodes a ferredoxin that binds Bfr and helps regulating iron homeostasis (Garg et al. 1996; Yao et al. 2012). The TusBCD sulfurtransferase complex is an important part of the 2-thiouridine synthesis of the modified wobble base mnm^5^s^2^U in tRNA (Ikeuchi et al. 2006; Numata et al. 2006). Based on the novel gene neighborhood present within the modern species, both insertion events most likely occurred within the last common ancestor of *Psychromonas*, *Moritella*, and all *Aeromondales*, *Vibrionales*, *Pateurellales*, *Orbales*, and *Enterobacterales* (fig. 1, supplementary figs. S4 and S5, Supplementary Material online). In many modern species, this novel gene order is not maintained but the operon pairs (*rpoBC*-*str* and *str*-*S10*) are fully disconnected (separated by >10 kb) suggesting that the gene insertions relaxed the selection for operon synteny (supplementary figs. S4*A* and S5*A*, Supplementary Material online). Phylogenetic analyses indicate that neither event is likely to be the result of a gene transfer event from a distantly related species (supplementary figs. S4 and S5, Supplementary Material online). The only node within the phylogenetic tree based on the TusDCB proteins that significantly differs from the species tree places *Moritella yayanosii* within the neighboring *Vibrionales* and *Aeromondales* clade but the node is only supported by a bootstrap value of 80% and the tree is still fully consistent with the insertion event (supplementary fig. S4, Supplementary Material online).

### Rearrangements of *rplL*-*rsmC* (F37) and *str*-*S10* (F72) within the *Bacilli*

The rearrangement events within the *Bacilli* that were chosen for further analysis occurred within the *Lactobacillaceae* (event F37) and within the *Sporolactobacillaceae* (event F72). Both events consist of insertions of a small number of genes (fig. 6). The *nrdHEF* genes were inserted between the *rplL* and *rsmC* genes within the *rpoBC* operon (event F37) and a set of amino acid biosynthesis genes, *ilvEBNC*-*leuABCD* between the *str* and *S10* operons (event F72). NrdH is a glutaredoxin-like protein that acts as an electron donor for the class Ib ribonucleotide reductase NrdEF which is involved in the biosynthesis of dNTPs (Jordan et al. 1994), and the IlvBCEN and LeuABCD proteins are part of the biosynthetic synthesis pathway of branched chain amino acids (Salmon et al. 2006). As seen for the insertion events within the *Gammaproteobacteria*, the novel gene order created by the insertion of the *nrdHEF* genes is not maintained in all modern species and the *rplL* and *rsmC* gene pair is fully disconnected in two out of the five modern species (supplementary fig. S6*A*, Supplementary Material online). The phylogenetic analysis based on the NrdHEF proteins shows no indications of HGT from a distantly related species (RF = 0 based on an 80% bootstrap threshold, supplementary fig. S6, Supplementary Material online) but the tree based on the IlvBCEN and LeuABCD proteins shows some deviations from the species tree (RF = 5 based on an 80% bootstrap threshold). However, all discrepancies are located within the *Bacillaceae* and *Sporolactobacillaceae* clades. The *Planococcaceae* clade in which the insertion event occurred is identical to the species tree (supplementary fig. S7, Supplementary Material online). It is therefore unlikely that either of the two events is the result of a long-distance HGT event.

### Potential Mechanisms That Could Drive Operon Cluster Alterations

The four deletions that were part of the further analysis were either 1) deletions of genes with a homolog in a different chromosomal location that has most likely been present in the last common ancestors of the *Gammaproteobacteria* (*rpmJ*, events P50, P51, and P52) or the *Bacilli* (*rpsN*, event F81), or 2) the result of a novel duplication (intrachromosomal or by HGT from a closely related species) followed by a deletion of the gene within the operon cluster (*rplJL*, event P23 and *secY*, event F84). For the rearrangement events the analysis indicates that the disconnection of two concatenated operons often follows the same pattern: initially, a small number of genes invade the interoperon region of the operon pair thus breaking the concatenation. Due to the relaxed selection for operon colocalization further rearrangement events can fully separate the operons. This also agrees with the observation that 67% of the events (77 out of 115 events) can be traced back to gene insertions (supplementary table S6, Supplementary Material online).

**Fig. 7.**
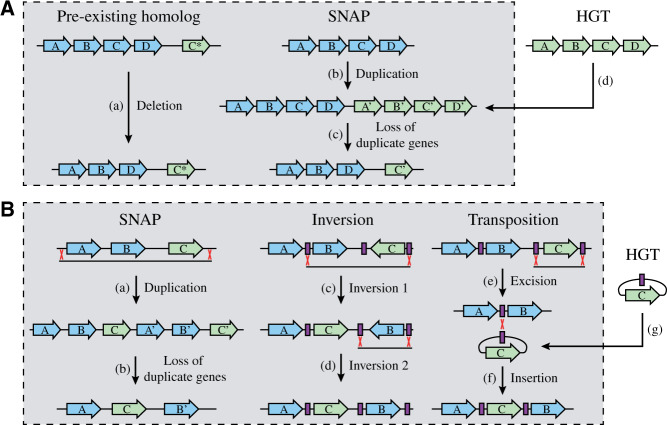
Potential mechanisms for operon cluster alteration events by intrachromosomal recombination (gray boxes) or horizontal gene transfer. (*A*) Genes can be deleted from the operon cluster if the chromosome contains a pre-existing homolog (a). Alternatively, the acquisition of a duplicate gene by intrachromosomal duplication (b) or horizontal gene transfer (d) allows for the deletion of the duplicate gene within the operon cluster (c). (*B*) Insertion of genes into the operon cluster could be facilitated by an intrachromosomal duplication event (a) followed by asymmetrical loss of duplicate genes (b), by two consecutive transposon-directed inversions (c, d), by transposon-directed transposition (e, f), or horizontal gene transfer (g) followed by transposon-directed insertion (f).

A potential mechanism that could explain each of the events that did not involve a pre-existing gene duplication is the recently suggested SNAP (Selection during Niche Adaptation) model. This model proposes that a segmental chromosomal duplication is selected and stabilized within a bacterial population during adaptation to a novel environment. Subsequently, the asymmetrical loss of duplicate genes can lead to alterations in the gene order on the chromosome (fig. 7) (Brandis and Hughes 2020). Segmental duplications are probably the most common type of mutations in the bacterial genome as they appear at very high frequencies even in the absence of selection (Anderson and Roth 1977; Haack and Roth 1995; Reams et al. 2010), are often found during laboratory selection conditions (Riehle et al. 2001; Knöppel et al. 2016), and play a role in shaping genome diversification and the evolution of new genes (Bergthorsson et al. 2007; Näsvall et al. 2012; Zhou et al. 2012). Additionally, a duplication of a chromosomal region would not disrupt operon integrity or operon concatenations. The subsequent loss of duplicate genes/operons is a slow process enabling the bacteria to adapt to potential negative consequences caused by the novel gene order. Thus, the SNAP model could explain the observed operon cluster alterations by combining high-frequency events and overcoming counter-selective barriers (Brandis and Hughes 2020). This model could also explain the duplicate section of the operon cluster (*rpmJ*-*rpsM*-*rpsK*-*rpsD*-*rpoA*-*rplQ*) found in *P*. *ingrahamii* (supplementary fig. S3*D*, Supplementary Material online). Alternatively, the gene insertion events could be facilitated by direct transposition, be the net outcome of multiple consecutive inversion events, or the result of the integration of a horizontally acquired segment from a closely related species (fig. 7*B*). A limitation with these three mechanisms is that they would be based on recombination between very short or nonhomologous sequences and are therefore expected to occur, if at all, at very low frequencies (Watt et al. 1985; Shen and Huang 1986; Brandis et al. 2018). Additionally, each of them leads to the sudden disruption of the operon integrity or operon concatenations and will most likely cause a reduction of cellular fitness (Campo et al. 2004; Brandis et al. 2019). Thus, these mechanisms couple a very infrequent event with a counter-selected fitness cost. A potential way to overcome these limitations would be “hijacking” of transposable elements. The integration of a transposon within or between operons could provide adequate sequence homologies for recombination that could ultimately lead to the insertion of genes between the operons. Transposon-directed integration is for example frequently observed with the integration of the F plasmid into the chromosome (Chumley et al. 1979). Interestingly, six of the events observed in the *Gammaproteobacteria* (P46) and *Bacilli* (F23, F33, F47, F76, and F103) consisted of integrations of transposable elements within the operon cluster (supplementary table S6, Supplementary Material online). This indicates that transposable elements could play a crucial role in the rearrangements of the operon cluster.

It is not possible to determine the precise mechanisms by which the analyzed operon cluster alterations occurred but the data suggest that duplications and transposable elements could be involved. Thus, deletions are most likely the result of a pre-existing ancient duplication or a novel duplication event that occurred intrachromosomally or by HGT from a closely related species (fig. 7*A*). Insertion events are most likely the result of a segmental duplication followed by asymmetrical loss of duplicate genes, transposon-directed intrachromosomal rearrangements, or transposon-directed integration of a horizontally acquired segment from a closely related species (fig. 7*B*).

### Operon Cluster Alteration Events Are Not Equally Distributed

The selection to maintain the linear gene order within bacterial chromosomes is weak on an evolutionary time scale (Tamames 2001). The *secE*-*rpoBC*-*str*-*S10*-*spc*-*alpha* operon cluster is a remarkable example of an exception to this rule as can be seen in species that maintained the full operon cluster organization over 2 Gy. This is further highlighted by the observation that changes to the operon cluster occur infrequently. Almost all branches within the two trees display no (70%) or single (20%) alteration events (supplementary table S9, Supplementary Material online). Interestingly, there are three branches within the *Bacilli* with seven or eight “simultaneous” events that might represent time points with unusually many operon cluster alteration events. To test this hypothesis, the ratio of alteration events per amino acid substitution (based on branch length) was calculated for every branch with at least one alteration in the *Gammaproteobacteria* and *Bacilli* trees. On average, there were 3.7 ± 3.8 alteration events per 1,000 amino acid substitutions with no significant difference (*P *=* *0.37, *t*-test) between the *Gammaproteobacteria* (3.3 ± 3.6 alterations per 1,000 substitutions) and the *Bacilli* (4.0 ± 3.9 alterations per 1,000 substitutions). Two of the three branches with a large number of alteration events were indistinguishable from this average rate (3.4 and 4.9 alterations per 1,000 substitutions) but the branch that represents the last common ancestor of the *Streptococcaceae* displayed a 3-fold increased operon alteration rate of 12 alterations per 1,000 amino acid substitutions. This indicates that the last common ancestor of the *Streptococcaceae* encountered a period with relaxed selection on the gene order within operon cluster. Another noteworthy observation is that a third of the events identified within the *Gammaproteobacteria* (33%, 17 out of 51 events) are located within the *Pasteurellales* whereas these only represent 12% of the analyzed species (fig. 1). An order-wide operon cluster alteration rate (including all branches and alterations unique to each order) was determined within the *Gammaproteobacteria* (supplementary table S10, Supplementary Material online). The alteration rate within the *Pasteurellales* was 3.24 alterations per 1,000 substitutions. This rate was 14-fold higher that the average rate of all other orders (0.24 ± 0.24 alterations per 1,000 substitutions). A possible explanation for this observation might be that the *Pasteurellales* are naturally competent. Natural competence per se is not uncommon among the *Gammaproteobacteria* (Johnston et al. 2014) but the *Pasteurellales* have a strong preference in the uptake of conspecific DNA (Bakkali et al. 2004; Redfield et al. 2006; Mell and Redfield 2014). It has been shown that natural transformation of conspecific DNA frequently generates duplications within bacterial genomes (Johnston et al. 2013). It is therefore possible that the natural competence coupled with preferential uptake of conspecific DNA could provide an increase of duplication formations that is ultimately responsible for the observed increase in operon rearrangements (fig. 7*A*). Alternatively, direct integration of the conspecific DNA into the chromosome could result in operon cluster alterations that would be indistinguishable from intrachromosomal translocations (fig. 7*B*).

## Conclusions

Here, the evolutionary history of the *secE*-*rpoBC*-*str*-*S10*-*spc*-*alpha* operon cluster was reconstructed for a period of 2 Gy back to the last common ancestors of the *Gammaproteobacteria* and the *Bacilli*. A total of 163 independent evolutionary events were identified in which the operon cluster was altered (figs. 1 and 2) and a subset of gene deletion and operon rearrangement events were further analyzed. The main findings were:

- The operon cluster remains stable in many *Gammaproteobacteria* and *Bacilli* over billions of years with no or little change.
- Single genes within the operon cluster can be exchanged with distantly related homologs without affecting the neighboring genes.
- Segmental duplications of the operon cluster can be found within the chromosomes.
- Deletions of genes within the operon cluster are the result of novel duplications or pre-excising homologs.
- Operons concatenation is broken by an invasion-separation mechanism.
- Intrachromosomal duplications and/or transposon-directed recombination play a crucial role in the rearrangement of the operon cluster.
- Long-distance HGT does not play a major role in the events that were further investigated.
- There are examples of accumulation of operon cluster alterations at specific branch points or within specific bacterial orders.

## Supplementary Material

Supplementary data are available at *Genome Biology and Evolution* online.

## Data Availability

All genome sequences included in this study are publicly available in the NCBI database. Accession numbers are provided in supplementary table S1, Supplementary Material online.

## Supplementary Material

evab041_Supplementary_Data
