# Supplementary material for: Reconstructing the Evolutionary History of a Highly Conserved Operon Cluster in Gammaproteobacteria and Bacilli
Source: Genome Biol Evol. 2021 Mar 2;13(4):evab041. doi: 10.1093/gbe/evab041 (PMC8046335; doi:10.1093/gbe/evab041)
Supplement: evab041_Supplementary_Data [file evab041_supplementary_data.zip › 20210222_Supplementary_Information.pdf]

Supplementary Information

Reconstructing the Evolutionary History of a Highly Conserved Operon Cluster in *Gammaproteobacteria* and *Bacilli*

Gerrit Brandis

Department of Cell and Molecular Biology, Uppsala University, Biomedical Center, Box 582, SE-75123 Uppsala, Sweden

Corresponding author: Gerrit Brandis

E-mail: gerrit.brandis@icm.uu.se

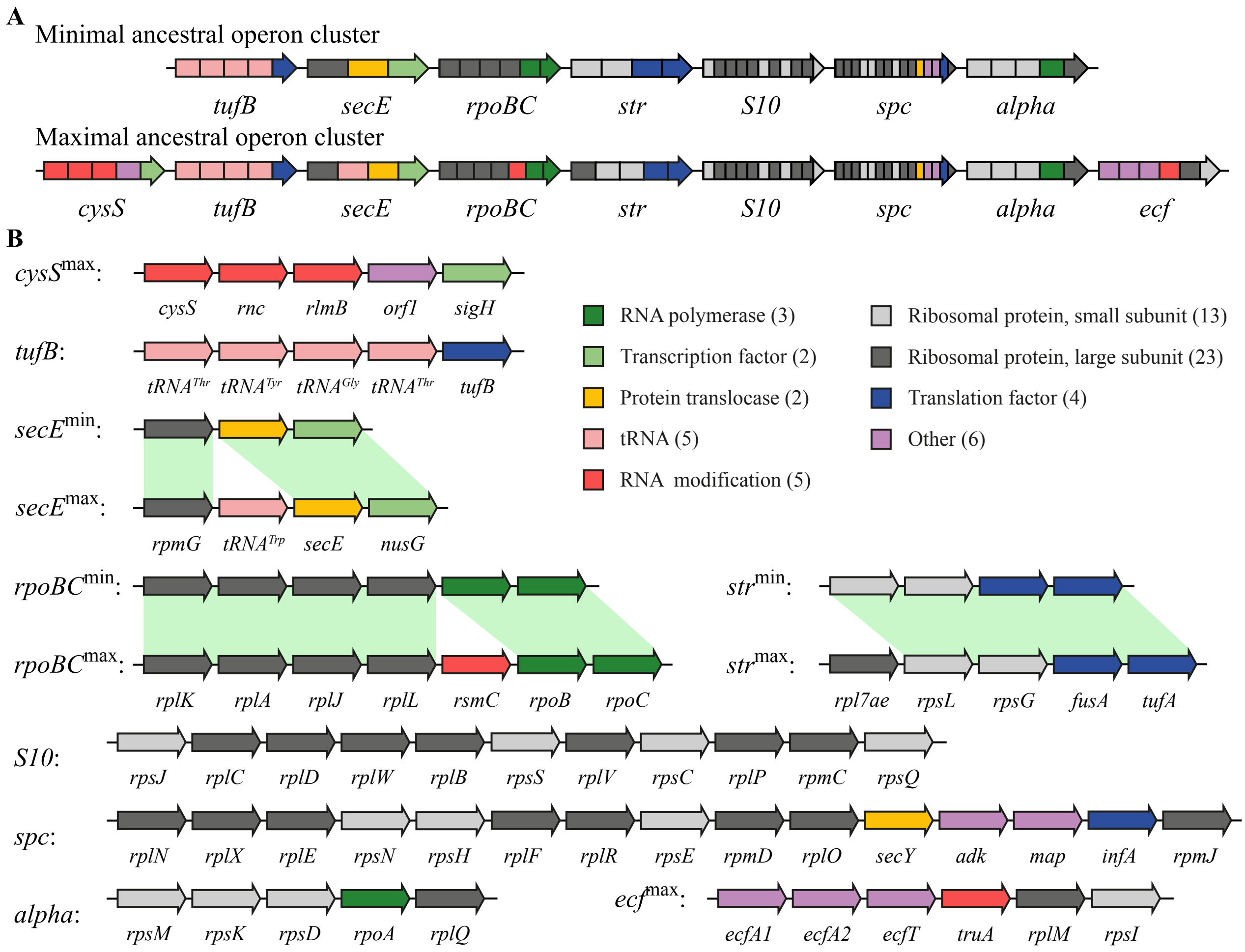

FIG. S1. Potential ancestral *secE-rpoBC-str-S10-spc-alpha* operon cluster in the last common ancestor of the *Proteobacteria*, *Acidobacteria*, *Firmicutes* and *Tenericutes*. (**A**) Overview over the operon concatenation. (**B**) Overview over the operon content. The minimal ancestral operon cluster (min) contains all operons/genes that are most likely to be present based on the analysis of the *Gammaproteobacteria* and *Bacilli*. The maximal ancestral operon cluster (max) additionally contains all ambiguous genes

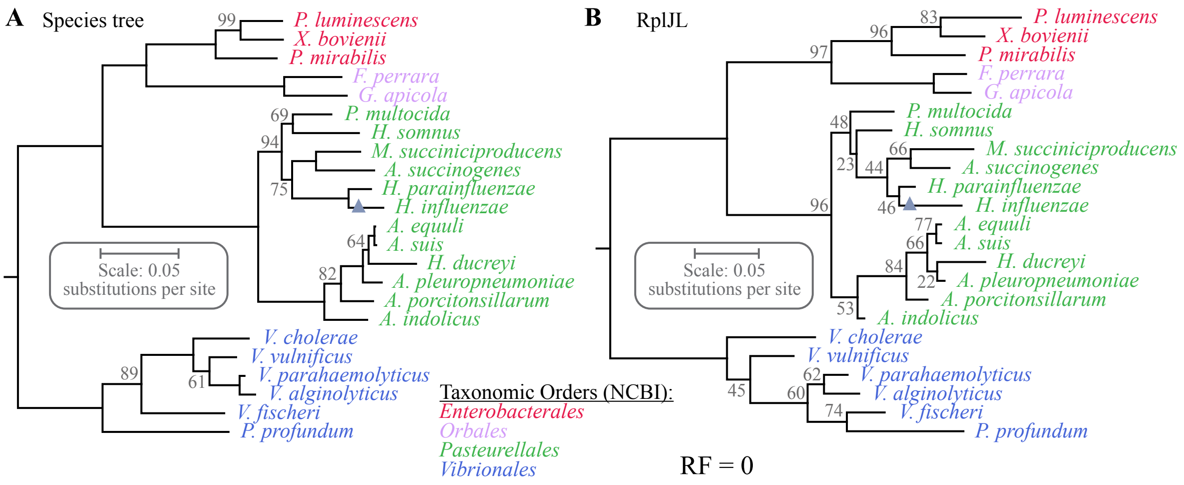

FIG. S2. Analysis of *rplJL* deletion in *Haemophilus influenzae* (event P23). Maximum likelihood phylogeny trees produced using the PhyML algorithm (WAG substitution model) based on the concatenated CLC alignments of (**A**) 39 proteins within the *secE-rpoBC-str-S10-spc-alpha* operon cluster (supplementary table S2) and (**B**) RplJ and RplL. Support for each node was evaluated by bootstrapping. The *rplJL* deletion event is indicated in the phylogenetic trees by a blue triangle. RF value between the trees was calculated based on an 80% bootstrap threshold.

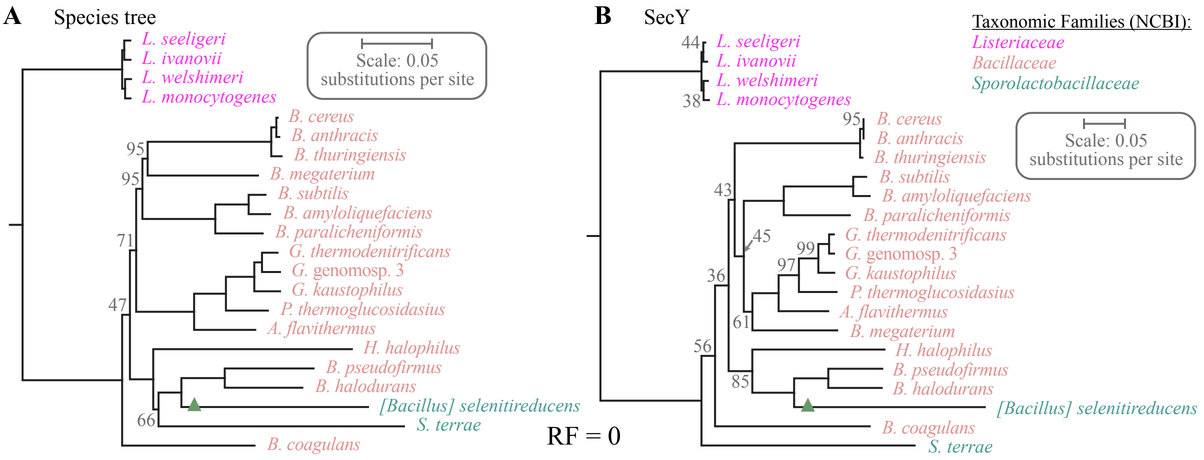

FIG. S3. Phylogenetic analysis of the *secY* deletion event within the *Bacilli* (event F84). Maximum likelihood phylogeny trees were produced using the CLC algorithm (WAG substitution model) based on the concatenated CLC alignments of (**A**) 44 proteins within the *secE-rpoBC-str-S10-spc-alpha* operon cluster (supplementary table S3) and (**B**) SecY. Support for each node was evaluated by bootstrapping and all support values for nodes are shown when these are below 100%. Taxonomic orders are designated according to NCBI and the time point of the *secY* deletion is indicated by a light green triangle. RF value between the trees was calculated based on an 80% bootstrap threshold.

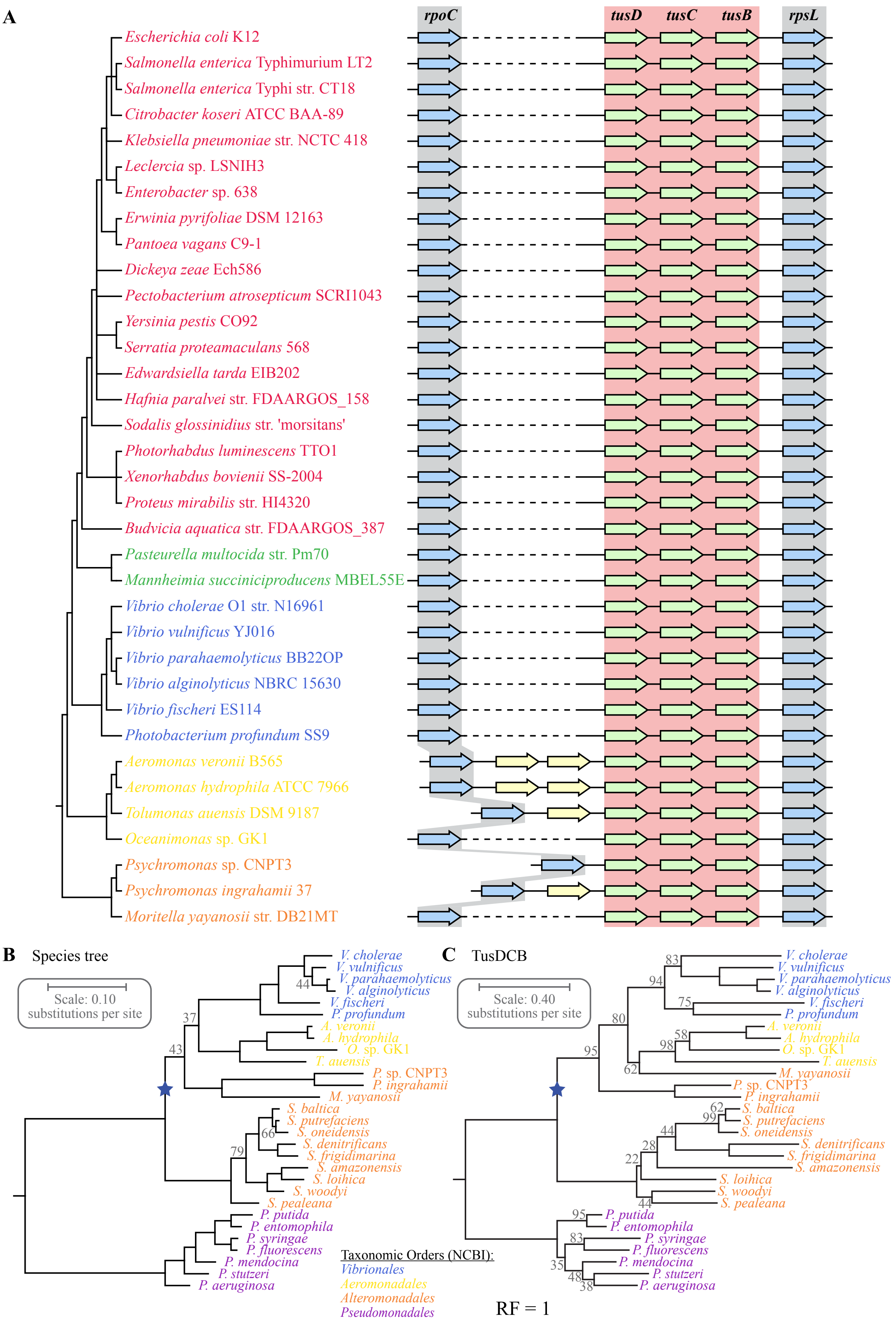

A
rpoC
tusD
tusC
tusB
rpsL
Escherichia coli K12
Salmonella enterica Typhimurium LT2
Salmonella enterica Typhi str. CT18
Citrobacter koseri ATCC BAA-89
Klebsiella pneumoniae str. NCTC 418
Leclercia sp. LSNIH3
Enterobacter sp. 638
Erwinia pyrifoliae DSM 12163
Pantoea vagans C9-1
Dickeya zeae Ech586
Pectobacterium atrosepticum SCRI1043
Yersinia pestis CO92
Serratia proteamaculans 568
Edwardsiella tarda EIB202
Hafnia paralvei str. FDAARGOS_158
Sodalis glossinidius str. 'morsitans'
Photorhabdus luminescens TTO1
Xenorhabdus bovienii SS-2004
Proteus mirabilis str. HI4320
Budvicia aquatica str. FDAARGOS_387
Pasteurella multocida str. Pm70
Mannheimia succiniciproducens MBEL55E
Vibrio cholerae O1 str. N16961
Vibrio vulnificus YJ016
Vibrio parahaemolyticus BB22OP
Vibrio alginolyticus NBRC 15630
Vibrio fischeri ES114
Photobacterium profundum SS9
Aeromonas veronii B565
Aeromonas hydrophila ATCC 7966
Tolumonas auensis DSM 9187
Oceanimonas sp. GK1
Psychromonas sp. CNPT3
Psychromonas ingrahamii 37
Moritella yayanosii str. DB21MT
B Species tree
Scale: 0.10 substitutions per site
C TusDCB
Scale: 0.40 substitutions per site
Taxonomic Orders (NCBI):
Vibrionales
Aeromonadales
Alteromonadales
Pseudomonadales
RF = 1

FIG. S4. Analysis of the *tusDCB* insertion event within the *Gammaproteobacteria* (event P29). (**A**) Genes that are co-linear at the time point of the insertion (*rpoC-rpsL*) are shown in blue, genes that represent the minimal inserted segment are green and genes that are not shared across the analysed species are yellow. Dashed lines indicate full disconnection (>10 kb distance) of the flanking genes and the gene orientation of disconnected gene pairs is not representative of the genomic organization. Phylogenetic relationship and taxonomic orders are indicated as in figure 1. Maximum likelihood phylogeny trees were produced using the PhyML algorithm (WAG substitution model) based on the concatenated CLC alignments of (**B**) 39 proteins within the *secE-rpoBC-str-S10-spc-alpha* operon cluster (supplementary table S2) and (**C**) TusDCB. Support for each node was evaluated by bootstrapping and all support values for nodes are shown when these are below 100%. Taxonomic orders are designated according to NCBI and the time point of the *tusDCB* insertion is indicated by a dark blue star. RF value between the trees was calculated based on an 80% bootstrap threshold.

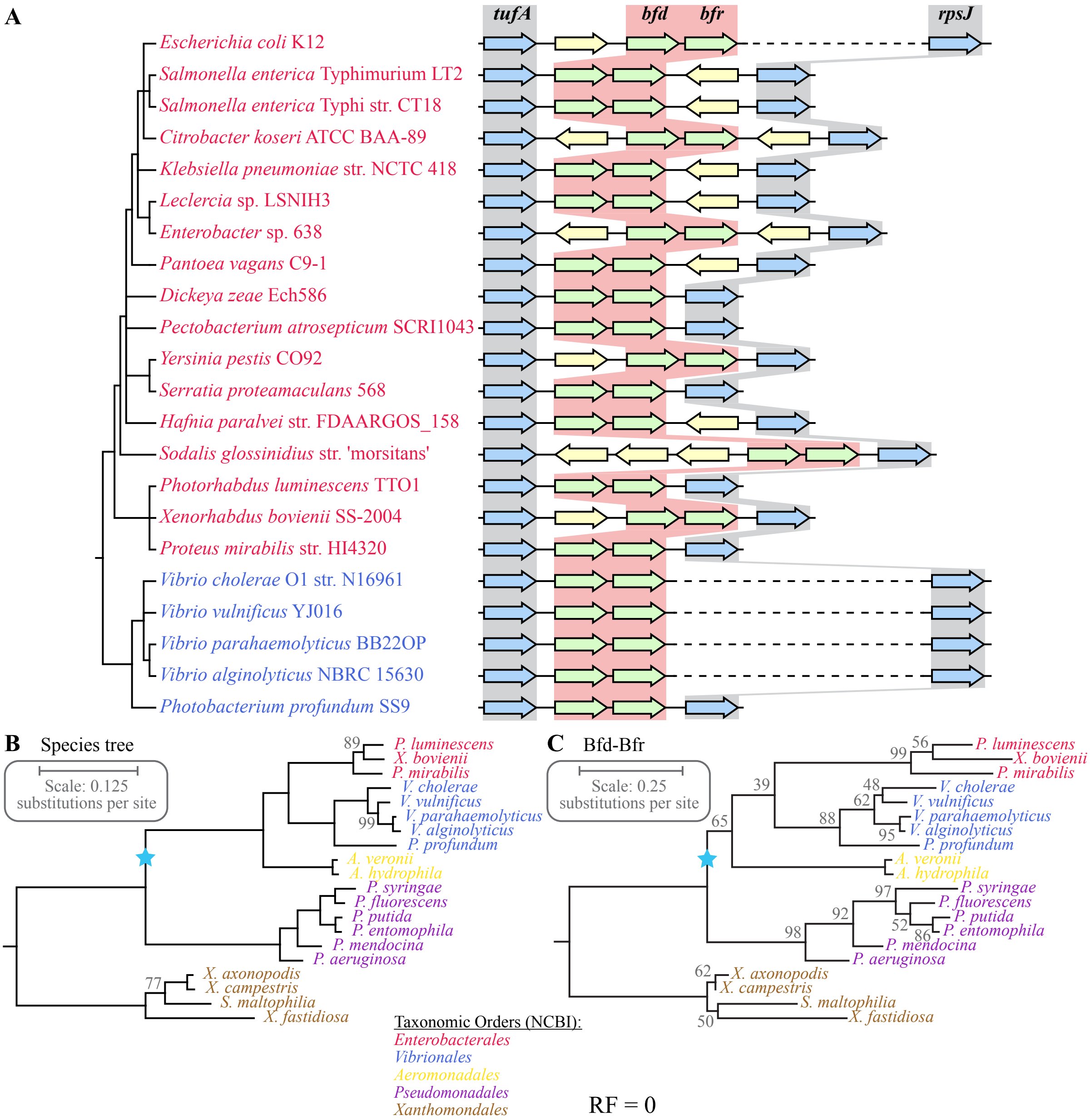

FIG. S5. Analysis of the *bfd-bfr* insertion event within the *Gammaproteobacteria* (event P38). (**A**) Genes that are co-linear at the time point of the insertion (*tufA-rpsJ*) are shown in blue, genes that represent the minimal inserted segment are green and genes that are not shared across the analysed species are yellow. Dashed lines indicate full disconnection (>10 kb distance) of the flanking genes and the gene orientation of disconnected gene pairs is not representative of the genomic organization. Phylogenetic relationship and taxonomic orders are indicated as in figure 1. Maximum likelihood phylogeny trees were produced using the PhyML algorithm (WAG substitution model) based on the concatenated CLC alignments of (**B**) 39 proteins within the *secE-rpoBC-str-S10-spc-alpha* operon cluster (supplementary table S2) and (**C**) Bfd and Bfr. Support for each node was evaluated by bootstrapping and all support values for nodes are shown when these are below 100%. Taxonomic orders are designated according to NCBI and the time point of the *bfd-bfr* insertion is indicated by a turquoise star. RF value between the trees was calculated based on an 80% bootstrap threshold.

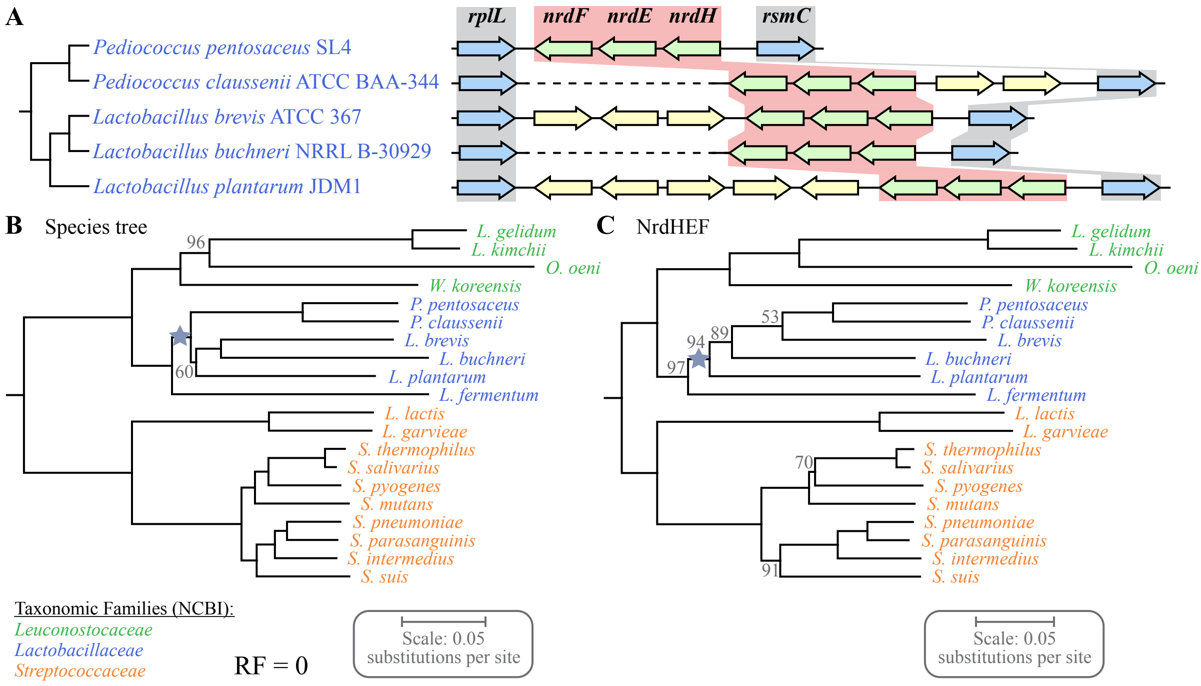

FIG. S6. Analysis of the *nrdHEF* insertion event within the *Bacilli* (event F37). (**A**) Genes that are co-linear at the time point of the insertion (*rplL-rsmC*) are shown in blue, genes that represent the minimal inserted segment are green and genes that are not shared across the analysed species are yellow. Dashed lines indicate full disconnection (>10 kb distance) of the flanking genes and the gene orientation of disconnected gene pairs is not representative of the genomic organization. Phylogenetic relationship and taxonomic orders are indicated as in figure 2. Maximum likelihood phylogeny trees were produced using the CLC algorithm (WAG substitution model) based on the concatenated CLC alignments of (**B**) 44 proteins within the *secE-rpoBC-str-S10-spc-alpha* operon cluster (supplementary table S3) and (**C**) NrdHEF. Support for each node was evaluated by bootstrapping and all support values for nodes are shown when these are below 100%. Taxonomic orders are designated according to NCBI and the time point of the *nrdHEF* insertion is indicated by a light blue star. RF value between the trees was calculated based on an 80% bootstrap threshold.

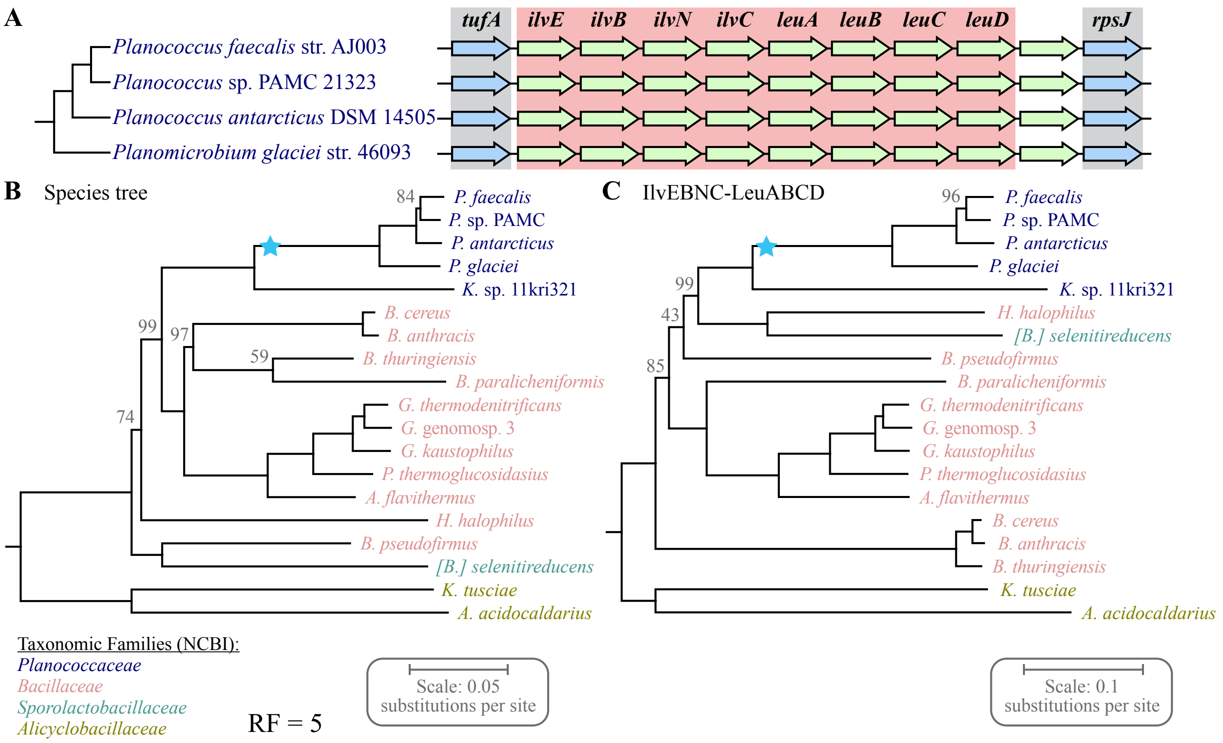

FIG. S7. Analysis of the *ilvEBNC-leuABCD* insertion event within the *Bacilli* (event F72). (**A**) Genes that are co-linear at the time point of the insertion (*tufA-rpsJ*) are shown in blue and the inserted genes are shown in green. The last gene of the insertion (not highlighted in red) is a putative amino acid transporter and was not part of the further analysis. Phylogenetic relationship and taxonomic families are indicated as in figure 2. Maximum likelihood phylogeny tree was produced using the CLC algorithm (WAG substitution model) based on the concatenated CLC alignments of (**B**) 44 proteins within the *secE-rpoBC-str-S10-spc-alpha* operon cluster (supplementary table S3) and (**C**) IlvEBNC-LeuABCD. Support for each node was evaluated by bootstrapping and all support values for nodes are shown when these are below 100%. Taxonomic orders are designated according to NCBI and the time point of the *ilvEBNC-leuABCD* insertion is indicated by a turquoise star. RF value between the trees was calculated based on an 80% bootstrap threshold.

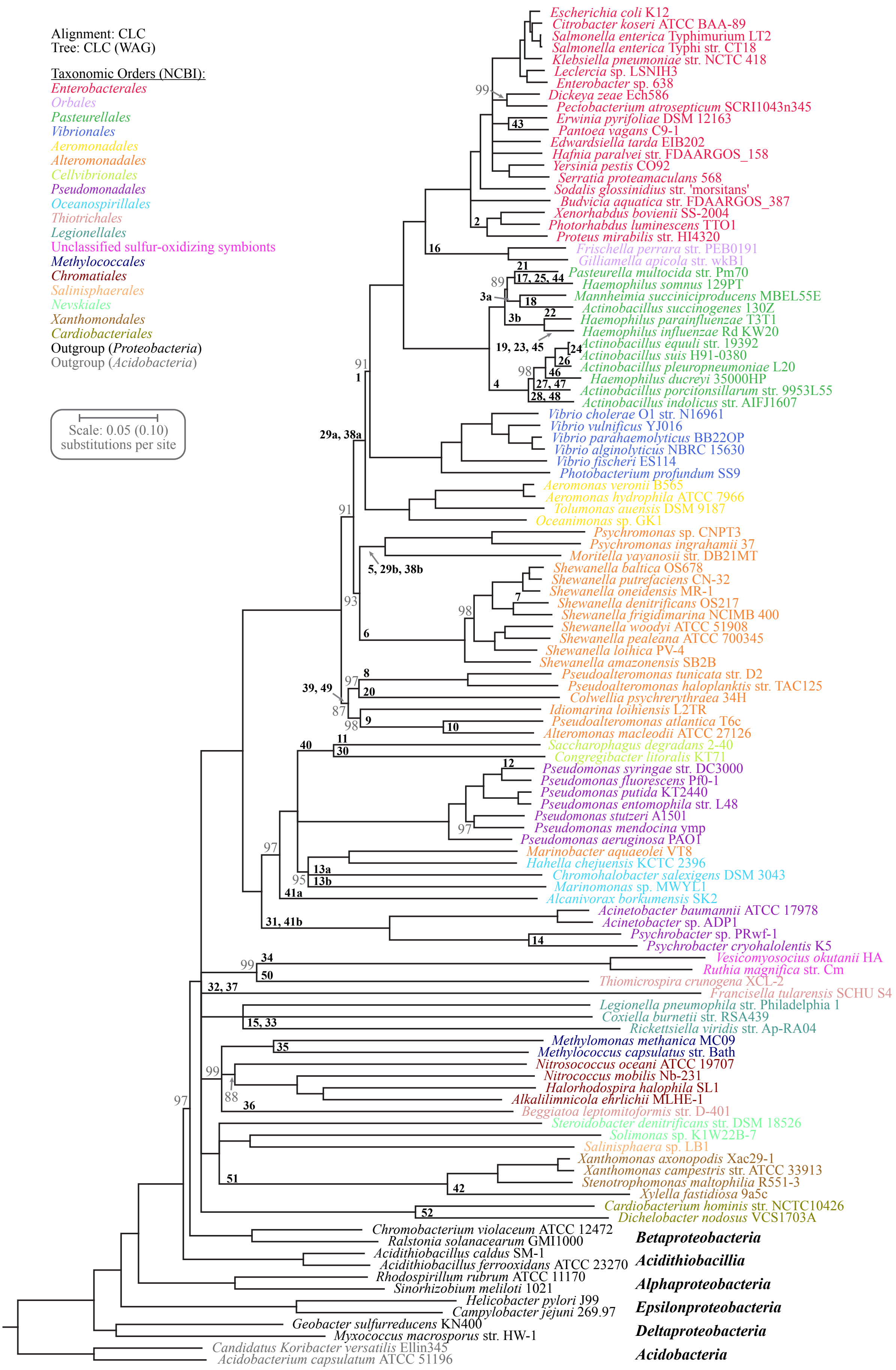

Alignment: CLC
Tree: CLC (WAG)
Taxonomic Orders (NCBI):
Enterobacterales
Orbales
Pasteurellales
Vibrionales
Aeromonadales
Alteromonadales
Cellvibrionales
Pseudomonadales
Oceanospirillales
Thiotrichales
Legionellales
Unclassified sulfur-oxidizing symbionts
Methylococcales
Chromatiales
Salinisphaerales
Nevskiales
Xanthomondales
Cardiobacteriales
Outgroup (Proteobacteria)
Outgroup (Acidobacteria)
Scale: 0.05 (0.10)
substitutions per site
Escherichia coli K12
Citrobacter koseri ATCC BAA-89
Salmonella enterica Typhimurium LT2
Salmonella enterica Typhi str. CT18
Klebsiella pneumoniae str. NCTC 418
Leclercia sp. LSNIH3
Enterobacter sp. 638
Dickeya zeae Ech586
Pectobacterium atrosepticum SCRI1043n345
Erwinia pyrifoliae DSM 12163
Pantoea vagans C9-1
Edwardsiella tarda EIB202
Hafnia paralvei str. FDAARGOS_158
Yersinia pestis CO92
Serratia proteamaculans 568
Sodalis glossinidius str. 'morsitans'
Budvicia aquatica str. FDAARGOS_387
Xenorhabdus bovienii SS-2004
Photorhabdus luminescens TTO1
Proteus mirabilis str. HI4320
Frischella perrara str. PEB0191
Gilliamella apicola str. wkB1
Pasteurella multocida str. Pm70
Haemophilus somnus 129PT
Mannheimia succiniciproducens MBEL55E
Actinobacillus succinogenes 130Z
Haemophilus parainfluenzae T3T1
Haemophilus influenzae Rd KW20
Actinobacillus equuli str. 19392
Actinobacillus suis H91-0380
Actinobacillus pleuropneumoniae L20
Haemophilus ducreyi 35000HP
Actinobacillus porcitonsillarum str. 9953L55
Actinobacillus indolicus str. AIFJ1607
Vibrio cholerae O1 str. N16961
Vibrio vulnificus YJ016
Vibrio parahaemolyticus BB22OP
Vibrio alginolyticus NBRC 15630
Vibrio fischeri ES114
Photobacterium profundum SS9
Aeromonas veronii B565
Aeromonas hydrophila ATCC 7966
Tolumonas auensis DSM 9187
Oceanimonas sp. GK1
Psychromonas sp. CNPT3
Psychromonas ingrahamii 37
Moritella yayanosii str. DB21MT
Shewanella baltica OS678
Shewanella putrefaciens CN-32
Shewanella oneidensis MR-1
Shewanella denitrificans OS217
Shewanella frigidimarina NCIMB 400
Shewanella woodyi ATCC 51908
Shewanella pealeana ATCC 700345
Shewanella loihica PV-4
Shewanella amazonensis SB2B
Pseudoalteromonas tunicata str. D2
Pseudoalteromonas haloplanktis str. TAC125
Colwellia psychrerythraea 34H
Idiomarina loihiensis L2TR
Pseudoalteromonas atlantica T6c
Alteromonas macleodii ATCC 27126
Saccharophagus degradans 2-40
Congregibacter litoralis KT71
Pseudomonas syringae str. DC3000
Pseudomonas fluorescens Pf0-1
Pseudomonas putida KT2440
Pseudomonas entomophila str. L48
Pseudomonas stutzeri A1501
Pseudomonas mendocina ymp
Pseudomonas aeruginosa PAO1
Marinobacter aquaeolei VT8
Hahella chejuensis KCTC 2396
Chromohalobacter salexigens DSM 3043
Marinomonas sp. MWYL1
Alcanivorax borkumensis SK2
Acinetobacter baumannii ATCC 17978
Acinetobacter sp. ADP1
Psychrobacter sp. PRwf-1
Psychrobacter cryohalolentis K5
Vesicomyosocius okutanii HA
Ruthia magnifica str. Cm
Thiomicrospira crunogena XCL-2
Francisella tularensis SCHU S4
Legionella pneumophila str. Philadelphia 1
Coxiella burnetii str. RSA439
Rickettsiella viridis str. Ap-RA04
Methylomonas methanica MC09
Methylococcus capsulatus str. Bath
Nitrosococcus oceani ATCC 19707
Nitrococcus mobilis Nb-231
Halorhodospira halophila SL1
Alkalilimnicola ehrlichii MLHE-1
Beggiatoa leptomitoformis str. D-401
Steroidobacter denitrificans str. DSM 18526
Solimonas sp. K1W22B-7
Salinisphaera sp. LB1
Xanthomonas axonopodis Xac29-1
Xanthomonas campestris str. ATCC 33913
Stenotrophomonas maltophilia R551-3
Xylella fastidiosa 9a5c
Cardiobacterium hominis str. NCTC10426
Dichelobacter nodosus VCS1703A
Chromobacterium violaceum ATCC 12472
Ralstonia solanacearum GMI1000
Acidithiobacillus caldus SM-1
Acidithiobacillus ferrooxidans ATCC 23270
Rhodospirillum rubrum ATCC 11170
Sinorhizobium meliloti 1021
Helicobacter pylori J99
Campylobacter jejuni 269.97
Geobacter sulfurreducens KN400
Myxococcus macrosporus str. HW-1
Candidatus Koribacter versatilis Ellin345
Acidobacterium capsulatum ATCC 51196
Betaproteobacteria
Acidithiobacillia
Alphaproteobacteria
Epsilonproteobacteria
Deltaproteobacteria
Acidobacteria

FIG. S8. Phylogeny of the *Gammaproteobacteria*. A maximum likelihood phylogeny tree was produced using the CLC algorithm (WAG substitution model) based on the concatenated CLC alignments of 39 proteins within the *secE-rpoBC-str-S10-spc-alpha* operon cluster (supplementary table S2). Support for each node was evaluated by bootstrapping and nodes with a bootstrap value below 80% were collapsed. Support values for nodes are shown when these are below 100%. Branch lengths for the outgroup species was reduced by a factor of two (see scale value in parenthesis). Taxonomic orders are designated according to NCBI and evolutionary events are indicated in the tree. Evolutionary events are indicated above the branches. See supplementary table S6 for event details.

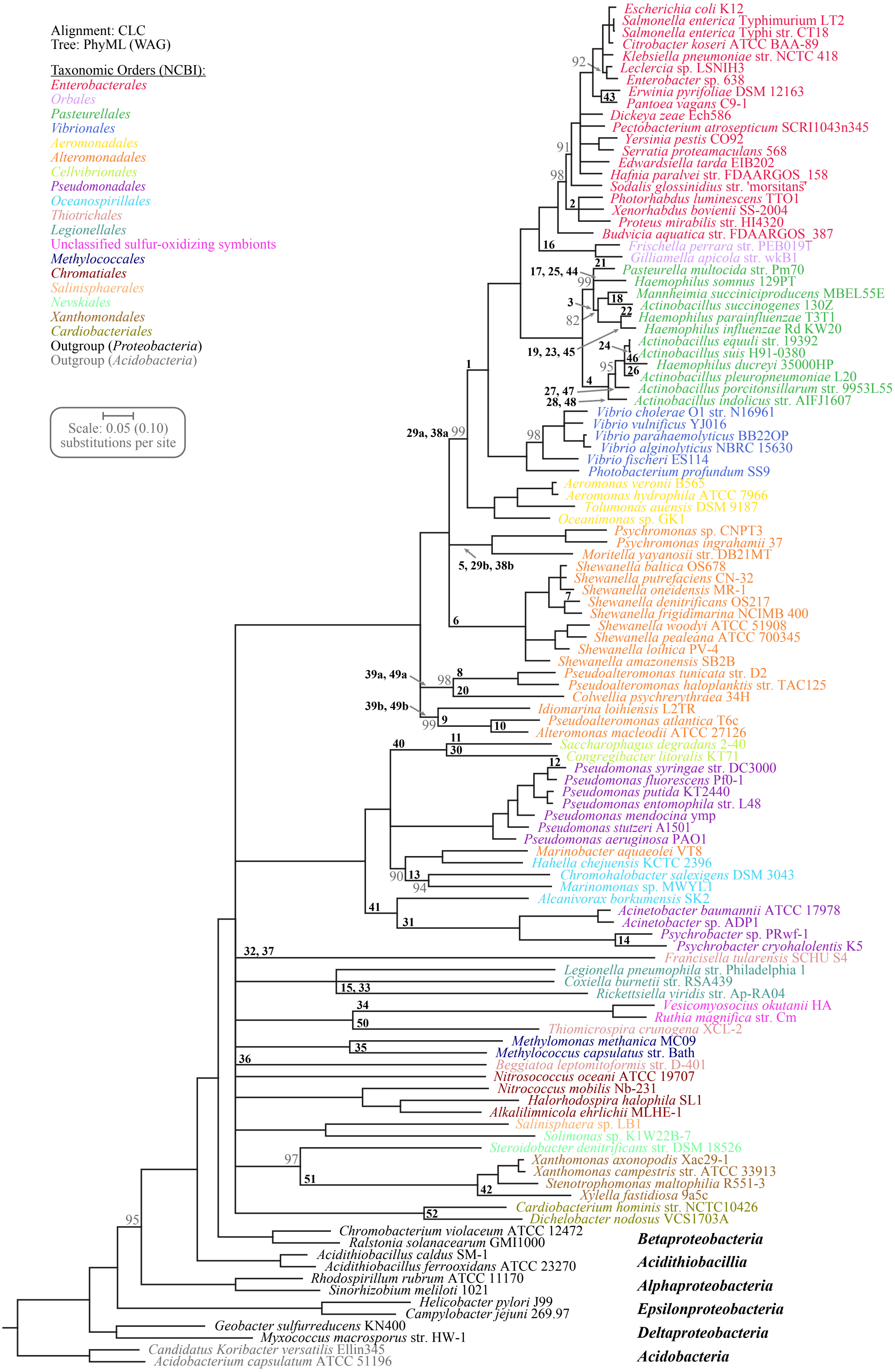

Alignment: CLC
Tree: PhyML (WAG)
Taxonomic Orders (NCBI):
Enterobacterales
Orbales
Pasteurellales
Vibrionales
Aeromonadales
Alteromonadales
Cellvibrionales
Pseudomonadales
Oceanospirillales
Thiotrichales
Legionellales
Unclassified sulfur-oxidizing symbionts
Methylococcales
Chromatiales
Salinisphaerales
Nevskiales
Xanthomondales
Cardiobacteriales
Outgroup (Proteobacteria)
Outgroup (Acidobacteria)
Scale: 0.05 (0.10) substitutions per site
Escherichia coli K12
Salmonella enterica Typhimurium LT2
Salmonella enterica Typhi str. CT18
Citrobacter koseri ATCC BAA-89
Klebsiella pneumoniae str. NCTC 418
Leclercia sp. LSNIH3
Enterobacter sp. 638
Erwinia pyrifoliae DSM 12163
Pantoea vagans C9-1
Dickeya zeae Ech586
Pectobacterium atrosepticum SCRI1043n345
Yersinia pestis CO92
Serratia proteamaculans 568
Edwardsiella tarda EIB202
Hafnia paralvei str. FDAARGOS_158
Sodalis glossinidius str. 'morsitans'
Photorhabdus luminescens TTO1
Xenorhabdus bovienii SS-2004
Proteus mirabilis str. HI4320
Budvicia aquatica str. FDAARGOS_387
Frischella perrara str. PEB0191
Gilliamella apicola str. wkB1
Pasteurella multocida str. Pm70
Haemophilus somnus 129PT
Mannheimia succiniciproducens MBEL55E
Actinobacillus succinogenes 130Z
Haemophilus parainfluenzae T3T1
Haemophilus influenzae Rd KW20
Actinobacillus equuli str. 19392
Actinobacillus suis H91-0380
Haemophilus ducreyi 35000HP
Actinobacillus pleuropneumoniae L20
Actinobacillus porcitonsillarum str. 9953L55
Actinobacillus indolicus str. AIFJ1607
Vibrio cholerae O1 str. N16961
Vibrio vulnificus YJ016
Vibrio parahaemolyticus BB22OP
Vibrio alginolyticus NBRC 15630
Vibrio fischeri ES114
Photobacterium profundum SS9
Aeromonas veronii B565
Aeromonas hydrophila ATCC 7966
Tolumonas auensis DSM 9187
Oceanimonas sp. GK1
Psychromonas sp. CNPT3
Psychromonas ingrahamii 37
Moritella yayanosii str. DB21MT
Shewanella baltica OS678
Shewanella putrefaciens CN-32
Shewanella oneidensis MR-1
Shewanella denitrificans OS217
Shewanella frigidimarina NCIMB 400
Shewanella woodyi ATCC 51908
Shewanella pealeana ATCC 700345
Shewanella loihica PV-4
Shewanella amazonensis SB2B
Pseudoalteromonas tunicata str. D2
Pseudoalteromonas haloplanktis str. TAC125
Colwellia psychrerythraea 34H
Idiomarina loihiensis L2TR
Pseudoalteromonas atlantica T6c
Alteromonas macleodii ATCC 27126
Saccharophagus degradans 2-40
Congregibacter litoralis KT71
Pseudomonas syringae str. DC3000
Pseudomonas fluorescens Pf0-1
Pseudomonas putida KT2440
Pseudomonas entomophila str. L48
Pseudomonas mendocina ymp
Pseudomonas stutzeri A1501
Pseudomonas aeruginosa PAO1
Marinobacter aquaeolei VT8
Hahella chejuensis KCTC 2396
Chromohalobacter salexigens DSM 3043
Marinomonas sp. MWYL1
Alcanivorax borkumensis SK2
Acinetobacter baumannii ATCC 17978
Acinetobacter sp. ADP1
Psychrobacter sp. PRwf-1
Psychrobacter cryohalolentis K5
Francisella tularensis SCHU S4
Legionella pneumophila str. Philadelphia 1
Coxiella burnetii str. RSA439
Rickettsiella viridis str. Ap-RA04
Vesicomyosocius okutanii HA
Ruthia magnifica str. Cm
Thiomicrospira crunogena XCL-2
Methylomonas methanica MC09
Methylococcus capsulatus str. Bath
Beggiatoa leptomitoformis str. D-401
Nitrosococcus oceani ATCC 19707
Nitrococcus mobilis Nb-231
Halorhodospira halophila SL1
Alkalilimnicola ehrlichii MLHE-1
Salinisphaera sp. LB1
Solimonas sp. K1W22B-7
Steroidobacter denitrificans str. DSM 18526
Xanthomonas axonopodis Xac29-1
Xanthomonas campestris str. ATCC 33913
Stenotrophomonas maltophilia R551-3
Xylella fastidiosa 9a5c
Cardiobacterium hominis str. NCTC10426
Dichelobacter nodosus VCS1703A
Chromobacterium violaceum ATCC 12472
Ralstonia solanacearum GMI1000
Acidithiobacillus caldus SM-1
Acidithiobacillus ferrooxidans ATCC 23270
Rhodospirillum rubrum ATCC 11170
Sinorhizobium meliloti 1021
Helicobacter pylori J99
Campylobacter jejuni 269.97
Geobacter sulfurreducens KN400
Myxococcus macrosporus str. HW-1
Candidatus Koribacter versatilis Ellin345
Acidobacterium capsulatum ATCC 51196
Betaproteobacteria
Acidithiobacillia
Alphaproteobacteria
Epsilonproteobacteria
Deltaproteobacteria
Acidobacteria

FIG. S9. Phylogeny of the *Gammaproteobacteria*. A maximum likelihood phylogeny tree was produced using the PhyML algorithm (WAG substitution model) based on the concatenated CLC alignments of 39 proteins within the *secE-rpoBC-str-S10-spc-alpha* operon cluster (supplementary table S2). Support for each node was evaluated by bootstrapping and nodes with a bootstrap value below 80% were collapsed. Support values for nodes are shown when these are below 100%. Branch lengths for the outgroup species was reduced by a factor of two (see scale value in parenthesis). Taxonomic orders are designated according to NCBI and evolutionary events are indicated in the tree. Evolutionary events are indicated above the branches. See supplementary table S6 for event details.

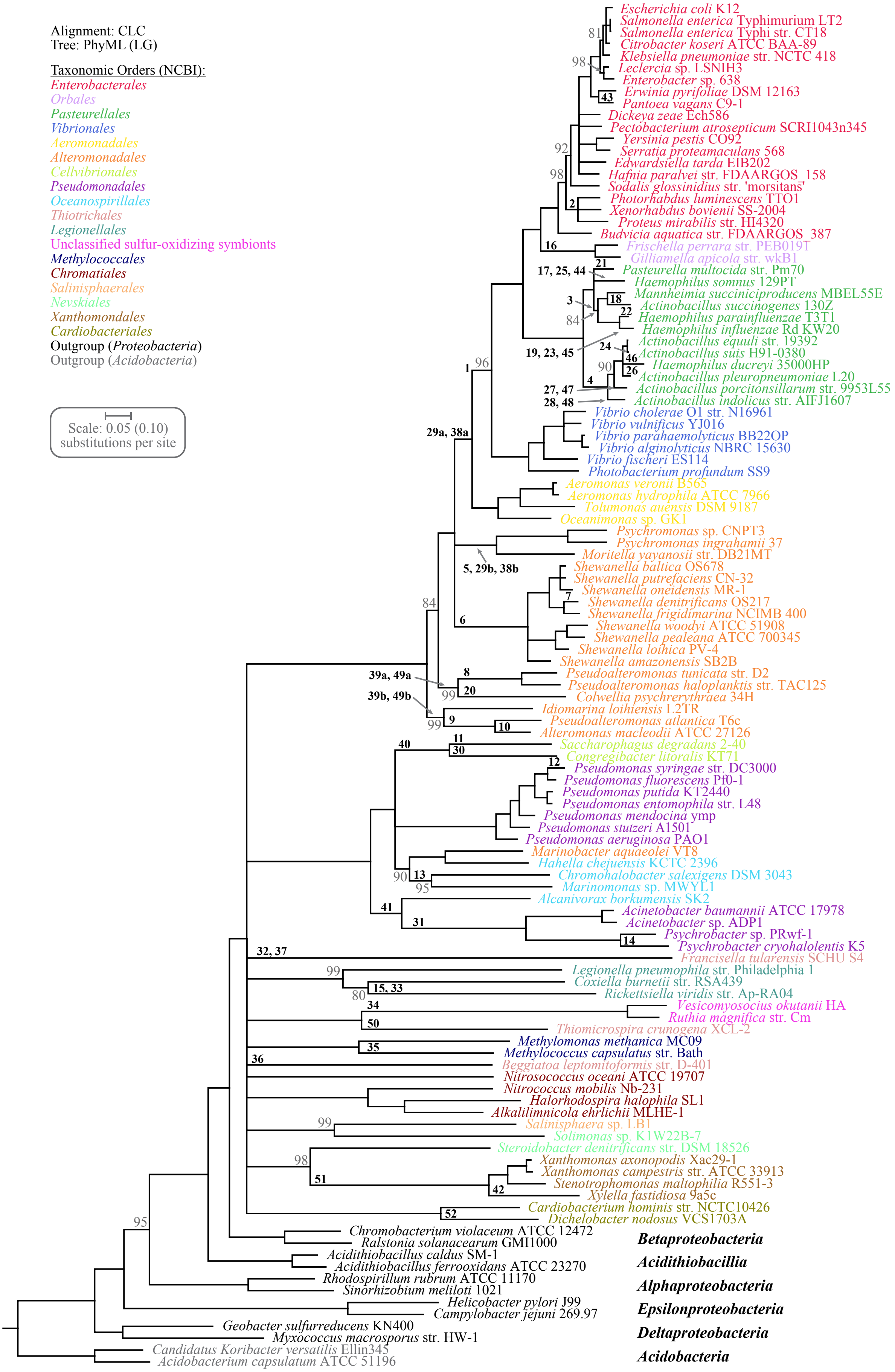

Alignment: CLC
Tree: PhyML (LG)
Taxonomic Orders (NCBI):
Enterobacterales
Orbales
Pasteurellales
Vibrionales
Aeromonadales
Alteromonadales
Cellvibrionales
Pseudomonadales
Oceanospirillales
Thiotrichales
Legionellales
Unclassified sulfur-oxidizing symbionts
Methylococcales
Chromatiales
Salinisphaerales
Nevskiales
Xanthomondales
Cardiobacteriales
Outgroup (Proteobacteria)
Outgroup (Acidobacteria)
Scale: 0.05 (0.10) substitutions per site
Escherichia coli K12
Salmonella enterica Typhimurium LT2
Salmonella enterica Typhi str. CT18
Citrobacter koseri ATCC BAA-89
Klebsiella pneumoniae str. NCTC 418
Leclercia sp. LSNIH3
Enterobacter sp. 638
Erwinia pyrifoliae DSM 12163
Pantoea vagans C9-1
Dickeya zeae Ech586
Pectobacterium atrosepticum SCRI1043n345
Yersinia pestis CO92
Serratia proteamaculans 568
Edwardsiella tarda EIB202
Hafnia paralvei str. FDAARGOS_158
Sodalis glossinidius str. 'morsitans'
Photorhabdus luminescens TTO1
Xenorhabdus bovienii SS-2004
Proteus mirabilis str. HI4320
Budvicia aquatica str. FDAARGOS_387
Frischella perrara str. PEB0191T
Gilliamella apicola str. wkB1
Pasteurella multocida str. Pm70
Haemophilus somnus 129PT
Mannheimia succiniciproducens MBEL55E
Actinobacillus succinogenes 130Z
Haemophilus parainfluenzae T3T1
Haemophilus influenzae Rd KW20
Actinobacillus equuli str. 19392
Actinobacillus suis H91-0380
Haemophilus ducreyi 35000HP
Actinobacillus pleuropneumoniae L20
Actinobacillus porcitonsillarum str. 9953L55
Actinobacillus indolicus str. AIFJ1607
Vibrio cholerae O1 str. N16961
Vibrio vulnificus YJ016
Vibrio parahaemolyticus BB22OP
Vibrio alginolyticus NBRC 15630
Vibrio fischeri ES114
Photobacterium profundum SS9
Aeromonas veronii B565
Aeromonas hydrophila ATCC 7966
Tolumonas auensis DSM 9187
Oceanimonas sp. GK1
Psychromonas sp. CNPT3
Psychromonas ingrahamii 37
Moritella yayanosii str. DB21MT
Shewanella baltica OS678
Shewanella putrefaciens CN-32
Shewanella oneidensis MR-1
Shewanella denitrificans OS217
Shewanella frigidimarina NCIMB 400
Shewanella woodyi ATCC 51908
Shewanella pealeana ATCC 700345
Shewanella loihica PV-4
Shewanella amazonensis SB2B
Pseudoalteromonas tunicata str. D2
Pseudoalteromonas haloplanktis str. TAC125
Colwellia psychrerythraea 34H
Idiomarina loihiensis L2TR
Pseudoalteromonas atlantica T6c
Alteromonas macleodii ATCC 27126
Saccharophagus degradans 2-40
Congregibacter litoralis KT71
Pseudomonas syringae str. DC3000
Pseudomonas fluorescens Pf0-1
Pseudomonas putida KT2440
Pseudomonas entomophila str. L48
Pseudomonas mendocina ymp
Pseudomonas stutzeri A1501
Pseudomonas aeruginosa PAO1
Marinobacter aquaeolei VT8
Hahella chejuensis KCTC 2396
Chromohalobacter salexigens DSM 3043
Marinomonas sp. MWYL1
Alcanivorax borkumensis SK2
Acinetobacter baumannii ATCC 17978
Acinetobacter sp. ADP1
Psychrobacter sp. PRwf-1
Psychrobacter cryohalolentis K5
Francisella tularensis SCHU S4
Legionella pneumophila str. Philadelphia 1
Coxiella burnetii str. RSA439
Rickettsiella viridis str. Ap-RA04
Vesicomyosocius okutanii HA
Ruthia magnifica str. Cm
Thiomicrospira crunogena XCL-2
Methylomonas methanica MC09
Methylococcus capsulatus str. Bath
Beggiatoa leptomitoformis str. D-401
Nitrosococcus oceani ATCC 19707
Nitrococcus mobilis Nb-231
Halorhodospira halophila SL1
Alkalilimnicola ehrlichii MLHE-1
Salinisphaera sp. LB1
Solimonas sp. K1W22B-7
Steroidobacter denitrificans str. DSM 18526
Xanthomonas axonopodis Xac29-1
Xanthomonas campestris str. ATCC 33913
Stenotrophomonas maltophilia R551-3
Xylella fastidiosa 9a5c
Cardiobacterium hominis str. NCTC10426
Dichelobacter nodosus VCS1703A
Chromobacterium violaceum ATCC 12472
Ralstonia solanacearum GMI1000
Acidithiobacillus caldus SM-1
Acidithiobacillus ferrooxidans ATCC 23270
Rhodospirillum rubrum ATCC 11170
Sinorhizobium meliloti 1021
Helicobacter pylori J99
Campylobacter jejuni 269.97
Geobacter sulfurreducens KN400
Myxococcus macrosporus str. HW-1
Candidatus Koribacter versatilis Ellin345
Acidobacterium capsulatum ATCC 51196
Betaproteobacteria
Acidithiobacillia
Alphaproteobacteria
Epsilonproteobacteria
Deltaproteobacteria
Acidobacteria
81
98
43
92
98
2
16
21
17, 25, 44
3
18
22
84
19, 23, 45
24
46
90
26
4
27, 47
28, 48
1
96
29a, 38a
5, 29b, 38b
7
84
6
8
39a, 49a
99
20
39b, 49b
99
9
10
11
40
30
12
90
13
95
41
31
14
32, 37
99
80
15, 33
34
50
35
36
99
98
51
42
52
95

FIG. S10. Phylogeny of the *Gammaproteobacteria*. A maximum likelihood phylogeny tree was produced using the PhyML algorithm (LG substitution model) based on the concatenated CLC alignments of 39 proteins within the *secE-rpoBC-str-S10-spc-alpha* operon cluster (supplementary table S2). Support for each node was evaluated by bootstrapping and nodes with a bootstrap value below 80% were collapsed. Support values for nodes are shown when these are below 100%. Branch lengths for the outgroup species was reduced by a factor of two (see scale value in parenthesis). Taxonomic orders are designated according to NCBI and evolutionary events are indicated in the tree. Evolutionary events are indicated above the branches. See supplementary table S6 for event details.

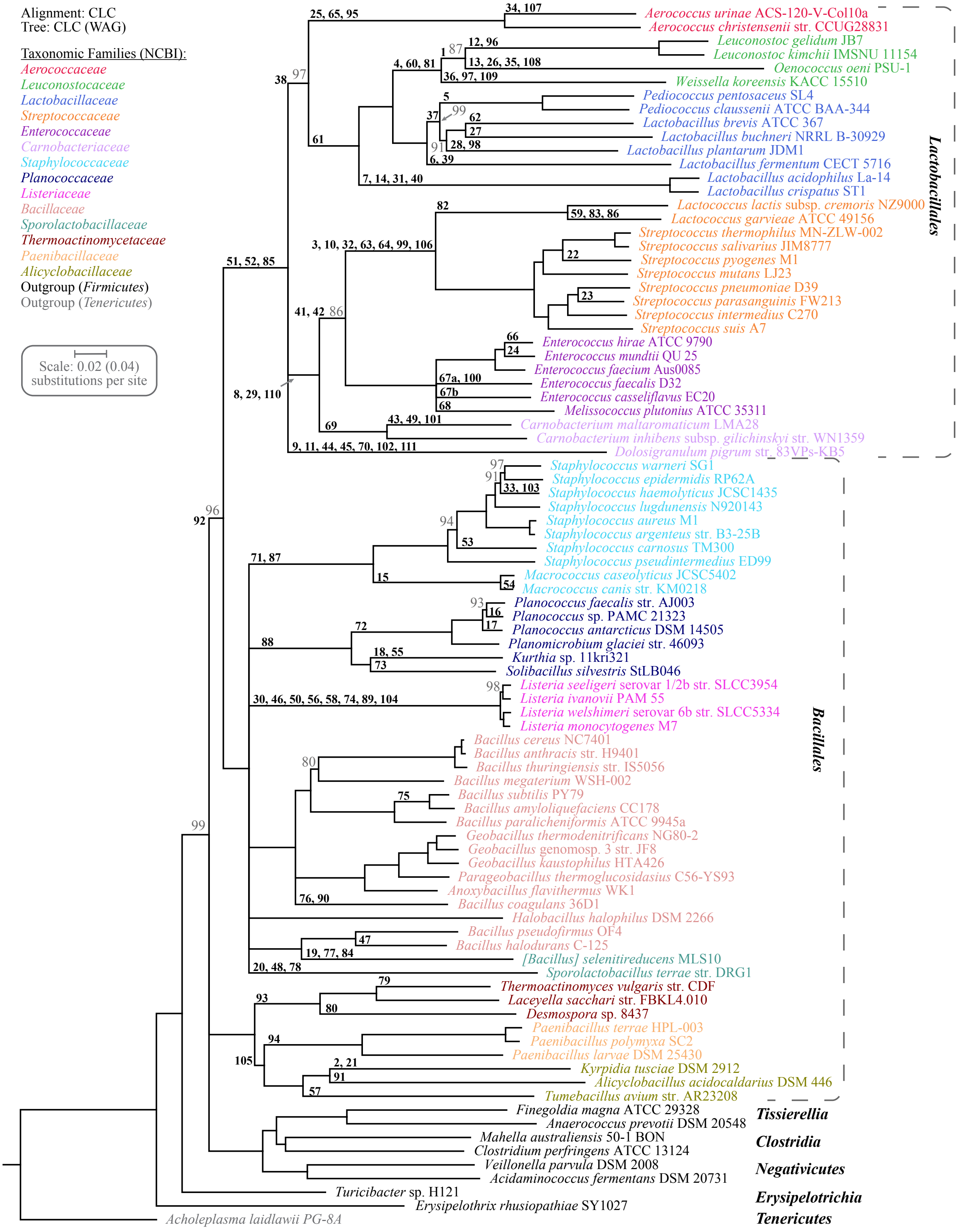

Alignment: CLC
Tree: CLC (WAG)
Taxonomic Families (NCBI):
Aerococcaceae
Leuconostocaceae
Lactobacillaceae
Streptococcaceae
Enterococcaceae
Carnobacteriaceae
Staphylococcaceae
Planococcaceae
Listeriaceae
Bacillaceae
Sporolactobacillaceae
Thermoactinomycetaceae
Paenibacillaceae
Alicyclobacillaceae
Outgroup (Firmicutes)
Outgroup (Tenericutes)
Scale: 0.02 (0.04) substitutions per site
Aerococcus urinae ACS-120-V-Col10a
Aerococcus christensenii str. CCUG28831
Leuconostoc gelidum JB7
Leuconostoc kimchii IMSNU 11154
Oenococcus oeni PSU-1
Weissella koreensis KACC 15510
Pediococcus pentosaceus SL4
Pediococcus claussenii ATCC BAA-344
Lactobacillus brevis ATCC 367
Lactobacillus buchneri NRRL B-30929
Lactobacillus plantarum JDM1
Lactobacillus fermentum CECT 5716
Lactobacillus acidophilus La-14
Lactobacillus crispatus ST1
Lactococcus lactis subsp. cremoris NZ9000
Lactococcus garvieae ATCC 49156
Streptococcus thermophilus MN-ZLW-002
Streptococcus salivarius JIM8777
Streptococcus pyogenes M1
Streptococcus mutans LJ23
Streptococcus pneumoniae D39
Streptococcus parasanguinis FW213
Streptococcus intermedius C270
Streptococcus suis A7
Enterococcus hirae ATCC 9790
Enterococcus mundtii QU 25
Enterococcus faecium Aus0085
Enterococcus faecalis D32
Enterococcus casseliflavus EC20
Melissococcus plutonius ATCC 35311
Carnobacterium maltaromaticum LMA28
Carnobacterium inhibens subsp. gilichinskyi str. WN1359
Dolosigranulum pigrum str. 83VPs-KB5
Lactobacillales
Staphylococcus warneri SG1
Staphylococcus epidermidis RP62A
Staphylococcus haemolyticus JCSC1435
Staphylococcus lugdunensis N920143
Staphylococcus aureus M1
Staphylococcus argenteus str. B3-25B
Staphylococcus carnosus TM300
Staphylococcus pseudintermedius ED99
Macrococcus caseolyticus JCSC5402
Macrococcus canis str. KM0218
Planococcus faecalis str. AJ003
Planococcus sp. PAMC 21323
Planococcus antarcticus DSM 14505
Planomicrobium glaciei str. 46093
Kurthia sp. 11kri321
Solibacillus silvestris StLB046
Listeria seeligeri serovar 1/2b str. SLCC3954
Listeria ivanovii PAM 55
Listeria welshimeri serovar 6b str. SLCC5334
Listeria monocytogenes M7
Bacillus cereus NC7401
Bacillus anthracis str. H9401
Bacillus thuringiensis str. IS5056
Bacillus megaterium WSH-002
Bacillus subtilis PY79
Bacillus amyloliquefaciens CC178
Bacillus paralicheniformis ATCC 9945a
Geobacillus thermodenitrificans NG80-2
Geobacillus genomosp. 3 str. JF8
Geobacillus kaustophilus HTA426
Parageobacillus thermoglucosidasius C56-YS93
Anoxybacillus flavithermus WK1
Bacillus coagulans 36D1
Halobacillus halophilus DSM 2266
Bacillus pseudofirmus OF4
Bacillus halodurans C-125
[Bacillus] selenitireducens MLS10
Sporolactobacillus terrae str. DRG1
Thermoactinomyces vulgaris str. CDF
Laceyella sacchari str. FBKL4.010
Desmospora sp. 8437
Paenibacillus terrae HPL-003
Paenibacillus polymyxa SC2
Paenibacillus larvae DSM 25430
Kyrpidia tusciae DSM 2912
Alicyclobacillus acidocaldarius DSM 446
Tumebacillus avium str. AR23208
Bacillales
Finegoldia magna ATCC 29328
Anaerococcus prevotii DSM 20548
Mahella australiensis 50-1 BON
Clostridium perfringens ATCC 13124
Veillonella parvula DSM 2008
Acidaminococcus fermentans DSM 20731
Turicibacter sp. H121
Erysipelothrix rhusiopathiae SY1027
Acholeplasma laidlawii PG-8A
Tissierellia
Clostridia
Negativicutes
Erysipelotrichia
Tenericutes
25, 65, 95
34, 107
38
97
1
87
12, 96
4, 60, 81
13, 26, 35, 108
36, 97, 109
61
5
37
99
62
27
91
28, 98
6, 39
7, 14, 31, 40
82
59, 83, 86
3, 10, 32, 63, 64, 99, 106
22
23
51, 52, 85
41, 42
86
66
24
8, 29, 110
67a, 100
67b
68
69
43, 49, 101
9, 11, 44, 45, 70, 102, 111
97
91
33, 103
96
92
94
53
71, 87
15
54
93
16
17
72
88
18, 55
73
98
30, 46, 50, 56, 58, 74, 89, 104
80
75
99
76, 90
47
19, 77, 84
20, 48, 78
79
93
80
94
105
2, 21
91
57

FIG. S11. Phylogeny of the *Bacilli*. A maximum likelihood phylogeny tree was produced using the CLC algorithm (WAG substitution model) based on the concatenated CLC alignments of 44 proteins within the *secE*-*rpoBC*-*str*-*S10*-*spc*-*alpha* operon cluster (supplementary table S3). Support for each node was evaluated by bootstrapping and nodes with a bootstrap value below 80% were collapsed. Support values for nodes are shown when these are below 100%. Branch lengths for the outgroup species was reduced by a factor of two (see scale value in parenthesis). Taxonomic orders and families are designated according to NCBI and evolutionary events are indicated in the tree. Evolutionary events are indicated above the branches. See supplementary table S6 for event details.

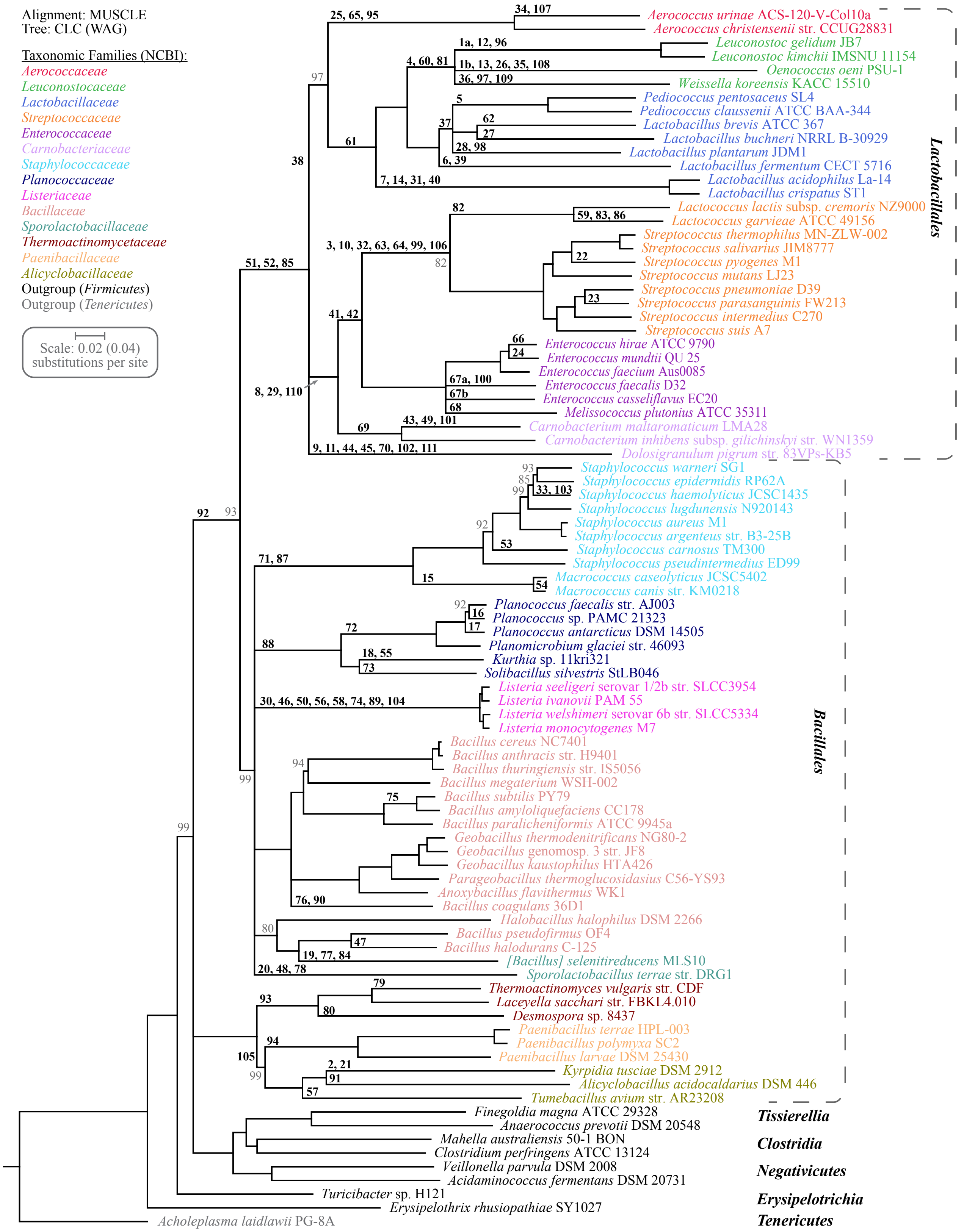

Alignment: MUSCLE
Tree: CLC (WAG)
Taxonomic Families (NCBI):
Aerococcaceae
Leuconostocaceae
Lactobacillaceae
Streptococcaceae
Enterococcaceae
Carnobacteriaceae
Staphylococcaceae
Planococcaceae
Listeriaceae
Bacillaceae
Sporolactobacillaceae
Thermoactinomycetaceae
Paenibacillaceae
Alicyclobacillaceae
Outgroup (Firmicutes)
Outgroup (Tenericutes)
Scale: 0.02 (0.04) substitutions per site
Aerococcus urinae ACS-120-V-Col10a
Aerococcus christensenii str. CCUG28831
Leuconostoc gelidum JB7
Leuconostoc kimchii IMSNU 11154
Oenococcus oeni PSU-1
Weissella koreensis KACC 15510
Pediococcus pentosaceus SL4
Pediococcus claussenii ATCC BAA-344
Lactobacillus brevis ATCC 367
Lactobacillus buchneri NRRL B-30929
Lactobacillus plantarum JDM1
Lactobacillus fermentum CECT 5716
Lactobacillus acidophilus La-14
Lactobacillus crispatus ST1
Lactococcus lactis subsp. cremoris NZ9000
Lactococcus garvieae ATCC 49156
Streptococcus thermophilus MN-ZLW-002
Streptococcus salivarius JIM8777
Streptococcus pyogenes M1
Streptococcus mutans LJ23
Streptococcus pneumoniae D39
Streptococcus parasanguinis FW213
Streptococcus intermedius C270
Streptococcus suis A7
Enterococcus hirae ATCC 9790
Enterococcus mundtii QU 25
Enterococcus faecium Aus0085
Enterococcus faecalis D32
Enterococcus casseliflavus EC20
Melissococcus plutonius ATCC 35311
Carnobacterium maltaromaticum LMA28
Carnobacterium inhibens subsp. gilichinskyi str. WN1359
Dolosigranulum pigrum str. 83VPs-KB5
Lactobacillales
Staphylococcus warneri SG1
Staphylococcus epidermidis RP62A
Staphylococcus haemolyticus JCSC1435
Staphylococcus lugdunensis N920143
Staphylococcus aureus M1
Staphylococcus argenteus str. B3-25B
Staphylococcus carnosus TM300
Staphylococcus pseudintermedius ED99
Macrococcus caseolyticus JCSC5402
Macrococcus canis str. KM0218
Planococcus faecalis str. AJ003
Planococcus sp. PAMC 21323
Planococcus antarcticus DSM 14505
Planomicrobium glaciei str. 46093
Kurthia sp. 11kri321
Solibacillus silvestris StLB046
Listeria seeligeri serovar 1/2b str. SLCC3954
Listeria ivanovii PAM 55
Listeria welshimeri serovar 6b str. SLCC5334
Listeria monocytogenes M7
Bacillus cereus NC7401
Bacillus anthracis str. H9401
Bacillus thuringiensis str. IS5056
Bacillus megaterium WSH-002
Bacillus subtilis PY79
Bacillus amyloliquefaciens CC178
Bacillus paralicheniformis ATCC 9945a
Geobacillus thermodenitrificans NG80-2
Geobacillus genomosp. 3 str. JF8
Geobacillus kaustophilus HTA426
Parageobacillus thermoglucosidasius C56-YS93
Anoxybacillus flavithermus WK1
Bacillus coagulans 36D1
Halobacillus halophilus DSM 2266
Bacillus pseudofirmus OF4
Bacillus halodurans C-125
[Bacillus] selenitireducens MLS10
Sporolactobacillus terrae str. DRG1
Thermoactinomyces vulgaris str. CDF
Laceyella sacchari str. FBKL4.010
Desmospora sp. 8437
Paenibacillus terrae HPL-003
Paenibacillus polymyxa SC2
Paenibacillus larvae DSM 25430
Kyrpidia tusciae DSM 2912
Alicyclobacillus acidocaldarius DSM 446
Tumebacillus avium str. AR23208
Bacillales
Finegoldia magna ATCC 29328
Anaerococcus prevotii DSM 20548
Tissierellia
Mahella australiensis 50-1 BON
Clostridium perfringens ATCC 13124
Clostridia
Veillonella parvula DSM 2008
Acidaminococcus fermentans DSM 20731
Negativicutes
Turicibacter sp. H121
Erysipelothrix rhusiopathiae SY1027
Erysipelotrichia
Acholeplasma laidlawii PG-8A
Tenericutes

FIG. S12. Phylogeny of the *Bacilli*. A maximum likelihood phylogeny tree was produced using the CLC algorithm (WAG substitution model) based on the concatenated MUSCLE alignments of 44 proteins within the *secE-rpoBC-str-S10-spc-alpha* operon cluster (supplementary table S3). Support for each node was evaluated by bootstrapping and nodes with a bootstrap value below 80% were collapsed. Support values for nodes are shown when these are below 100%. Branch lengths for the outgroup species was reduced by a factor of two (see scale value in parenthesis). Taxonomic orders and families are designated according to NCBI and evolutionary events are indicated in the tree. Evolutionary events are indicated above the branches. See supplementary table S6 for event details.

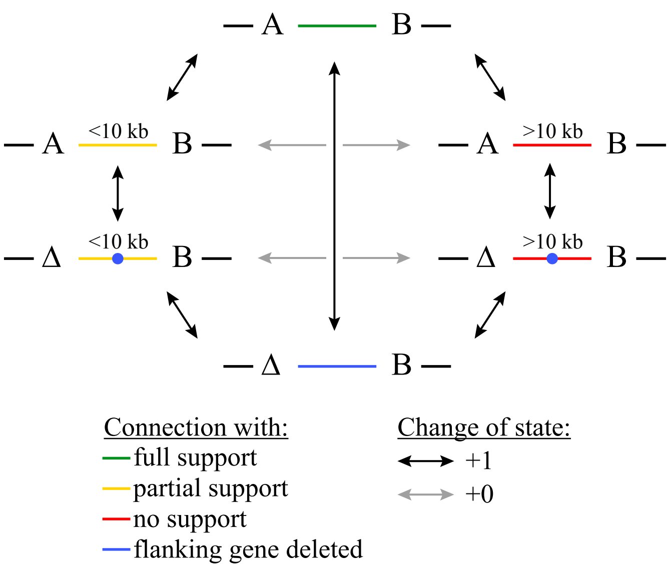

FIG. S13. Definition of potential state changes for a gene pair (A and B) used in the reconstruction of the ancestral operon cluster.

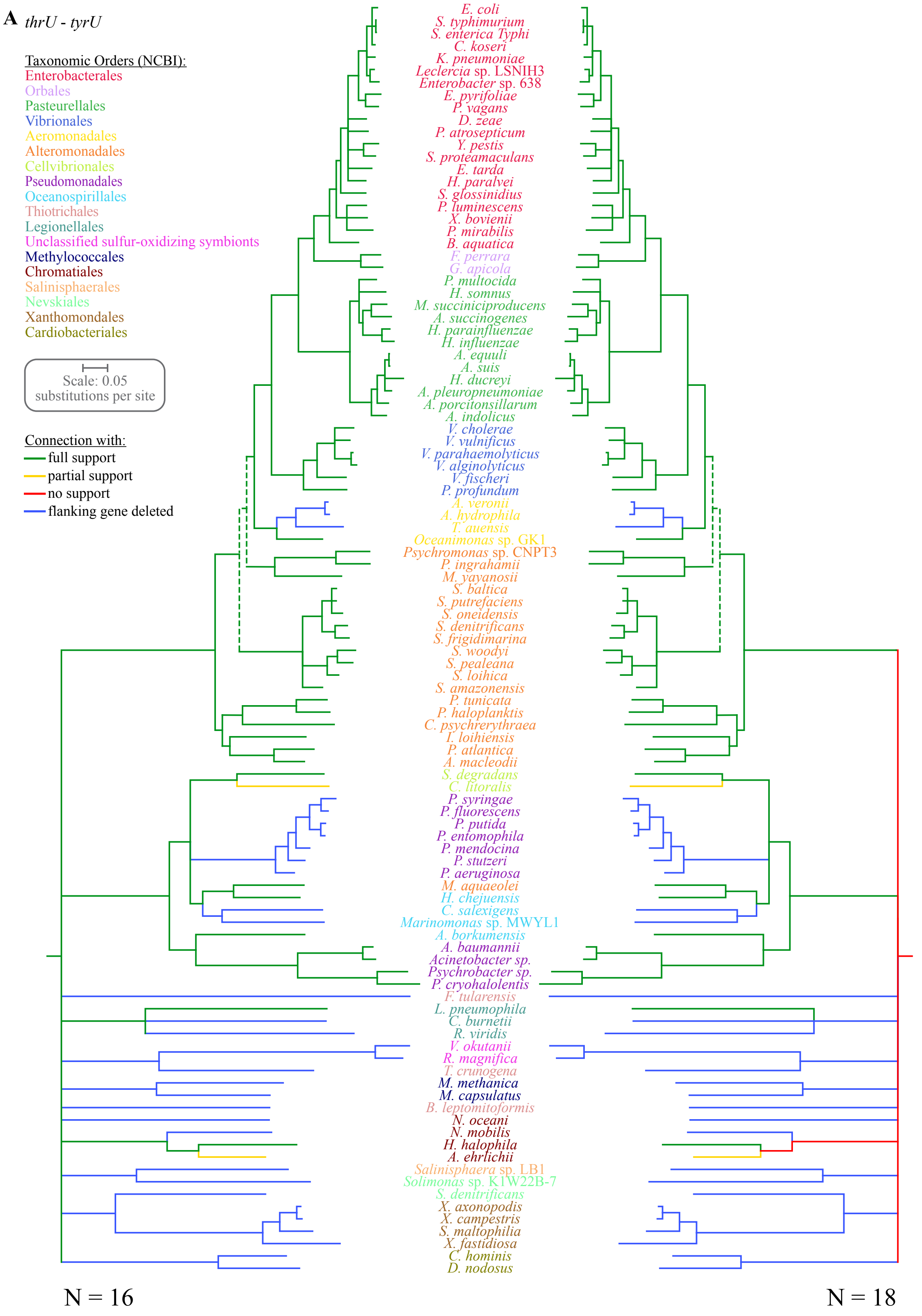
A thrU - tyrU
Taxonomic Orders (NCBI):
Enterobacterales
Orbales
Pasteurellales
Vibrionales
Aeromonadales
Alteromonadales
Cellvibrionales
Pseudomonadales
Oceanospirillales
Thiotrichales
Legionellales
Unclassified sulfur-oxidizing symbionts
Methylococcales
Chromatiales
Salinisphaerales
Nevskiales
Xanthomondales
Cardiobacteriales
Scale: 0.05
substitutions per site
Connection with:
full support
partial support
no support
flanking gene deleted
E. coli
S. typhimurium
S. enterica Typhi
C. koseri
K. pneumoniae
Leclercia sp. LSNIH3
Enterobacter sp. 638
E. pyrifoliae
P. vagans
D. zeae
P. atrosepticum
Y. pestis
S. proteamaculans
E. tarda
H. paralvei
S. glossinidius
P. luminescens
X. bovienii
P. mirabilis
B. aquatica
F. perrara
G. apicola
P. multocida
H. somnus
M. succiniciproducens
A. succinogenes
H. parainfluenzae
H. influenzae
A. equuli
A. suis
H. ducreyi
A. pleuropneumoniae
A. porcitonsillarum
A. indolicus
V. cholerae
V. vulnificus
V. parahaemolyticus
V. alginolyticus
V. fischeri
P. profundum
A. veronii
A. hydrophila
T. auensis
Oceanimonas sp. GK1
Psychromonas sp. CNPT3
P. ingrahamii
M. yayanosii
S. baltica
S. putrefaciens
S. oneidensis
S. denitrificans
S. frigidimarina
S. woodyi
S. pealeana
S. loihica
S. amazonensis
P. tunicata
P. haloplanktis
C. psychrerythraea
I. loihiensis
P. atlantica
A. macleodii
S. degradans
C. litoralis
P. syringae
P. fluorescens
P. putida
P. entomophila
P. mendocina
P. stutzeri
P. aeruginosa
M. aquaeolei
H. chejuensis
C. salexigens
Marinomonas sp. MWYL1
A. borkumensis
A. baumannii
Acinetobacter sp.
Psychrobacter sp.
P. cryohalolentis
F. tularensis
L. pneumophila
C. burnetii
R. viridis
V. okutanii
R. magnifica
T. crunogena
M. methanica
M. capsulatus
B. leptomitoformis
N. oceani
N. mobilis
H. halophila
A. ehrlichii
Salinisphaera sp. LB1
Solimonas sp. K1W22B-7
S. denitrificans
X. axonopodis
X. campestris
S. maltophilia
X. fastidiosa
C. hominis
D. nodosus
N = 16
N = 18

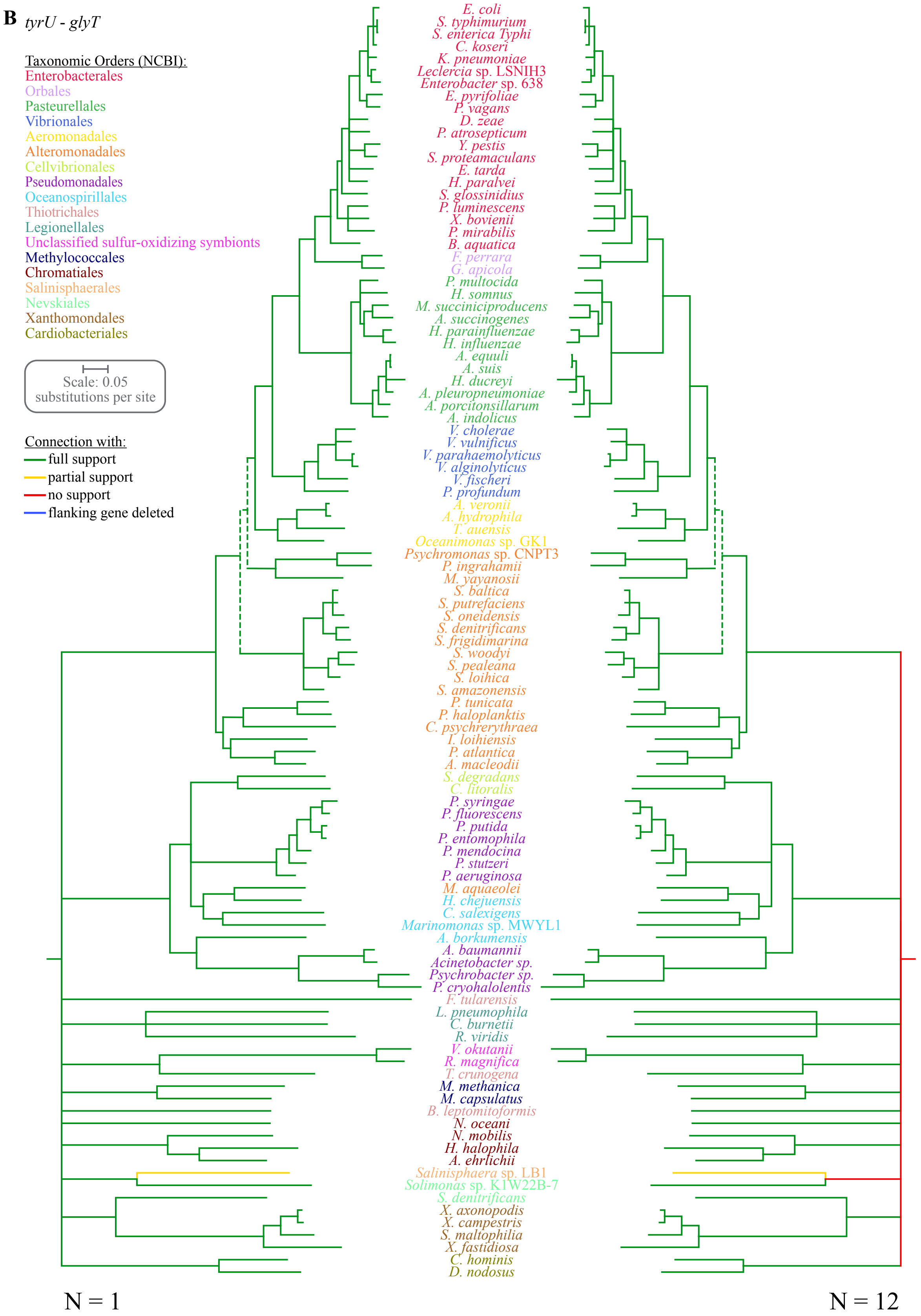
B tyrU - glyT
Taxonomic Orders (NCBI):
Enterobacterales
Orbales
Pasteurellales
Vibrionales
Aeromonadales
Alteromonadales
Cellvibrionales
Pseudomonadales
Oceanospirillales
Thiotrichales
Legionellales
Unclassified sulfur-oxidizing symbionts
Methylococcales
Chromatiales
Salinisphaerales
Nevskiales
Xanthomonadales
Cardiobacteriales
Scale: 0.05
substitutions per site
Connection with:
full support
partial support
no support
flanking gene deleted
E. coli
S. typhimurium
S. enterica Typhi
C. koseri
K. pneumoniae
Leclercia sp. LSNIH3
Enterobacter sp. 638
E. pyrifoliae
P. vagans
D. zeae
P. atrosepticum
Y. pestis
S. proteamaculans
E. tarda
H. paralvei
S. glossinidius
P. luminescens
X. bovienii
P. mirabilis
B. aquatica
F. perrara
G. apicola
P. multocida
H. somnus
M. succiniciproducens
A. succinogenes
H. parainfluenzae
H. influenzae
A. equuli
A. suis
H. ducreyi
A. pleuropneumoniae
A. porcitonsillarum
A. indolicus
V. cholerae
V. vulnificus
V. parahaemolyticus
V. alginolyticus
V. fischeri
P. profundum
A. veronii
A. hydrophila
T. auensis
Oceanimonas sp. GK1
Psychromonas sp. CNPT3
P. ingrahamii
M. yayanosii
S. baltica
S. putrefaciens
S. oneidensis
S. denitrificans
S. frigidimarina
S. woodyi
S. pealeana
S. loihica
S. amazonensis
P. tunicata
P. haloplanktis
C. psychrerythraea
I. loihiensis
P. atlantica
A. macleodii
S. degradans
C. litoralis
P. syringae
P. fluorescens
P. putida
P. entomophila
P. mendocina
P. stutzeri
P. aeruginosa
M. aquaeoli
H. chejuensis
C. salexigens
Marinomonas sp. MWYL1
A. borkumensis
A. baumannii
Acinetobacter sp.
Psychrobacter sp.
P. cryohalolentis
F. tularensis
L. pneumophila
C. burnetii
R. viridis
V. okutanii
R. magnifica
T. crunogena
M. methanica
M. capsulatus
B. leptomitoformis
N. oceani
N. mobilis
H. halophila
A. ehrlichii
Salinisphaera sp. LB1
Solimonas sp. K1W22B-7
S. denitrificans
X. axonopodis
X. campestris
S. maltophilia
X. fastidiosa
C. hominis
D. nodosus
N = 1
N = 12

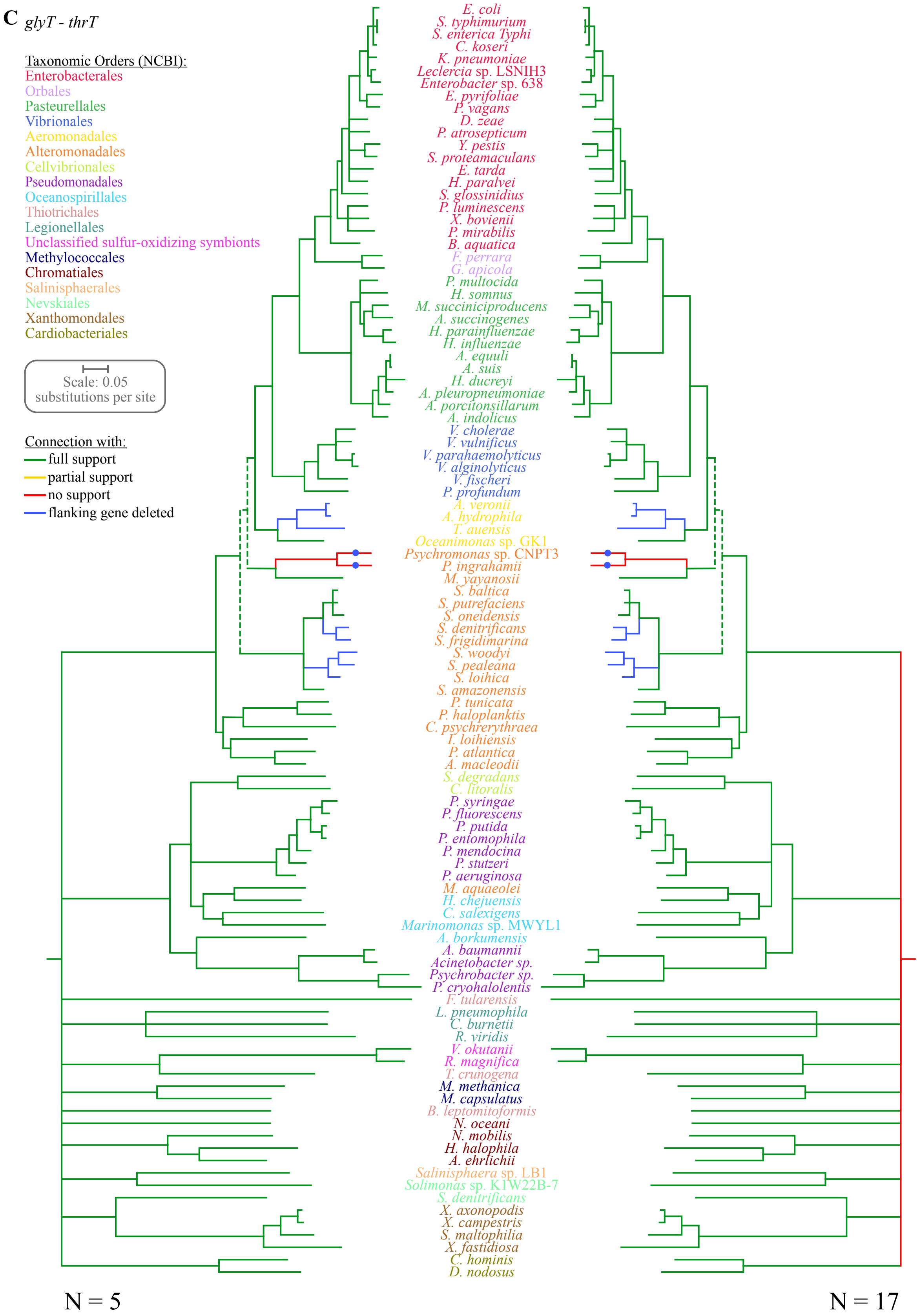
C glyT - thrT
Taxonomic Orders (NCBI):
Enterobacterales
Orbales
Pasteurellales
Vibrionales
Aeromonadales
Alteromonadales
Cellvibrionales
Pseudomonadales
Oceanospirillales
Thiotrichales
Legionellales
Unclassified sulfur-oxidizing symbionts
Methylococcales
Chromatiales
Salinisphaerales
Nevskiales
Xanthomondales
Cardiobacteriales
Scale: 0.05 substitutions per site
Connection with:
full support
partial support
no support
flanking gene deleted
E. coli
S. typhimurium
S. enterica Typhi
C. koseri
K. pneumoniae
Leclercia sp. LSNIH3
Enterobacter sp. 638
E. pyrifoliae
P. vagans
D. zeae
P. atrosepticum
Y. pestis
S. proteamaculans
E. tarda
H. paralvei
S. glossinidius
P. luminescens
X. bovienii
P. mirabilis
B. aquatica
F. perrara
G. apicola
P. multocida
H. somnus
M. succiniciproducens
A. succinogenes
H. parainfluenzae
H. influenzae
A. equuli
A. suis
H. ducreyi
A. pleuropneumoniae
A. porcitonsillarum
A. indolicus
V. cholerae
V. vulnificus
V. parahaemolyticus
V. alginolyticus
V. fischeri
P. profundum
A. veronii
A. hydrophila
T. auensis
Oceanimonas sp. GK1
Psychromonas sp. CNPT3
P. ingrahamii
M. yayanosii
S. baltica
S. putrefaciens
S. oneidensis
S. denitrificans
S. frigidimarina
S. woodyi
S. pealeana
S. loihica
S. amazonensis
P. tunicata
P. haloplanktis
C. psychrerythraea
I. loihiensis
P. atlantica
A. macleodii
S. degradans
C. litoralis
P. syringae
P. fluorescens
P. putida
P. entomophila
P. mendocina
P. stutzeri
P. aeruginosa
M. aquaeolei
H. chejuensis
C. salexigens
Marinomonas sp. MWYL1
A. borkumensis
A. baumannii
Acinetobacter sp.
Psychrobacter sp.
P. cryohalolentis
F. tularensis
L. pneumophila
C. burnetii
R. viridis
V. okutanii
R. magnifica
T. crunogena
M. methanica
M. capsulatus
B. leptomitoformis
N. oceani
N. mobilis
H. halophila
A. ehrlichii
Salinisphaera sp. LB1
Solimonas sp. K1W22B-7
S. denitrificans
X. axonopodis
X. campestris
S. maltophilia
X. fastidiosa
C. hominis
D. nodosus
N = 5
N = 17

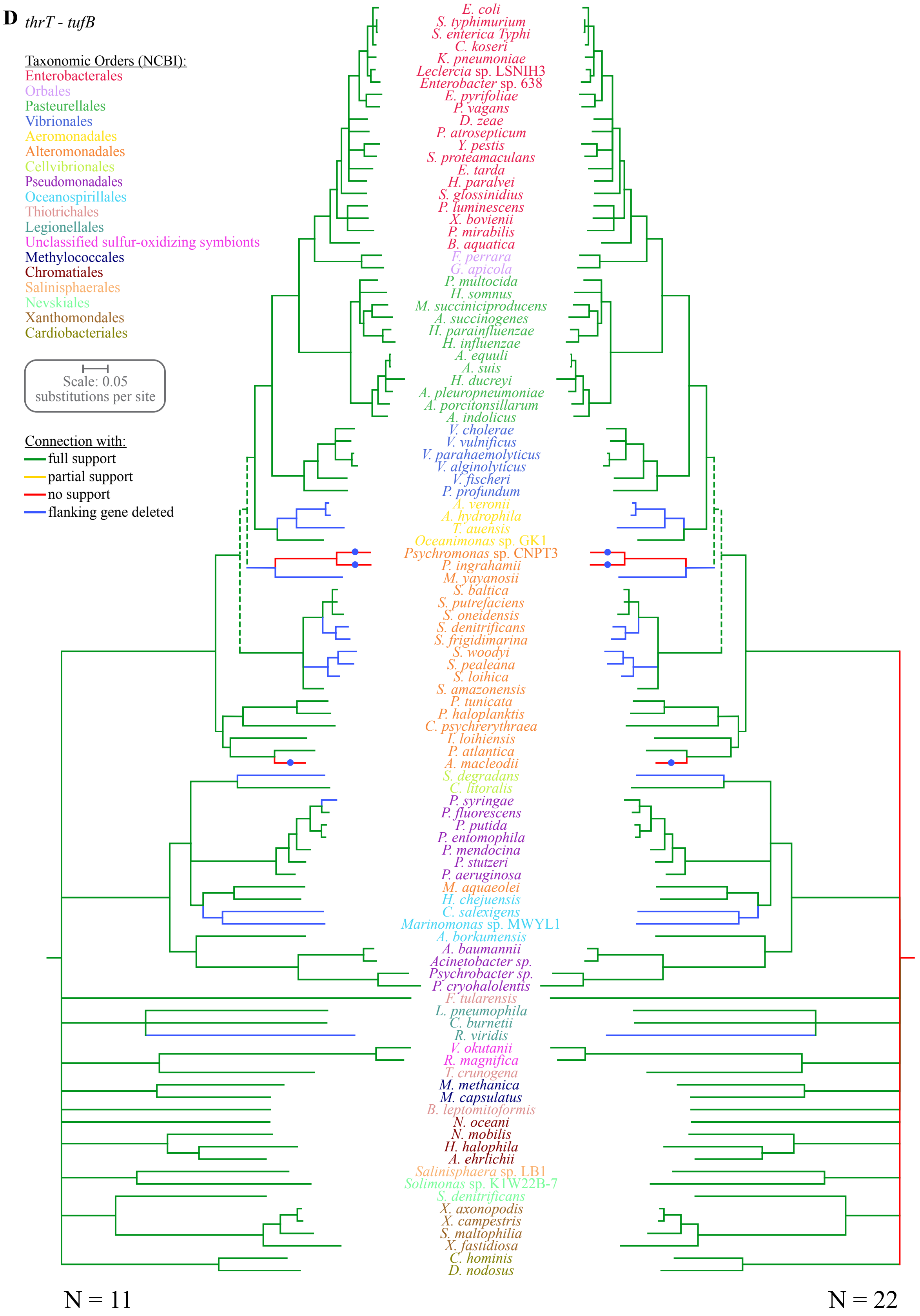
D thrT - tufB
Taxonomic Orders (NCBI):
Enterobacterales
Orbales
Pasteurellales
Vibrionales
Aeromonadales
Alteromonadales
Cellvibrionales
Pseudomonadales
Oceanospirillales
Thiotrichales
Legionellales
Unclassified sulfur-oxidizing symbionts
Methylococcales
Chromatiales
Salinisphaerales
Nevskiales
Xanthomondales
Cardiobacteriales
Scale: 0.05
substitutions per site
Connection with:
full support
partial support
no support
flanking gene deleted
E. coli
S. typhimurium
S. enterica Typhi
C. koseri
K. pneumoniae
Leclercia sp. LSNIH3
Enterobacter sp. 638
E. pyrifoliae
P. vagans
D. zeae
P. atrosepticum
Y. pestis
S. proteamaculans
E. tarda
H. paralvei
S. glossinidius
P. luminescens
X. bovienii
P. mirabilis
B. aquatica
F. perrara
G. apicola
P. multocida
H. somnus
M. succiniciproducens
A. succinogenes
H. parainfluenzae
H. influenzae
A. equuli
A. suis
H. ducreyi
A. pleuropneumoniae
A. porcitonsillarum
A. indolicus
V. cholerae
V. vulnificus
V. parahaemolyticus
V. alginolyticus
V. fischeri
P. profundum
A. veronii
A. hydrophila
T. auensis
Oceanimonas sp. GK1
Psychromonas sp. CNPT3
P. ingrahamii
M. yayanosii
S. baltica
S. putrefaciens
S. oneidensis
S. denitrificans
S. frigidimarina
S. woodyi
S. pealeana
S. loihica
S. amazonensis
P. tunicata
P. haloplanktis
C. psychrerythraea
I. loihiensis
P. atlantica
A. macleodii
S. degradans
C. litoralis
P. syringae
P. fluorescens
P. putida
P. entomophila
P. mendocina
P. stutzeri
P. aeruginosa
M. aquaeolei
H. chejuensis
C. salexigens
Marinomonas sp. MWYL1
A. borkumensis
A. baumannii
Acinetobacter sp.
Psychrobacter sp.
P. cryohalolentis
F. tularensis
L. pneumophila
C. burnetii
R. viridis
V. okutanii
R. magnifica
T. crunogena
M. methanica
M. capsulatus
B. leptomitoformis
N. oceani
N. mobilis
H. halophila
A. ehrlichii
Salinisphaera sp. LB1
Solimonas sp. K1W22B-7
S. denitrificans
X. axonopodis
X. campestris
S. maltophilia
X. fastidiosa
C. hominis
D. nodosus
N = 11
N = 22

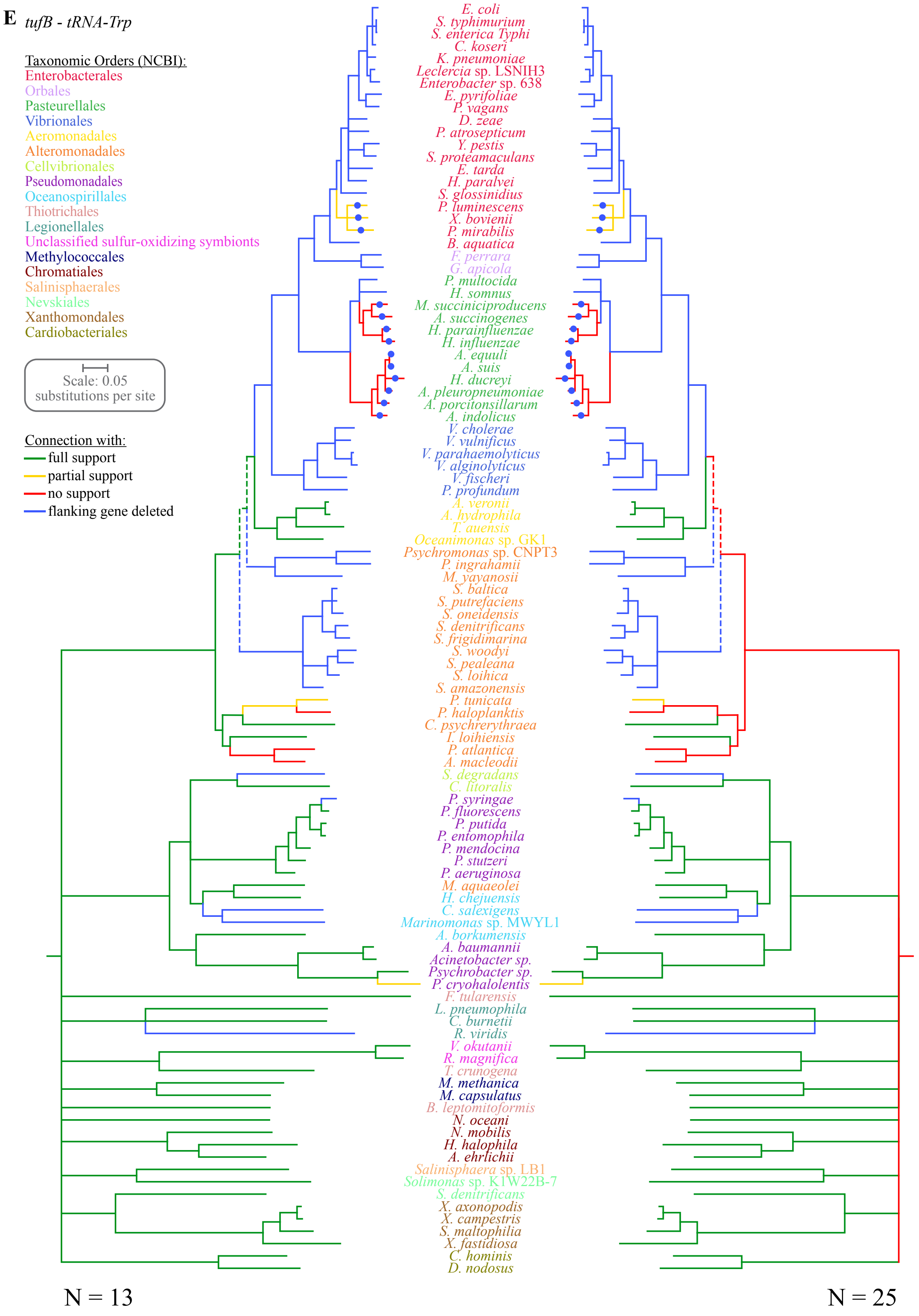

E
tufB - tRNA-Trp
Taxonomic Orders (NCBI):
Enterobacterales
Orbales
Pasteurellales
Vibrionales
Aeromonadales
Alteromonadales
Cellvibrionales
Pseudomonadales
Oceanospirillales
Thiotrichales
Legionellales
Unclassified sulfur-oxidizing symbionts
Methylococcales
Chromatiales
Salinisphaerales
Nevskiales
Xanthomondales
Cardiobacteriales
Scale: 0.05
substitutions per site
Connection with:
full support
partial support
no support
flanking gene deleted
E. coli
S. typhimurium
S. enterica Typhi
C. koseri
K. pneumoniae
Leclercia sp. LSNIH3
Enterobacter sp. 638
E. pyrifoliae
P. vagans
D. zeae
P. atrosepticum
Y. pestis
S. proteamaculans
E. tarda
H. paralvei
S. glossinidius
P. luminescens
X. bovienii
P. mirabilis
B. aquatica
F. perrara
G. apicola
P. multocida
H. somnus
M. succiniciproducens
A. succinogenes
H. parainfluenzae
H. influenzae
A. equuli
A. suis
H. ducreyi
A. pleuropneumoniae
A. porcitonsillarum
A. indolicus
V. cholerae
V. vulnificus
V. parahaemolyticus
V. alginolyticus
V. fischeri
P. profundum
A. veronii
A. hydrophila
T. auensis
Oceanimonas sp. GK1
Psychromonas sp. CNPT3
P. ingrahamii
M. yayanosii
S. baltica
S. putrefaciens
S. oneidensis
S. denitrificans
S. frigidimarina
S. woodyi
S. pealeana
S. loihica
S. amazonensis
P. tunicata
P. haloplanktis
C. psychrerythraea
I. loihiensis
P. atlantica
A. macleodii
S. degradans
C. litoralis
P. syringae
P. fluorescens
P. putida
P. entomophila
P. mendocina
P. stutzeri
P. aeruginosa
M. aquaeolei
H. chejuensis
C. salexigens
Marinomonas sp. MWYL1
A. borkumensis
A. baumannii
Acinetobacter sp.
Psychrobacter sp.
P. cryohalolentis
F. tularensis
L. pneumophila
C. burnetii
R. viridis
V. okutanii
R. magnifica
T. crunogena
M. methanica
M. capsulatus
B. leptomitoformis
N. oceani
N. mobilis
H. halophila
A. ehrlichii
Salinisphaera sp. LB1
Solimonas sp. K1W22B-7
S. denitrificans
X. axonopodis
X. campestris
S. maltophilia
X. fastidiosa
C. hominis
D. nodosus
N = 13
N = 25

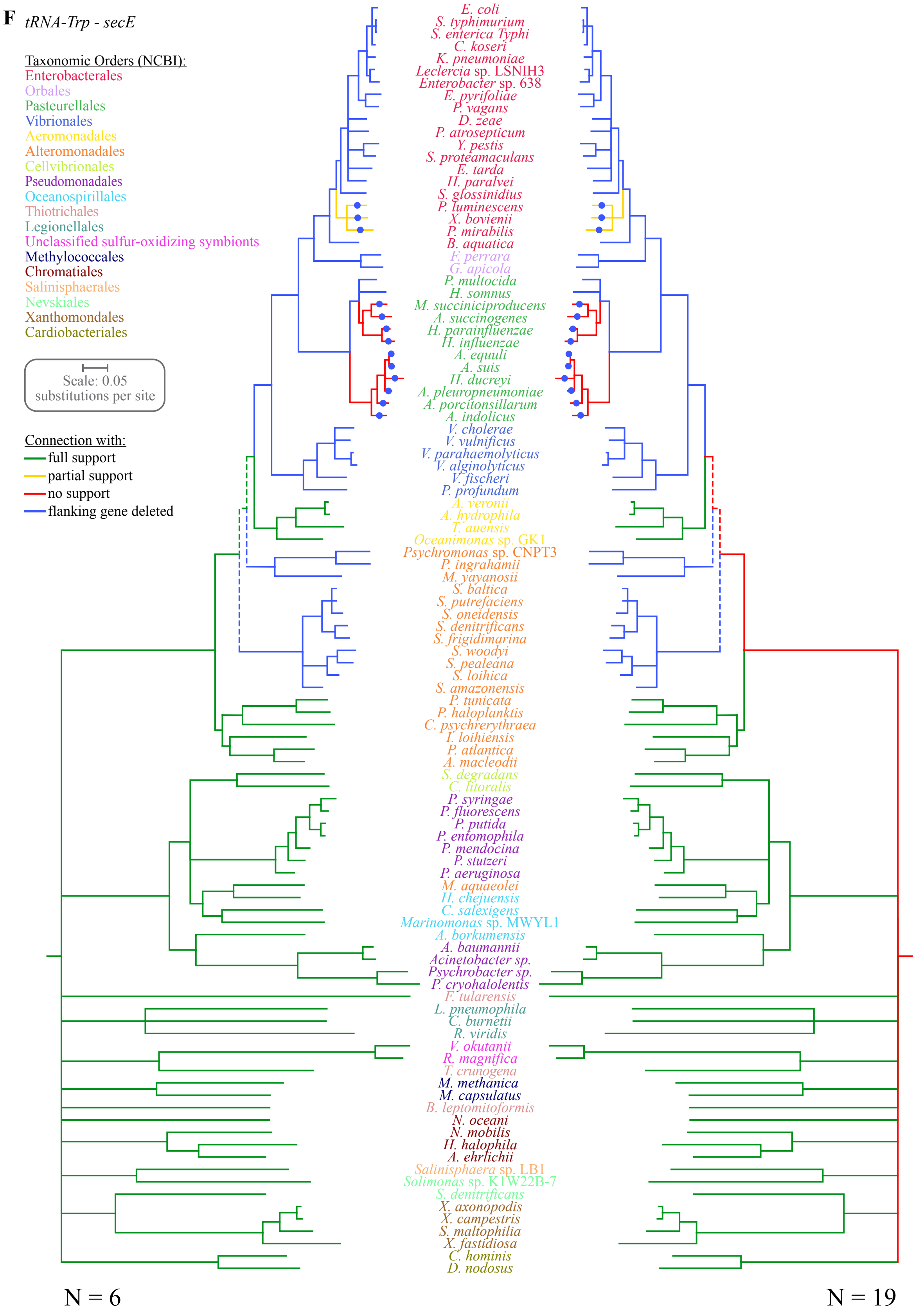
F
tRNA-Trp - secE
Taxonomic Orders (NCBI):
Enterobacterales
Orbales
Pasteurellales
Vibrionales
Aeromonadales
Alteromonadales
Cellvibrionales
Pseudomonadales
Oceanospirillales
Thiotrichales
Legionellales
Unclassified sulfur-oxidizing symbionts
Methylococcales
Chromatiales
Salinisphaerales
Nevskiales
Xanthomondales
Cardiobacteriales
Scale: 0.05
substitutions per site
Connection with:
full support
partial support
no support
flanking gene deleted
E. coli
S. typhimurium
S. enterica Typhi
C. koseri
K. pneumoniae
Leclercia sp. LSNIH3
Enterobacter sp. 638
E. pyrifoliae
P. vagans
D. zeae
P. atrosepticum
Y. pestis
S. proteamaculans
E. tarda
H. paralvei
S. glossinidius
P. luminescens
X. bovienii
P. mirabilis
B. aquatica
F. perrara
G. apicola
P. multocida
H. somnus
M. succiniciproducens
A. succinogenes
H. parainfluenzae
H. influenzae
A. equuli
A. suis
H. ducreyi
A. pleuropneumoniae
A. porcitonsillarum
A. indolicus
V. cholerae
V. vulnificus
V. parahaemolyticus
V. alginolyticus
V. fischeri
P. profundum
A. veronii
A. hydrophila
T. auensis
Oceanimonas sp. GK1
Psychromonas sp. CNPT3
P. ingrahamii
M. yayanosii
S. baltica
S. putrefaciens
S. oneidensis
S. denitrificans
S. frigidimarina
S. woodyi
S. pealeana
S. loihica
S. amazonensis
P. tunicata
P. haloplanktis
C. psychrerythraea
I. loihiensis
P. atlantica
A. macleodii
S. degradans
C. litoralis
P. syringae
P. fluorescens
P. putida
P. entomophila
P. mendocina
P. stutzeri
P. aeruginosa
M. aquaeolei
H. chejuensis
C. salexigens
Marinomonas sp. MWYL1
A. borkumensis
A. baumannii
Acinetobacter sp.
Psychrobacter sp.
P. cryohalolentis
F. tularensis
L. pneumophila
C. burnetii
R. viridis
V. okutanii
R. magnifica
T. crunogena
M. methanica
M. capsulatus
B. leptomitoformis
N. oceani
N. mobilis
H. halophila
A. ehrlichii
Salinisphaera sp. LB1
Solimonas sp. K1W22B-7
S. denitrificans
X. axonopodis
X. campestris
S. maltophilia
X. fastidiosa
C. hominis
D. nodosus
N = 6
N = 19

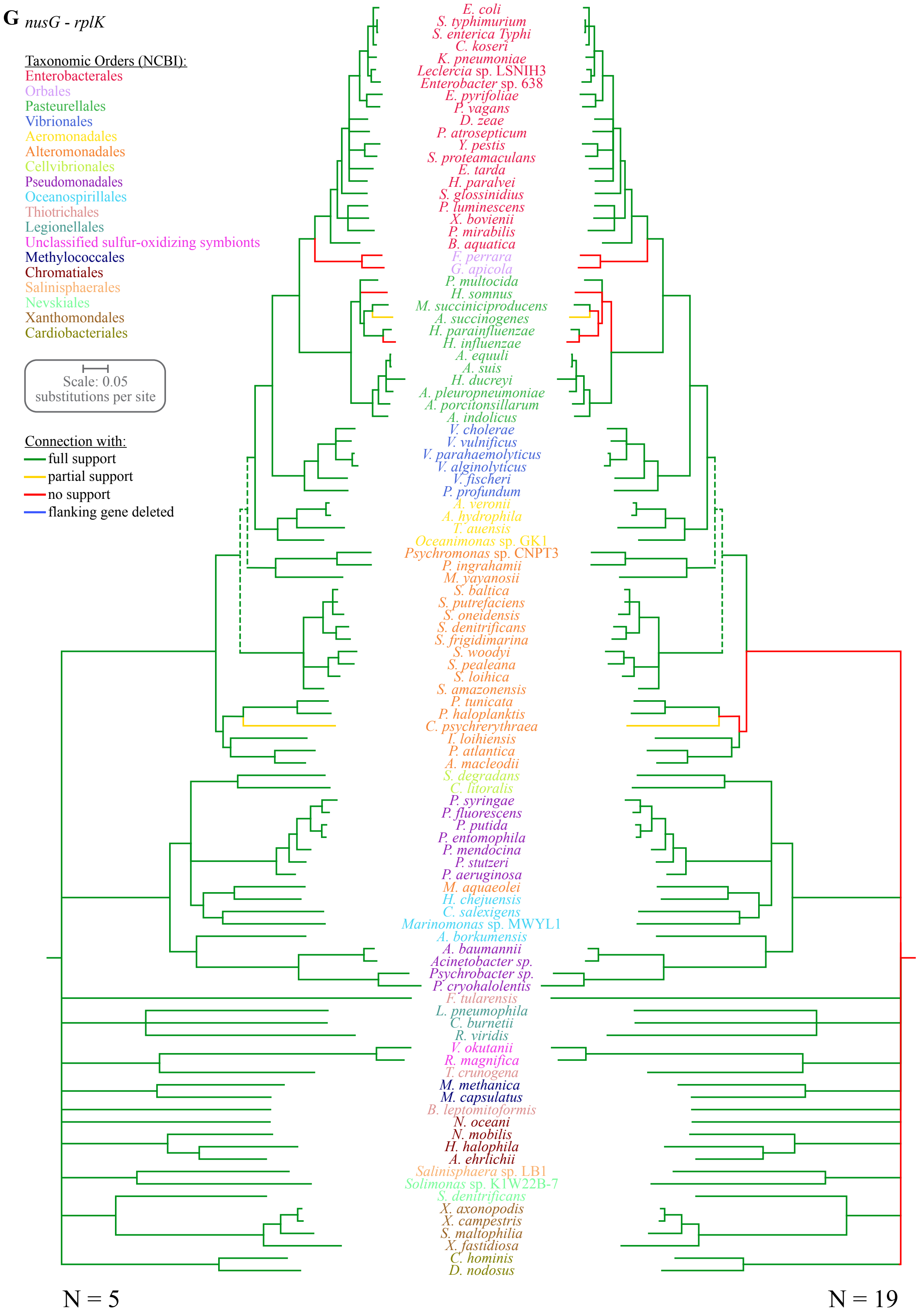
G nusG - rplK
Taxonomic Orders (NCBI):
Enterobacterales
Orbales
Pasteurellales
Vibrionales
Aeromonadales
Alteromonadales
Cellvibrionales
Pseudomonadales
Oceanospirillales
Thiotrichales
Legionellales
Unclassified sulfur-oxidizing symbionts
Methylococcales
Chromatiales
Salinisphaerales
Nevskiales
Xanthomondales
Cardiobacteriales
Scale: 0.05
substitutions per site
Connection with:
full support
partial support
no support
flanking gene deleted
E. coli
S. typhimurium
S. enterica Typhi
C. koseri
K. pneumoniae
Leclercia sp. LSNIH3
Enterobacter sp. 638
E. pyrifoliae
P. vagans
D. zeae
P. atrosepticum
Y. pestis
S. proteamaculans
E. tarda
H. paralvei
S. glossinidius
P. luminescens
X. bovienii
P. mirabilis
B. aquatica
F. perrara
G. apicola
P. multocida
H. somnus
M. succiniciproducens
A. succinogenes
H. parainfluenzae
H. influenzae
A. equuli
A. suis
H. ducreyi
A. pleuropneumoniae
A. porcitonsillarum
A. indolicus
V. cholerae
V. vulnificus
V. parahaemolyticus
V. alginolyticus
V. fischeri
P. profundum
A. veronii
A. hydrophila
T. auensis
Oceanimonas sp. GK1
Psychromonas sp. CNPT3
P. ingrahamii
M. yayanosii
S. baltica
S. putrefaciens
S. oneidensis
S. denitrificans
S. frigidimarina
S. woodyi
S. pealeana
S. loihica
S. amazonensis
P. tunicata
P. haloplanktis
C. psychrerythraea
I. loihiensis
P. atlantica
A. macleodii
S. degradans
C. litoralis
P. syringae
P. fluorescens
P. putida
P. entomophila
P. mendocina
P. stutzeri
P. aeruginosa
M. aquaeolei
H. chejuensis
C. salexigens
Marinomonas sp. MWYL1
A. borkumensis
A. baumannii
Acinetobacter sp.
Psychrobacter sp.
P. cryohalolentis
F. tularensis
L. pneumophila
C. burnetii
R. viridis
V. okutanii
R. magnifica
T. crunogena
M. methanica
M. capsulatus
B. leptomitoformis
N. oceani
N. mobilis
H. halophila
A. ehrlichii
Salinisphaera sp. LB1
Solimonas sp. K1W22B-7
S. denitrificans
X. axonopodis
X. campestris
S. maltophilia
X. fastidiosa
C. hominis
D. nodosus
N = 5
N = 19

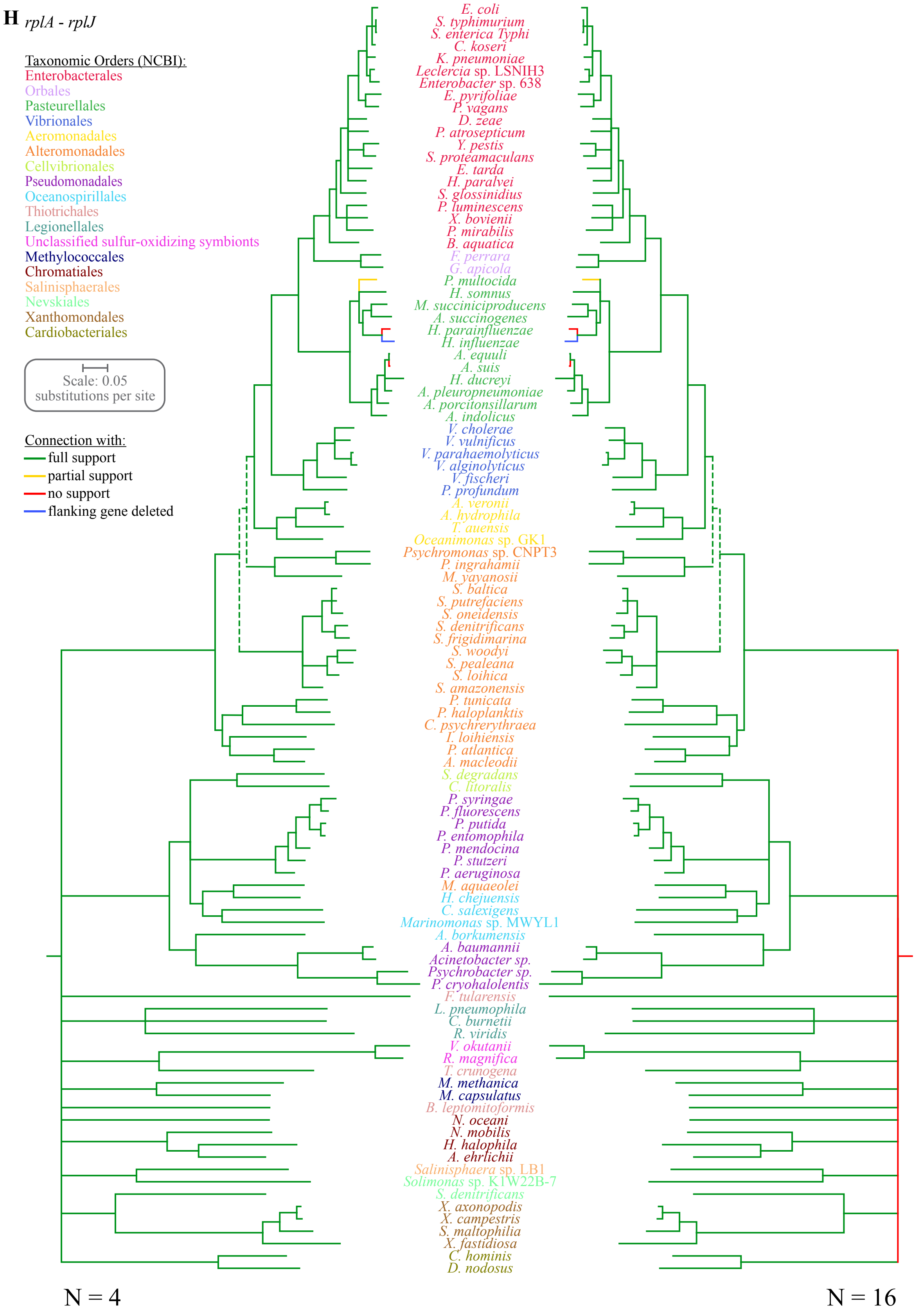
H rplA - rplJ
Taxonomic Orders (NCBI):
Enterobacterales
Orbales
Pasteurellales
Vibrionales
Aeromonadales
Alteromonadales
Cellvibrionales
Pseudomonadales
Oceanospirillales
Thiotrichales
Legionellales
Unclassified sulfur-oxidizing symbionts
Methylococcales
Chromatiales
Salinisphaerales
Nevskiales
Xanthomondales
Cardiobacteriales
Scale: 0.05
substitutions per site
Connection with:
full support
partial support
no support
flanking gene deleted
E. coli
S. typhimurium
S. enterica Typhi
C. koseri
K. pneumoniae
Leclercia sp. LSNIH3
Enterobacter sp. 638
E. pyrifoliae
P. vagans
D. zeae
P. atrosepticum
Y. pestis
S. proteamaculans
E. tarda
H. paralvei
S. glossinidius
P. luminescens
X. bovienii
P. mirabilis
B. aquatica
F. perrara
G. apicola
P. multocida
H. somnus
M. succiniciproducens
A. succinogenes
H. parainfluenzae
H. influenzae
A. equuli
A. suis
H. ducreyi
A. pleuropneumoniae
A. porcitonsillarum
A. indolicus
V. cholerae
V. vulnificus
V. parahaemolyticus
V. alginolyticus
V. fischeri
P. profundum
A. veronii
A. hydrophila
T. auensis
Oceanimonas sp. GK1
Psychromonas sp. CNPT3
P. ingrahamii
M. yayanosii
S. baltica
S. putrefaciens
S. oneidensis
S. denitrificans
S. frigidimarina
S. woodyi
S. pealeana
S. loihica
S. amazonensis
P. tunicata
P. haloplanktis
C. psychrerythraea
I. loihiensis
P. atlantica
A. macleodii
S. degradans
C. litoralis
P. syringae
P. fluorescens
P. putida
P. entomophila
P. mendocina
P. stutzeri
P. aeruginosa
M. aquaeolei
H. chejuensis
C. salexigens
Marinomonas sp. MWYL1
A. borkumensis
A. baumannii
Acinetobacter sp.
Psychrobacter sp.
P. cryohalolentis
F. tularensis
L. pneumophila
C. burnetii
R. viridis
V. okutanii
R. magnifica
T. crunogena
M. methanica
M. capsulatus
B. leptomitoformis
N. oceani
N. mobilis
H. halophila
A. ehrlichii
Salinisphaera sp. LB1
Solimonas sp. K1W22B-7
S. denitrificans
X. axonopodis
X. campestris
S. maltophilia
X. fastidiosa
C. hominis
D. nodosus
N = 4
N = 16

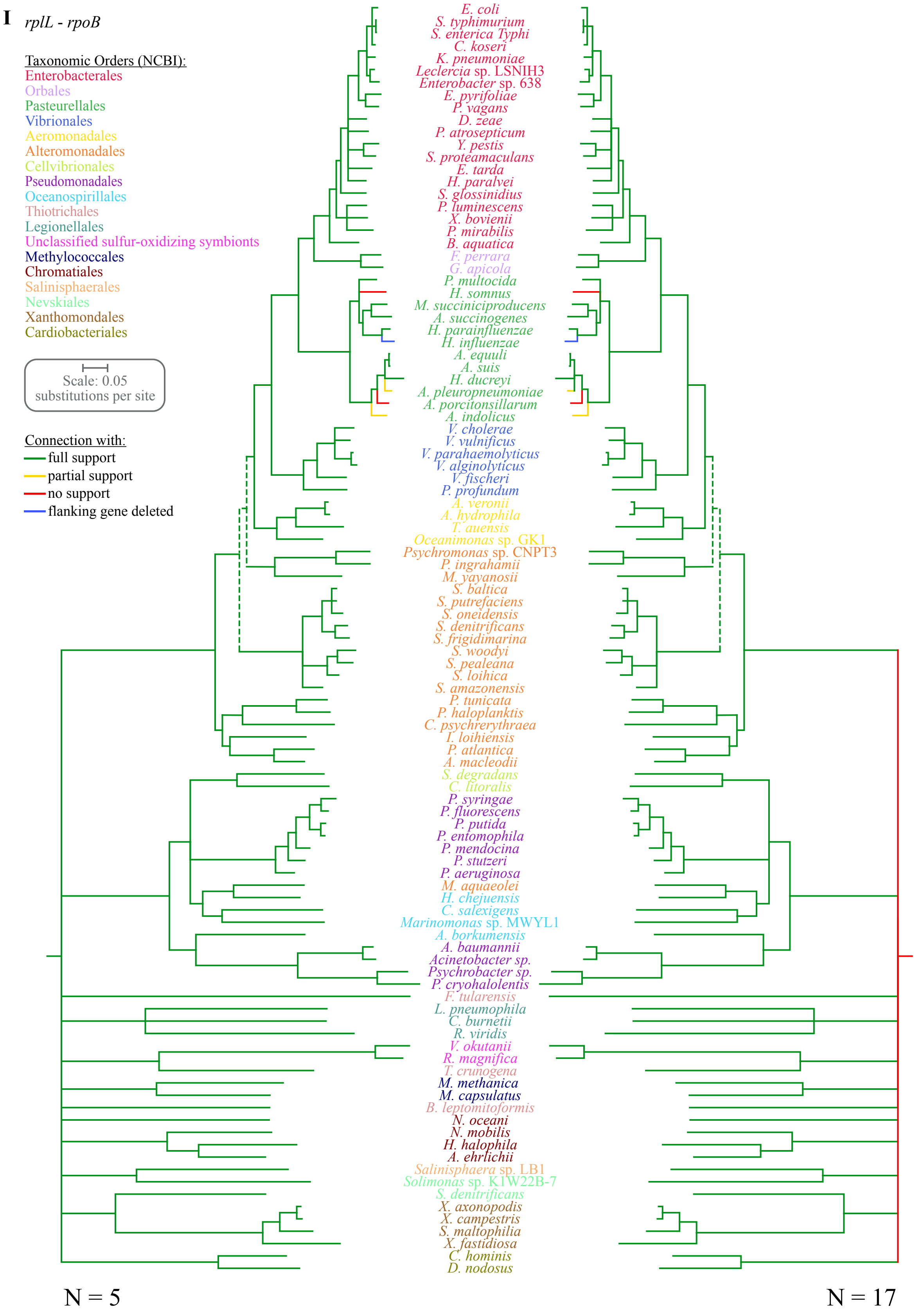
I
rplL - rpoB
Taxonomic Orders (NCBI):
Enterobacterales
Orbales
Pasteurellales
Vibrionales
Aeromonadales
Alteromonadales
Cellvibrionales
Pseudomonadales
Oceanospirillales
Thiotrichales
Legionellales
Unclassified sulfur-oxidizing symbionts
Methylococcales
Chromatiales
Salinisphaerales
Nevskiales
Xanthomondales
Cardiobacteriales
Scale: 0.05
substitutions per site
Connection with:
full support
partial support
no support
flanking gene deleted
E. coli
S. typhimurium
S. enterica Typhi
C. koseri
K. pneumoniae
Leclercia sp. LSNIH3
Enterobacter sp. 638
E. pyrifoliae
P. vagans
D. zeae
P. atrosepticum
Y. pestis
S. proteamaculans
E. tarda
H. paralvei
S. glossinidius
P. luminescens
X. bovienii
P. mirabilis
B. aquatica
F. perrara
G. apicola
P. multocida
H. somnus
M. succiniciproducens
A. succinogenes
H. parainfluenzae
H. influenzae
A. equuli
A. suis
H. ducreyi
A. pleuropneumoniae
A. porcitonsillarum
A. indolicus
V. cholerae
V. vulnificus
V. parahaemolyticus
V. alginolyticus
V. fischeri
P. profundum
A. veronii
A. hydrophila
T. auensis
Oceanimonas sp. GK1
Psychromonas sp. CNPT3
P. ingrahamii
M. yayanosii
S. baltica
S. putrefaciens
S. oneidensis
S. denitrificans
S. frigidimarina
S. woodyi
S. pealeana
S. loihica
S. amazonensis
P. tunicata
P. haloplanktis
C. psychrerythraea
I. loihiensis
P. atlantica
A. macleodii
S. degradans
C. litoralis
P. syringae
P. fluorescens
P. putida
P. entomophila
P. mendocina
P. stutzeri
P. aeruginosa
M. aquaeolei
H. chejuensis
C. salexigens
Marinomonas sp. MWYL1
A. borkumensis
A. baumannii
Acinetobacter sp.
Psychrobacter sp.
P. cryohalolentis
F. tularensis
L. pneumophila
C. burnetii
R. viridis
V. okutanii
R. magnifica
T. crunogena
M. methanica
M. capsulatus
B. leptomitoformis
N. oceani
N. mobilis
H. halophila
A. ehrlichii
Salinisphaera sp. LB1
Solimonas sp. K1W22B-7
S. denitrificans
X. axonopodis
X. campestris
S. maltophilia
X. fastidiosa
C. hominis
D. nodosus
N = 5
N = 17

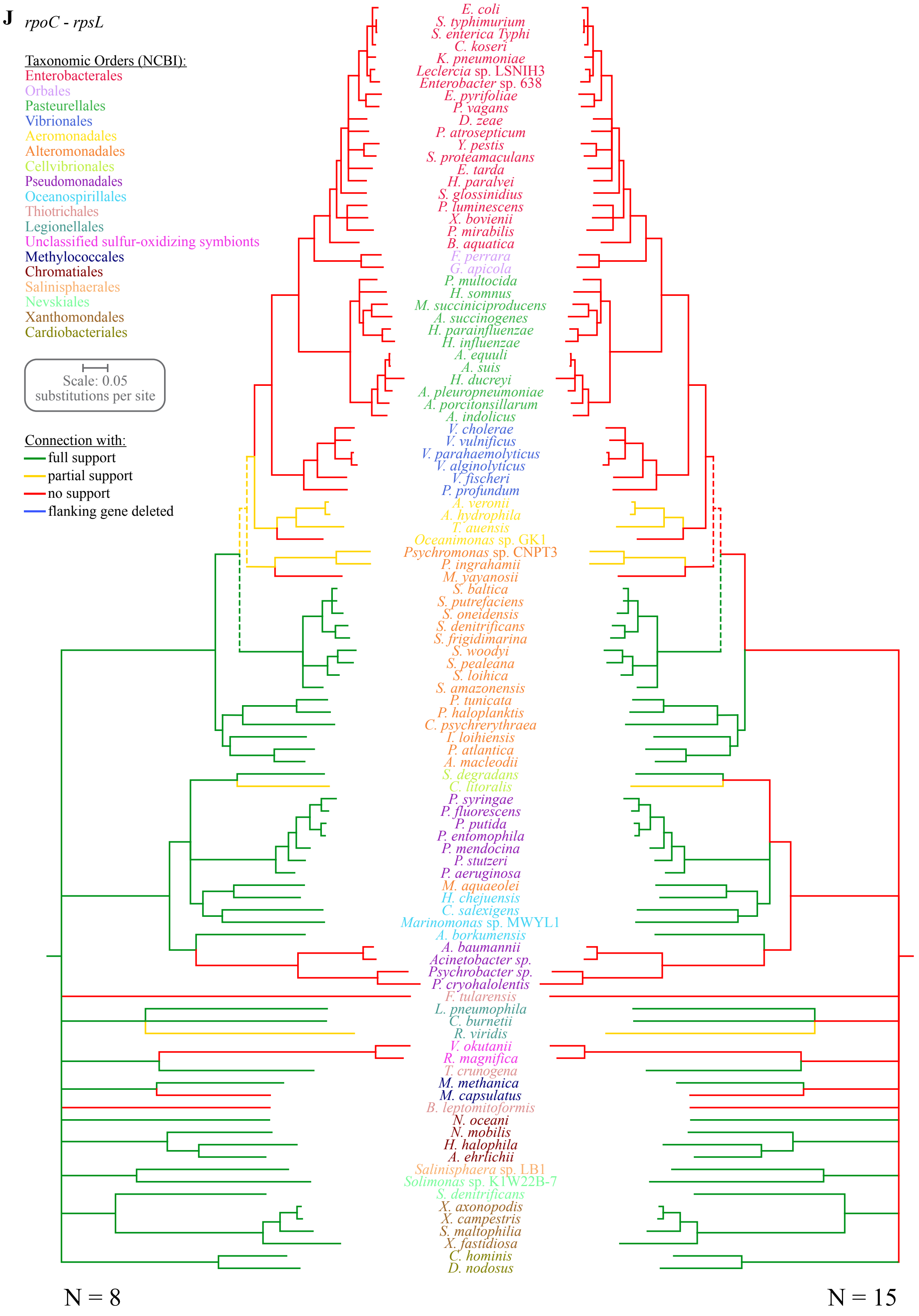
J
rpoC - rpsL
Taxonomic Orders (NCBI):
Enterobacterales
Orbales
Pasteurellales
Vibrionales
Aeromonadales
Alteromonadales
Cellvibrionales
Pseudomonadales
Oceanospirillales
Thiotrichales
Legionellales
Unclassified sulfur-oxidizing symbionts
Methylococcales
Chromatiales
Salinisphaerales
Nevskiales
Xanthomondales
Cardiobacteriales
Scale: 0.05
substitutions per site
Connection with:
full support
partial support
no support
flanking gene deleted
E. coli
S. typhimurium
S. enterica Typhi
C. koseri
K. pneumoniae
Leclercia sp. LSNIH3
Enterobacter sp. 638
E. pyrifoliae
P. vagans
D. zeae
P. atrosepticum
Y. pestis
S. proteamaculans
E. tarda
H. paralvei
S. glossinidius
P. luminescens
X. bovienii
P. mirabilis
B. aquatica
F. perrara
G. apicola
P. multocida
H. somnus
M. succiniciproducens
A. succinogenes
H. parainfluenzae
H. influenzae
A. equuli
A. suis
H. ducreyi
A. pleuropneumoniae
A. porcitonsillarum
A. indolicus
V. cholerae
V. vulnificus
V. parahaemolyticus
V. alginolyticus
V. fischeri
P. profundum
A. veronii
A. hydrophila
T. auensis
Oceanimonas sp. GK1
Psychromonas sp. CNPT3
P. ingrahamii
M. yayanosii
S. baltica
S. putrefaciens
S. oneidensis
S. denitrificans
S. frigidimarina
S. woodyi
S. pealeana
S. loihica
S. amazonensis
P. tunicata
P. haloplanktis
C. psychrerythraea
I. loihiensis
P. atlantica
A. macleodii
S. degradans
C. litoralis
P. syringae
P. fluorescens
P. putida
P. entomophila
P. mendocina
P. stutzeri
P. aeruginosa
M. aquaeolei
H. chejuensis
C. salexigens
Marinomonas sp. MWYL1
A. borkumensis
A. baumannii
Acinetobacter sp.
Psychrobacter sp.
P. cryohalolentis
F. tularensis
L. pneumophila
C. burnetii
R. viridis
V. okutanii
R. magnifica
T. crunogena
M. methanica
M. capsulatus
B. leptomitoformis
N. oceani
N. mobilis
H. halophila
A. ehrlichii
Salinisphaera sp. LB1
Solimonas sp. K1W22B-7
S. denitrificans
X. axonopodis
X. campestris
S. maltophilia
X. fastidiosa
C. hominis
D. nodosus
N = 8
N = 15

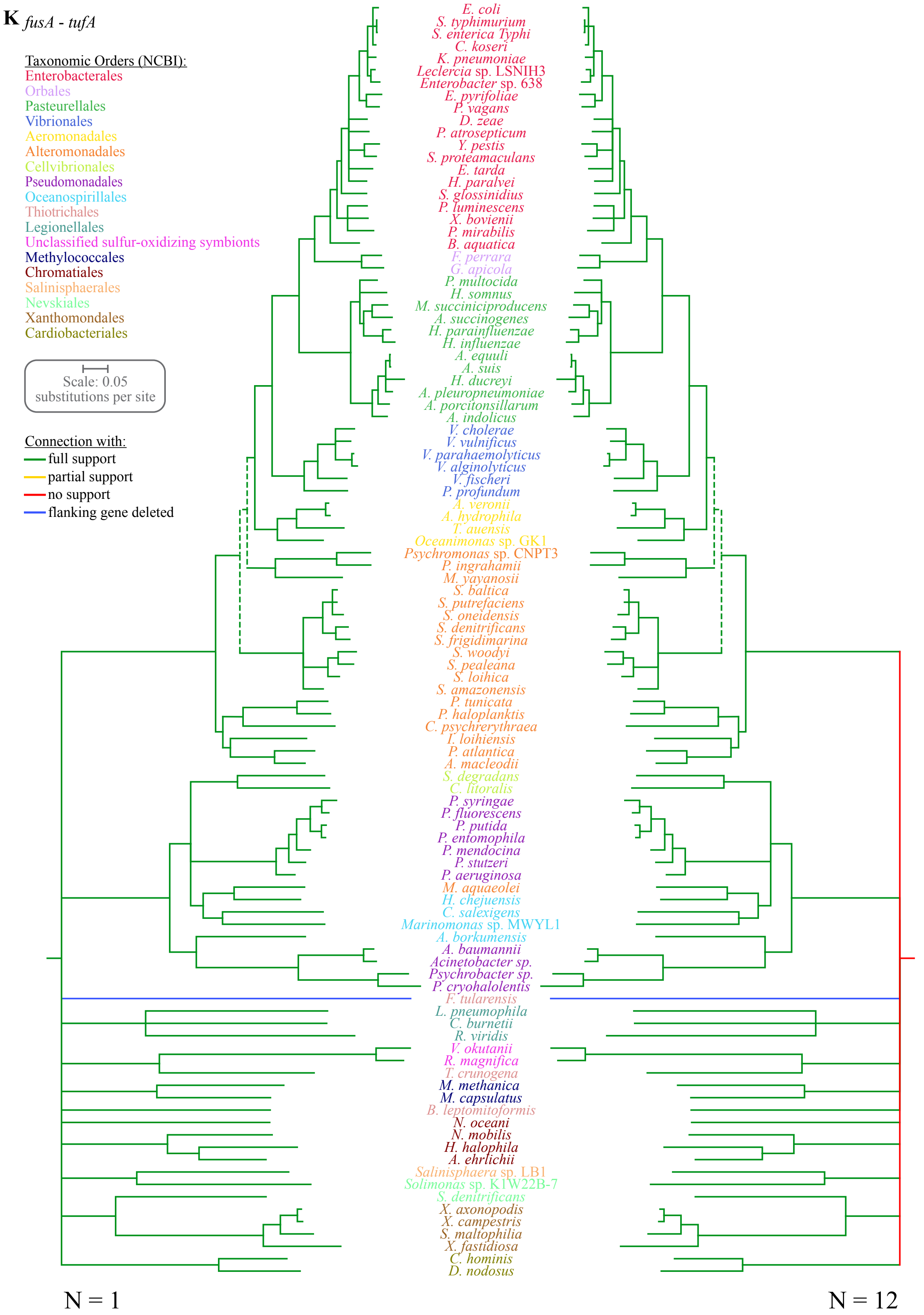
K fusA - tufA
Taxonomic Orders (NCBI):
Enterobacterales
Orbales
Pasteurellales
Vibrionales
Aeromonadales
Alteromonadales
Cellvibrionales
Pseudomonadales
Oceanospirillales
Thiotrichales
Legionellales
Unclassified sulfur-oxidizing symbionts
Methylococcales
Chromatiales
Salinisphaerales
Nevskiales
Xanthomondales
Cardiobacteriales
Scale: 0.05 substitutions per site
Connection with:
full support
partial support
no support
flanking gene deleted
E. coli
S. typhimurium
S. enterica Typhi
C. koseri
K. pneumoniae
Leclercia sp. LSNIH3
Enterobacter sp. 638
E. pyrifoliae
P. vagans
D. zeae
P. atrosepticum
Y. pestis
S. proteamaculans
E. tarda
H. paralvei
S. glossinidius
P. luminescens
X. bovienii
P. mirabilis
B. aquatica
F. perrara
G. apicola
P. multocida
H. somnus
M. succiniciproducens
A. succinogenes
H. parainfluenzae
H. influenzae
A. equuli
A. suis
H. ducreyi
A. pleuropneumoniae
A. porcitonsillarum
A. indolicus
V. cholerae
V. vulnificus
V. parahaemolyticus
V. alginolyticus
V. fischeri
P. profundum
A. veronii
A. hydrophila
T. auensis
Oceanimonas sp. GK1
Psychromonas sp. CNPT3
P. ingrahamii
M. yayanosii
S. baltica
S. putrefaciens
S. oneidensis
S. denitrificans
S. frigidimarina
S. woodyi
S. pealeana
S. loihica
S. amazonensis
P. tunicata
P. haloplanktis
C. psychrerythraea
I. loihiensis
P. atlantica
A. macleodii
S. degradans
C. litoralis
P. syringae
P. fluorescens
P. putida
P. entomophila
P. mendocina
P. stutzeri
P. aeruginosa
M. aquaeolei
H. chejuensis
C. salexigens
Marinomonas sp. MWYL1
A. borkumensis
A. baumannii
Acinetobacter sp.
Psychrobacter sp.
P. cryohalolentis
F. tularensis
L. pneumophila
C. burnetii
R. viridis
V. okutanii
R. magnifica
T. crunogena
M. methanica
M. capsulatus
B. leptomitoformis
N. oceani
N. mobilis
H. halophila
A. ehrlichii
Salinisphaera sp. LB1
Solimonas sp. K1W22B-7
S. denitrificans
X. axonopodis
X. campestris
S. maltophilia
X. fastidiosa
C. hominis
D. nodosus
N = 1
N = 12

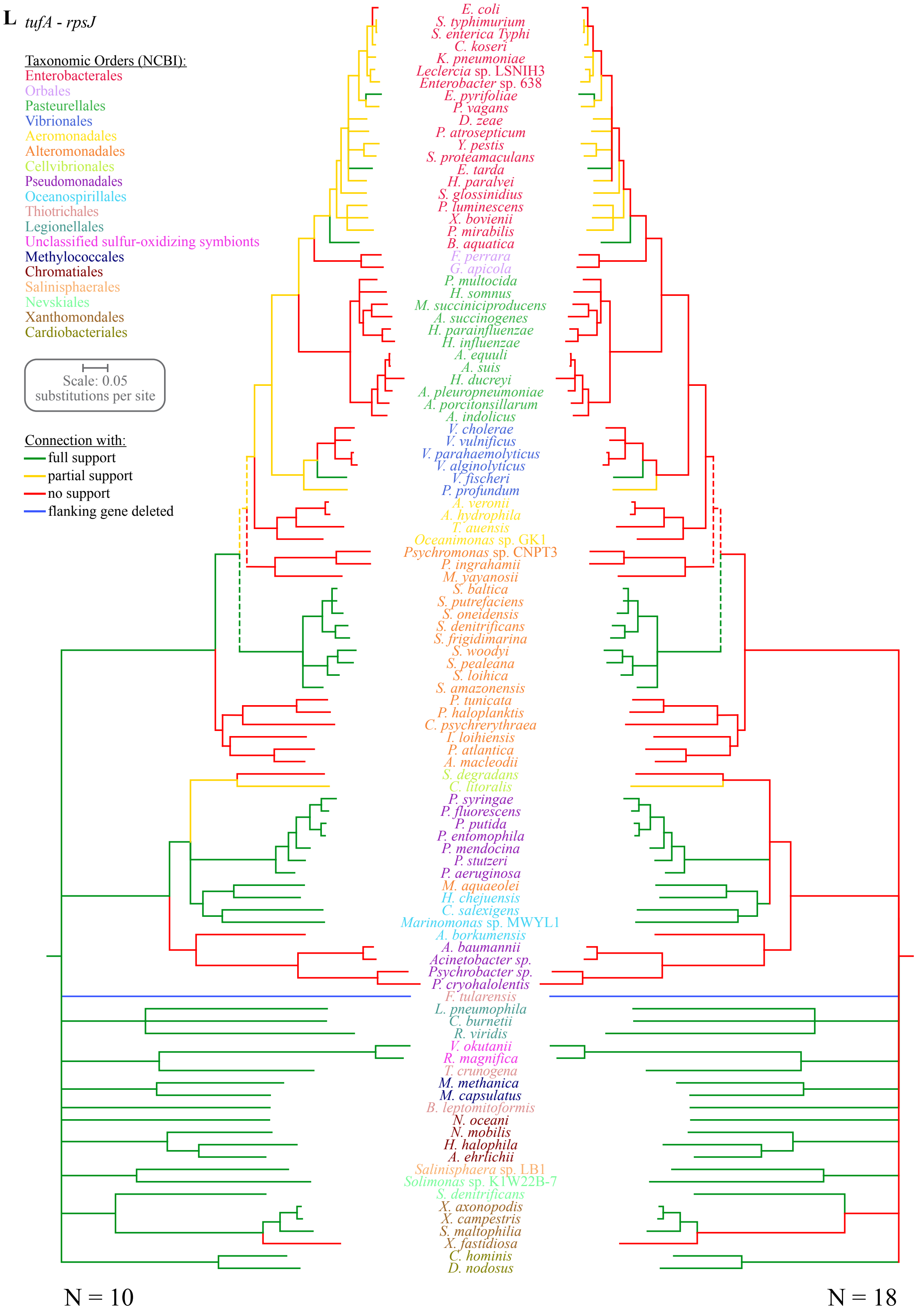
L tufA - rpsJ
Taxonomic Orders (NCBI):
Enterobacterales
Orbales
Pasteurellales
Vibrionales
Aeromonadales
Alteromonadales
Cellvibrionales
Pseudomonadales
Oceanospirillales
Thiotrichales
Legionellales
Unclassified sulfur-oxidizing symbionts
Methylococcales
Chromatiales
Salinisphaerales
Nevskiales
Xanthomondales
Cardiobacteriales
Scale: 0.05
substitutions per site
Connection with:
full support
partial support
no support
flanking gene deleted
E. coli
S. typhimurium
S. enterica Typhi
C. koseri
K. pneumoniae
Leclercia sp. LSNIH3
Enterobacter sp. 638
E. pyrifoliae
P. vagans
D. zeae
P. atrosepticum
Y. pestis
S. proteamaculans
E. tarda
H. paralvei
S. glossinidius
P. luminescens
X. bovienii
P. mirabilis
B. aquatica
F. perrara
G. apicola
P. multocida
H. somnus
M. succiniciproducens
A. succinogenes
H. parainfluenzae
H. influenzae
A. equuli
A. suis
H. ducreyi
A. pleuropneumoniae
A. porcitonsillarum
A. indolicus
V. cholerae
V. vulnificus
V. parahaemolyticus
V. alginolyticus
V. fischeri
P. profundum
A. veronii
A. hydrophila
T. auensis
Oceanimonas sp. GK1
Psychromonas sp. CNPT3
P. ingrahamii
M. yayanosii
S. baltica
S. putrefaciens
S. oneidensis
S. denitrificans
S. frigidimarina
S. woodyi
S. pealeana
S. loihica
S. amazonensis
P. tunicata
P. haloplanktis
C. psychrerythraea
I. loihiensis
P. atlantica
A. macleodii
S. degradans
C. litoralis
P. syringae
P. fluorescens
P. putida
P. entomophila
P. mendocina
P. stutzeri
P. aeruginosa
M. aquaeolei
H. chejuensis
C. salexigens
Marinomonas sp. MWYL1
A. borkumensis
A. baumannii
Acinetobacter sp.
Psychrobacter sp.
P. cryohalolentis
F. tularensis
L. pneumophila
C. burnetii
R. viridis
V. okutanii
R. magnifica
T. crunogena
M. methanica
M. capsulatus
B. leptomitoformis
N. oceani
N. mobilis
H. halophila
A. ehrlichii
Salinisphaera sp. LB1
Solimonas sp. K1W22B-7
S. denitrificans
X. axonopodis
X. campestris
S. maltophilia
X. fastidiosa
C. hominis
D. nodosus
N = 10
N = 18

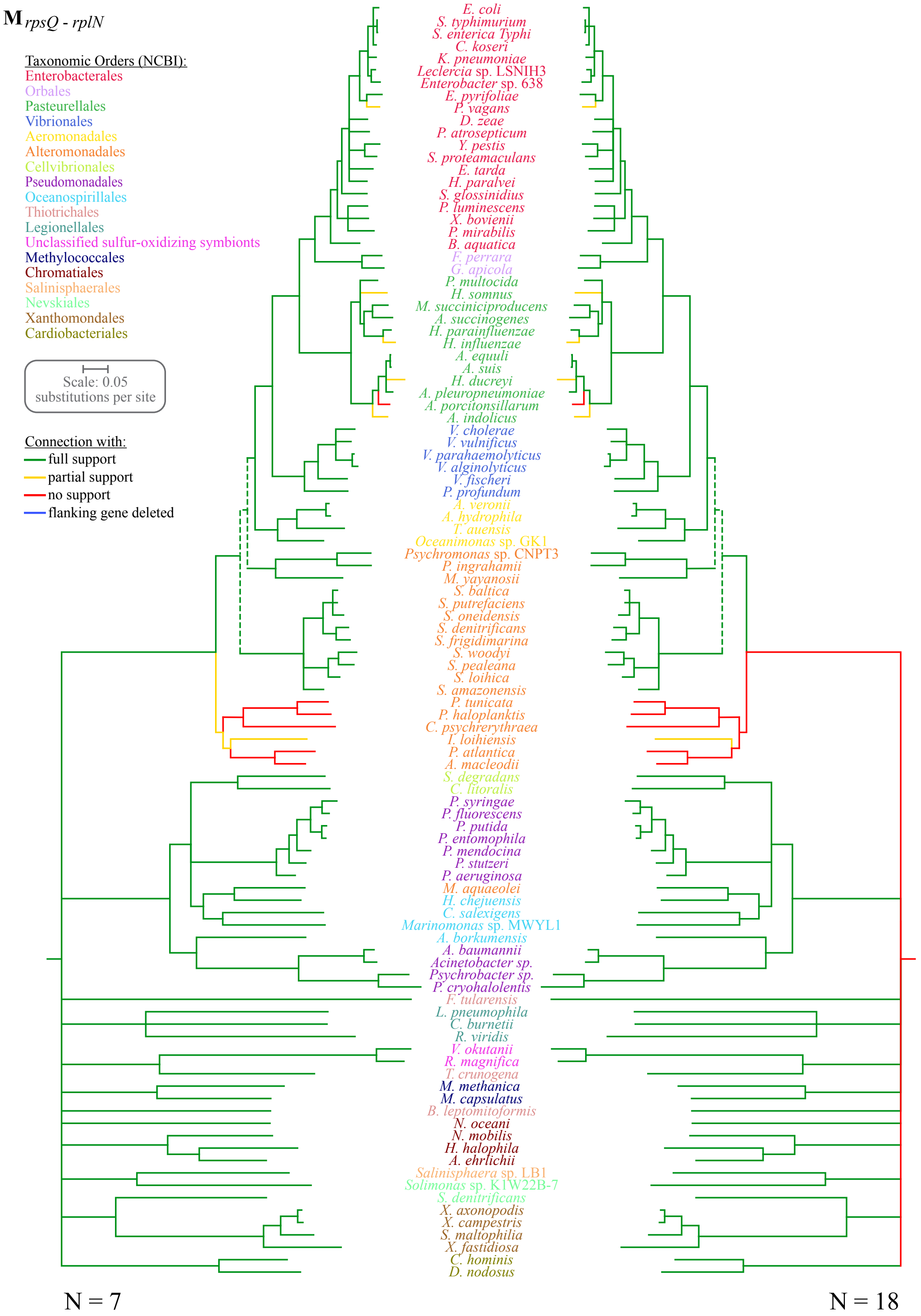

M rpsQ - rplN
Taxonomic Orders (NCBI):
Enterobacterales
Orbales
Pasteurellales
Vibrionales
Aeromonadales
Alteromonadales
Cellvibrionales
Pseudomonadales
Oceanospirillales
Thiotrichales
Legionellales
Unclassified sulfur-oxidizing symbionts
Methylococcales
Chromatiales
Salinisphaerales
Nevskiales
Xanthomonadales
Cardiobacteriales
Scale: 0.05
substitutions per site
Connection with:
full support
partial support
no support
flanking gene deleted
E. coli
S. typhimurium
S. enterica Typhi
C. koseri
K. pneumoniae
Leclercia sp. LSNIH3
Enterobacter sp. 638
E. pyrifoliae
P. vagans
D. zeae
P. atrosepticum
Y. pestis
S. proteamaculans
E. tarda
H. paralvei
S. glossinidius
P. luminescens
X. bovienii
P. mirabilis
B. aquatica
F. perrara
G. apicola
P. multocida
H. somnus
M. succiniciproducens
A. succinogenes
H. parainfluenzae
H. influenzae
A. equuli
A. suis
H. ducreyi
A. pleuropneumoniae
A. porcitonsillarum
A. indolicus
V. cholerae
V. vulnificus
V. parahaemolyticus
V. alginolyticus
V. fischeri
P. profundum
A. veronii
A. hydrophila
T. auensis
Oceanimonas sp. GK1
Psychromonas sp. CNPT3
P. ingrahamii
M. yayanosii
S. baltica
S. putrefaciens
S. oneidensis
S. denitrificans
S. frigidimarina
S. woodyi
S. pealeana
S. loihica
S. amazonensis
P. tunicata
P. haloplanktis
C. psychrerythraea
I. loihiensis
P. atlantica
A. macleodii
S. degradans
C. litoralis
P. syringae
P. fluorescens
P. putida
P. entomophila
P. mendocina
P. stutzeri
P. aeruginosa
M. aquaeolei
H. chejuensis
C. salexigens
Marinomonas sp. MWYL1
A. borkumensis
A. baumannii
Acinetobacter sp.
Psychrobacter sp.
P. cryohalolentis
F. tularensis
L. pneumophila
C. burnetii
R. viridis
V. okutanii
R. magnifica
T. crunogena
M. methanica
M. capsulatus
B. leptomitoformis
N. oceani
N. mobilis
H. halophila
A. ehrlichii
Salinisphaera sp. LB1
Solimonas sp. K1W22B-7
S. denitrificans
X. axonopodis
X. campestris
S. maltophilia
X. fastidiosa
C. hominis
D. nodosus
N = 7
N = 18

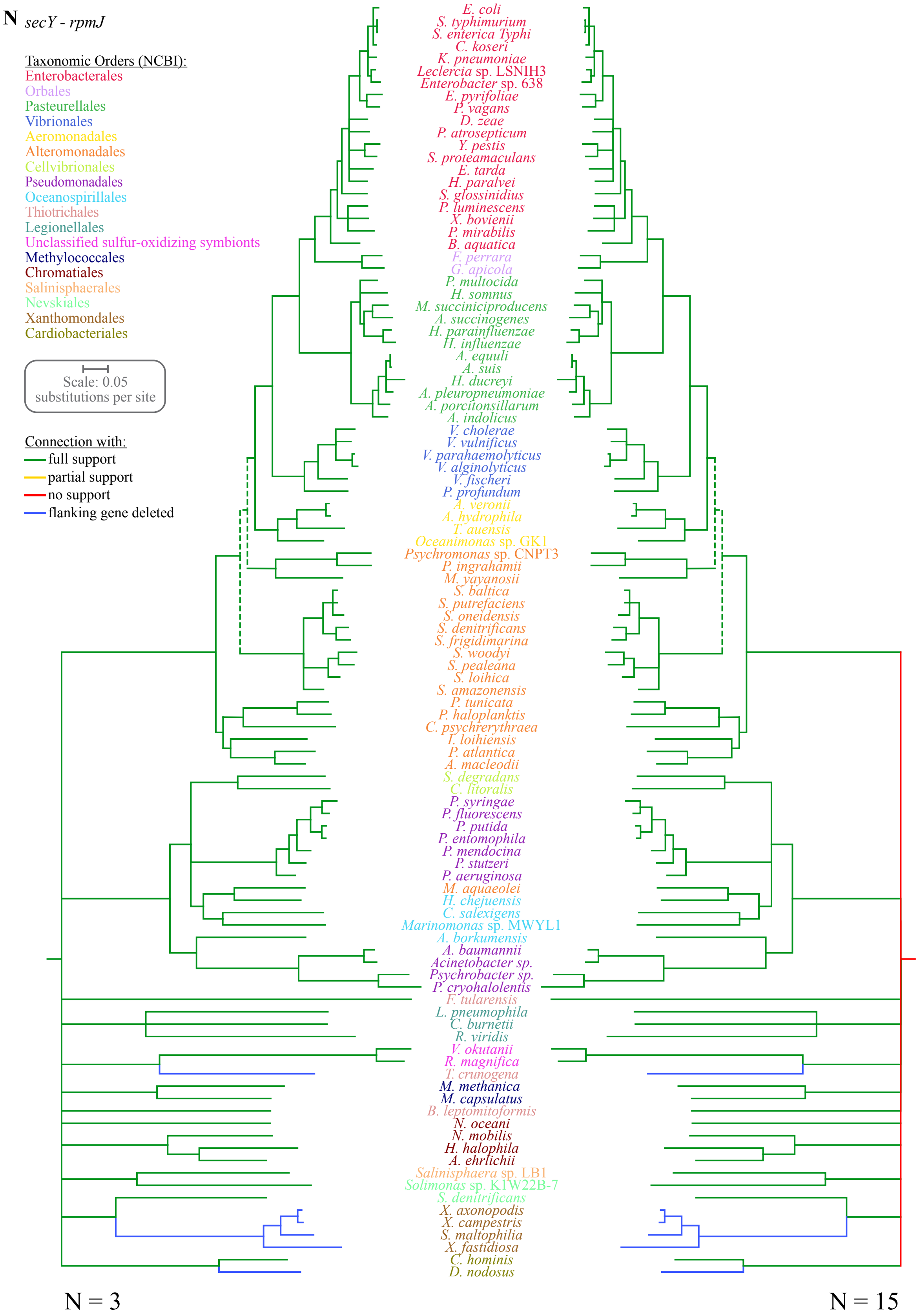
N
secY - rpmJ
Taxonomic Orders (NCBI):
Enterobacterales
Orbales
Pasteurellales
Vibrionales
Aeromonadales
Alteromonadales
Cellvibrionales
Pseudomonadales
Oceanospirillales
Thiotrichales
Legionellales
Unclassified sulfur-oxidizing symbionts
Methylococcales
Chromatiales
Salinisphaerales
Nevskiales
Xanthomondales
Cardiobacteriales
Scale: 0.05
substitutions per site
Connection with:
full support
partial support
no support
flanking gene deleted
E. coli
S. typhimurium
S. enterica Typhi
C. koseri
K. pneumoniae
Leclercia sp. LSNIH3
Enterobacter sp. 638
E. pyrifoliae
P. vagans
D. zeae
P. atrosepticum
Y. pestis
S. proteamaculans
E. tarda
H. paralvei
S. glossinidius
P. luminescens
X. bovienii
P. mirabilis
B. aquatica
F. perrara
G. apicola
P. multocida
H. somnus
M. succiniciproducens
A. succinogenes
H. parainfluenzae
H. influenzae
A. equuli
A. suis
H. ducreyi
A. pleuropneumoniae
A. porcitonsillarum
A. indolicus
V. cholerae
V. vulnificus
V. parahaemolyticus
V. alginolyticus
V. fischeri
P. profundum
A. veronii
A. hydrophila
T. auensis
Oceanimonas sp. GK1
Psychromonas sp. CNPT3
P. ingrahamii
M. yayanosii
S. baltica
S. putrefaciens
S. oneidensis
S. denitrificans
S. frigidimarina
S. woodyi
S. pealeana
S. loihica
S. amazonensis
P. tunicata
P. haloplanktis
C. psychrerythraea
I. loihiensis
P. atlantica
A. macleodii
S. degradans
C. litoralis
P. syringae
P. fluorescens
P. putida
P. entomophila
P. mendocina
P. stutzeri
P. aeruginosa
M. aquaeolei
H. chejuensis
C. salexigens
Marinomonas sp. MWYL1
A. borkumensis
A. baumannii
Acinetobacter sp.
Psychrobacter sp.
P. cryohalolentis
F. tularensis
L. pneumophila
C. burnetii
R. viridis
V. okutanii
R. magnifica
T. crunogena
M. methanica
M. capsulatus
B. leptomitoformis
N. oceani
N. mobilis
H. halophila
A. ehrlichii
Salinisphaera sp. LB1
Solimonas sp. K1W22B-7
S. denitrificans
X. axonopodis
X. campestris
S. maltophilia
X. fastidiosa
C. hominis
D. nodosus
N = 3
N = 15

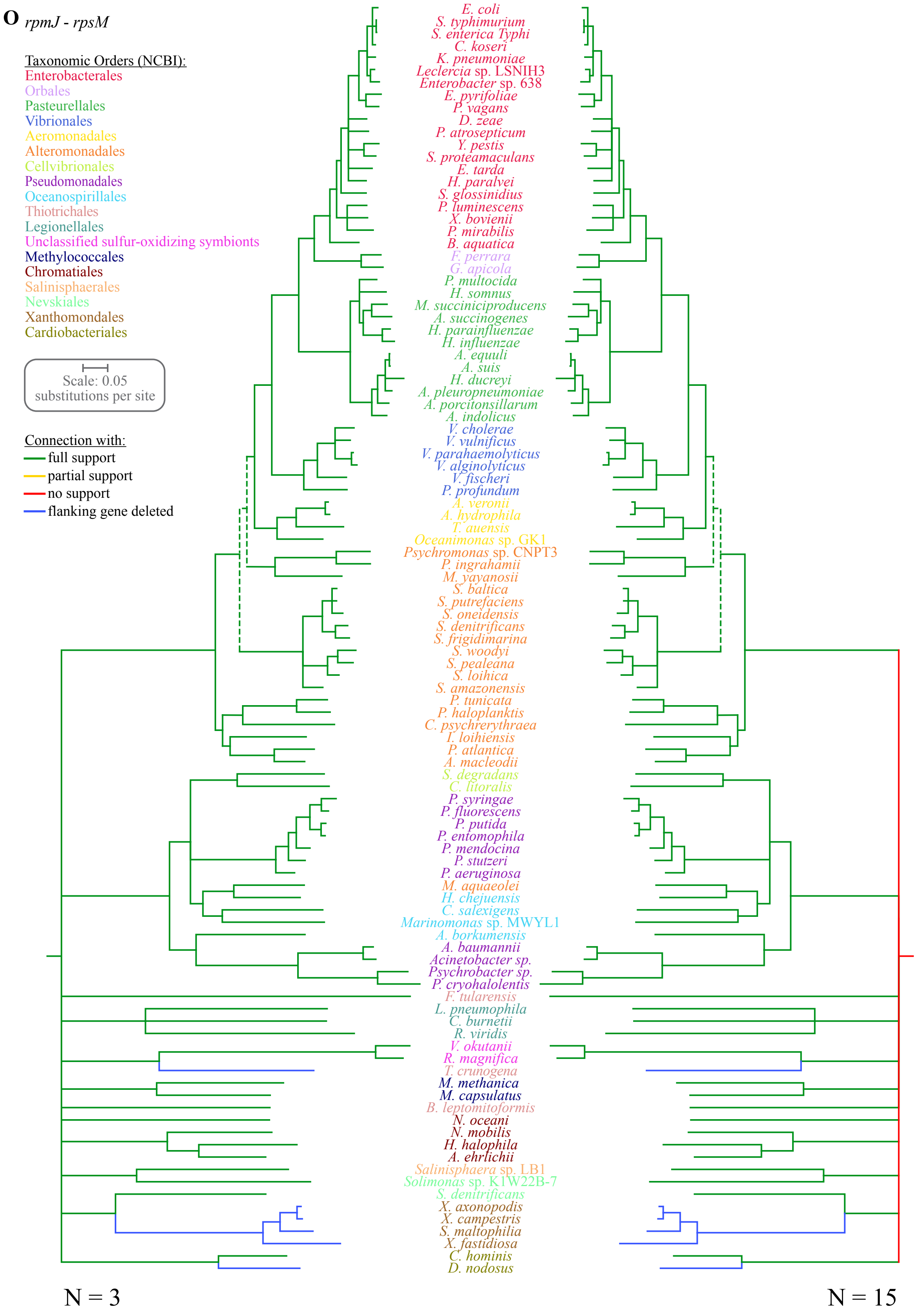
O rpmJ - rpsM
Taxonomic Orders (NCBI):
Enterobacterales
Orbales
Pasteurellales
Vibrionales
Aeromonadales
Alteromonadales
Cellvibrionales
Pseudomonadales
Oceanospirillales
Thiotrichales
Legionellales
Unclassified sulfur-oxidizing symbionts
Methylococcales
Chromatiales
Salinisphaerales
Nevskiales
Xanthomondales
Cardiobacteriales
Scale: 0.05
substitutions per site
Connection with:
full support
partial support
no support
flanking gene deleted
E. coli
S. typhimurium
S. enterica Typhi
C. koseri
K. pneumoniae
Leclercia sp. LSNIH3
Enterobacter sp. 638
E. pyrifoliae
P. vagans
D. zeae
P. atrosepticum
Y. pestis
S. proteamaculans
E. tarda
H. paralvei
S. glossinidius
P. luminescens
X. bovienii
P. mirabilis
B. aquatica
F. perrara
G. apicola
P. multocida
H. somnus
M. succiniciproducens
A. succinogenes
H. parainfluenzae
H. influenzae
A. equuli
A. suis
H. ducreyi
A. pleuropneumoniae
A. porcitonsillarum
A. indolicus
V. cholerae
V. vulnificus
V. parahaemolyticus
V. alginolyticus
V. fischeri
P. profundum
A. veronii
A. hydrophila
T. auensis
Oceanimonas sp. GK1
Psychromonas sp. CNPT3
P. ingrahamii
M. yayanosii
S. baltica
S. putrefaciens
S. oneidensis
S. denitrificans
S. frigidimarina
S. woodyi
S. pealeana
S. loihica
S. amazonensis
P. tunicata
P. haloplanktis
C. psychrerythraea
I. loihiensis
P. atlantica
A. macleodii
S. degradans
C. litoralis
P. syringae
P. fluorescens
P. putida
P. entomophila
P. mendocina
P. stutzeri
P. aeruginosa
M. aquaeolei
H. chejuensis
C. salexigens
Marinomonas sp. MWYL1
A. borkumensis
A. baumannii
Acinetobacter sp.
Psychrobacter sp.
P. cryohalolentis
F. tularensis
L. pneumophila
C. burnetii
R. viridis
V. okutanii
R. magnifica
T. crunogena
M. methanica
M. capsulatus
B. leptomitoformis
N. oceani
N. mobilis
H. halophila
A. ehrlichii
Salinisphaera sp. LB1
Solimonas sp. K1W22B-7
S. denitrificans
X. axonopodis
X. campestris
S. maltophilia
X. fastidiosa
C. hominis
D. nodosus
N = 3
N = 15

FIG. S14. Decision trees used in the reconstruction of the ancestral operon cluster for all gene pairs that are not fully conserved throughout the *Gammaproteobacteria* used in this study. The number of minimal state changes (N) of each gene pair was determined for the case that the gene pair was connected (left tree) or disconnected (right tree) within the last common ancestor of the displayed species. The definition of potential state changes is shown in supplementary fig. S13.

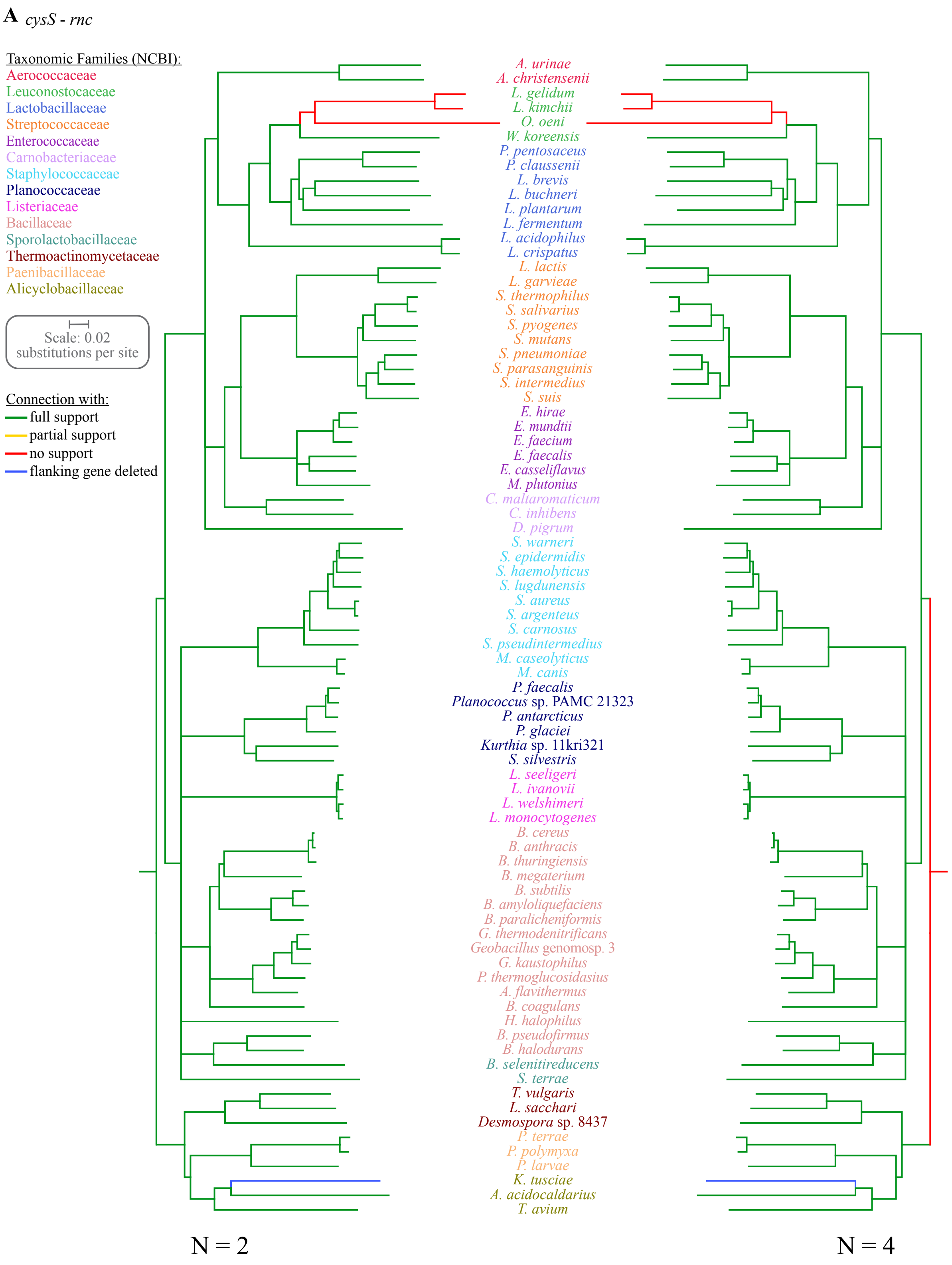

A cysS - rnc
Taxonomic Families (NCBI):
Aerococcaceae
Leuconostocaceae
Lactobacillaceae
Streptococcaceae
Enterococcaceae
Carnobacteriaceae
Staphylococcaceae
Planococcaceae
Listeriaceae
Bacillaceae
Sporolactobacillaceae
Thermoactinomycetaceae
Paenibacillaceae
Alicyclobacillaceae
Scale: 0.02 substitutions per site
Connection with:
full support
partial support
no support
flanking gene deleted
A. urinae
A. christensenii
L. gelidum
L. kimchii
O. oeni
W. koreensis
P. pentosaceus
P. claussenii
L. brevis
L. buchneri
L. plantarum
L. fermentum
L. acidophilus
L. crispatus
L. lactis
L. garvieae
S. thermophilus
S. salivarius
S. pyogenes
S. mutans
S. pneumoniae
S. parasanguinis
S. intermedius
S. suis
E. hirae
E. mundtii
E. faecium
E. faecalis
E. casseliflavus
M. plutonius
C. maltaromaticum
C. inhibens
D. pigrum
S. warneri
S. epidermidis
S. haemolyticus
S. lugdunensis
S. aureus
S. argenteus
S. carnosus
S. pseudintermedius
M. caseolyticus
M. canis
P. faecalis
Planococcus sp. PAMC 21323
P. antarcticus
P. glaciei
Kurthia sp. 11kri321
S. silvestris
L. seeligeri
L. ivanovii
L. welshimeri
L. monocytogenes
B. cereus
B. anthracis
B. thuringiensis
B. megaterium
B. subtilis
B. amyloliquefaciens
B. paralicheniformis
G. thermodenitrificans
Geobacillus genomosp. 3
G. kaustophilus
P. thermoglucosidasius
A. flavithermus
B. coagulans
H. halophilus
B. pseudofirmus
B. halodurans
B. selenitireducens
S. terrae
T. vulgaris
L. sacchari
Desmospora sp. 8437
P. terrae
P. polymyxa
P. larvae
K. tusciae
A. acidocaldarius
T. avium
N = 2
N = 4

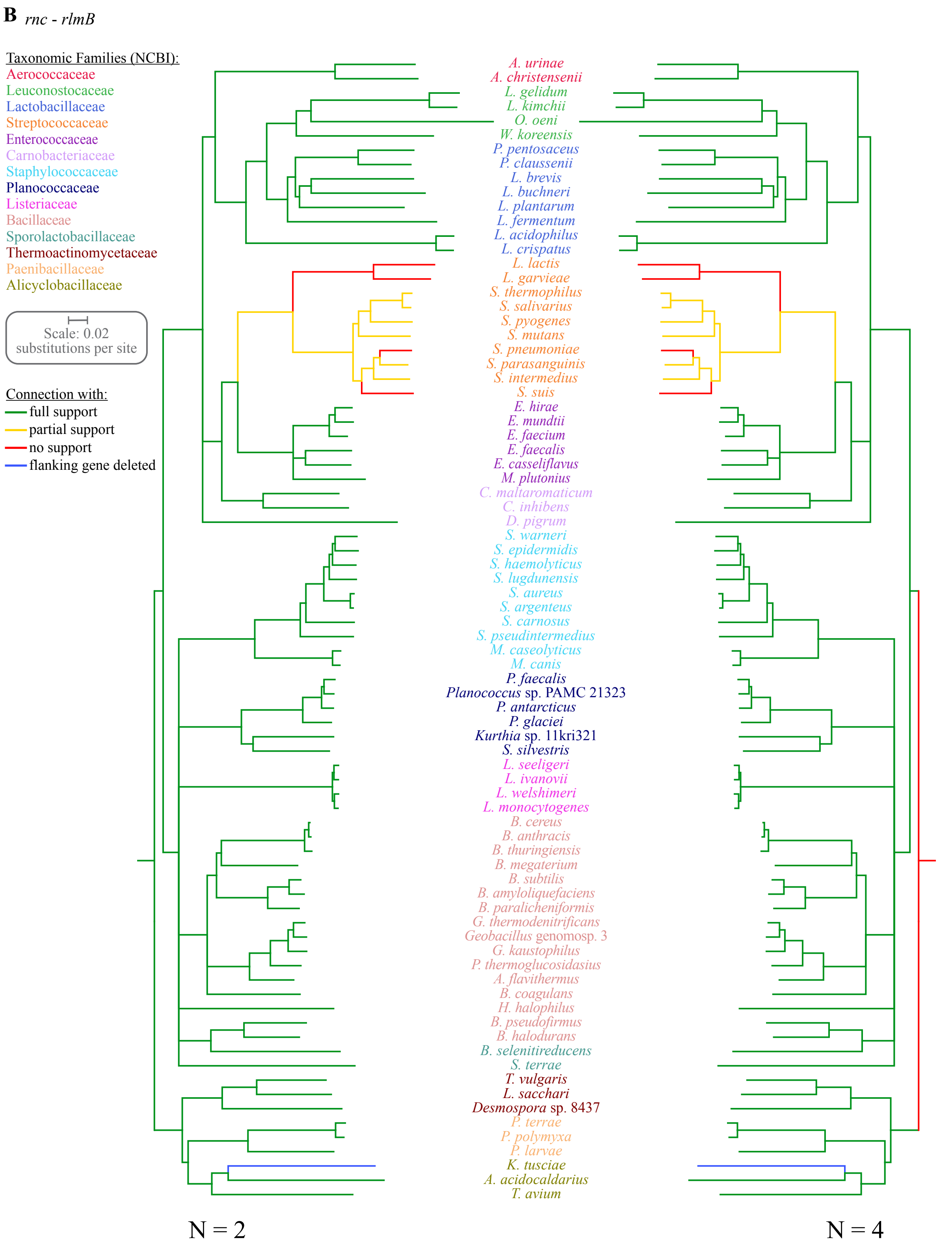
B rnc - rlmB
Taxonomic Families (NCBI):
Aerococcaceae
Leuconostocaceae
Lactobacillaceae
Streptococcaceae
Enterococcaceae
Carnobacteriaceae
Staphylococcaceae
Planococcaceae
Listeriaceae
Bacillaceae
Sporolactobacillaceae
Thermoactinomycetaceae
Paenibacillaceae
Alicyclobacillaceae
Scale: 0.02 substitutions per site
Connection with:
full support
partial support
no support
flanking gene deleted
A. urinae
A. christensenii
L. gelidum
L. kimchii
O. oeni
W. koreensis
P. pentosaceus
P. claussenii
L. brevis
L. buchneri
L. plantarum
L. fermentum
L. acidophilus
L. crispatus
L. lactis
L. garvieae
S. thermophilus
S. salivarius
S. pyogenes
S. mutans
S. pneumoniae
S. parasanguinis
S. intermedius
S. suis
E. hirae
E. mundtii
E. faecium
E. faecalis
E. casseliflavus
M. plutonius
C. maltaromaticum
C. inhibens
D. pigrum
S. warneri
S. epidermidis
S. haemolyticus
S. lugdunensis
S. aureus
S. argenteus
S. carnosus
S. pseudintermedius
M. caseolyticus
M. canis
P. faecalis
Planococcus sp. PAMC 21323
P. antarcticus
P. glaciei
Kurthia sp. 11kri321
S. silvestris
L. seeligeri
L. ivanovii
L. welshimeri
L. monocytogenes
B. cereus
B. anthracis
B. thuringiensis
B. megaterium
B. subtilis
B. amyloliquefaciens
B. paralicheniformis
G. thermodenitrificans
Geobacillus genomosp. 3
G. kaustophilus
P. thermoglucosidasius
A. flavithermus
B. coagulans
H. halophilus
B. pseudofirmus
B. halodurans
B. selenitireducens
S. terrae
T. vulgaris
L. sacchari
Desmospora sp. 8437
P. terrae
P. polymyxa
P. larvae
K. tusciae
A. acidocaldarius
T. avium
N = 2
N = 4

C *rlmB - orf1*

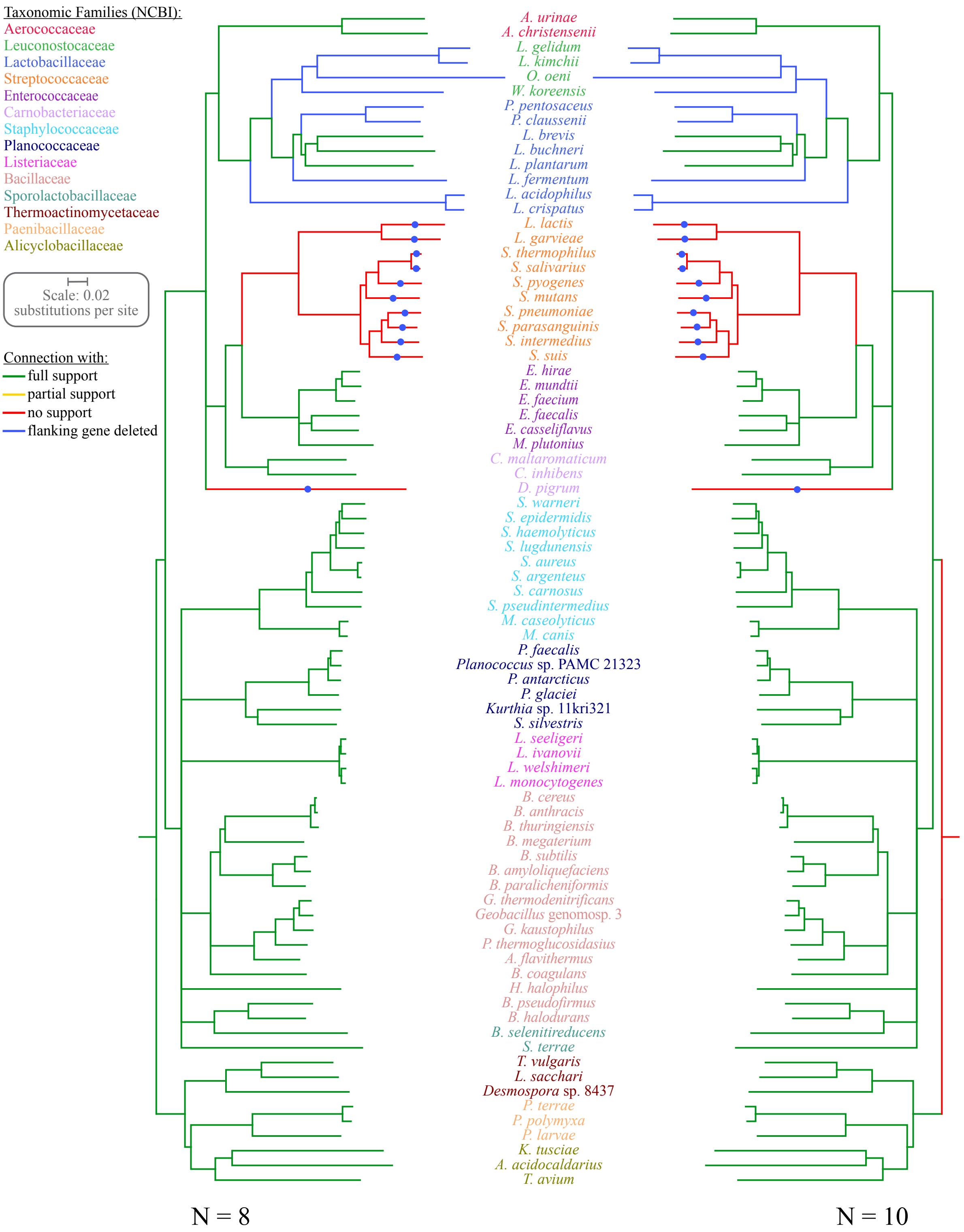

Taxonomic Families (NCBI):
Aerococcaceae
Leuconostocaceae
Lactobacillaceae
Streptococcaceae
Enterococcaceae
Carnobacteriaceae
Staphylococcaceae
Planococcaceae
Listeriaceae
Bacillaceae
Sporolactobacillaceae
Thermoactinomycetaceae
Paenibacillaceae
Alicyclobacillaceae
Scale: 0.02 substitutions per site
Connection with:
full support
partial support
no support
flanking gene deleted
A. urinae
A. christensenii
L. gelidum
L. kimchii
O. oeni
W. koreensis
P. pentosaceus
P. claussenii
L. brevis
L. buchneri
L. plantarum
L. fermentum
L. acidophilus
L. crispatus
L. lactis
L. garvieae
S. thermophilus
S. salivarius
S. pyogenes
S. mutans
S. pneumoniae
S. parasanguinis
S. intermedius
S. suis
E. hirae
E. mundtii
E. faecium
E. faecalis
E. casseliflavus
M. plutonius
C. maltaromaticum
C. inhibens
D. pigrum
S. warneri
S. epidermidis
S. haemolyticus
S. lugdunensis
S. aureus
S. argenteus
S. carnosus
S. pseudintermedius
M. caseolyticus
M. canis
P. faecalis
Planococcus sp. PAMC 21323
P. antarcticus
P. glaciei
Kurthia sp. 11kri321
S. silvestris
L. seeligeri
L. ivanovii
L. welshimeri
L. monocytogenes
B. cereus
B. anthracis
B. thuringiensis
B. megaterium
B. subtilis
B. amyloliquefaciens
B. paralicheniformis
G. thermodenitrificans
Geobacillus genomosp. 3
G. kaustophilus
P. thermoglucosidasius
A. flavithermus
B. coagulans
H. halophilus
B. pseudofirmus
B. halodurans
B. selenitireducens
S. terrae
T. vulgaris
L. sacchari
Desmospora sp. 8437
P. terrae
P. polymyxa
P. larvae
K. tusciae
A. acidocaldarius
T. avium
N = 8
N = 10

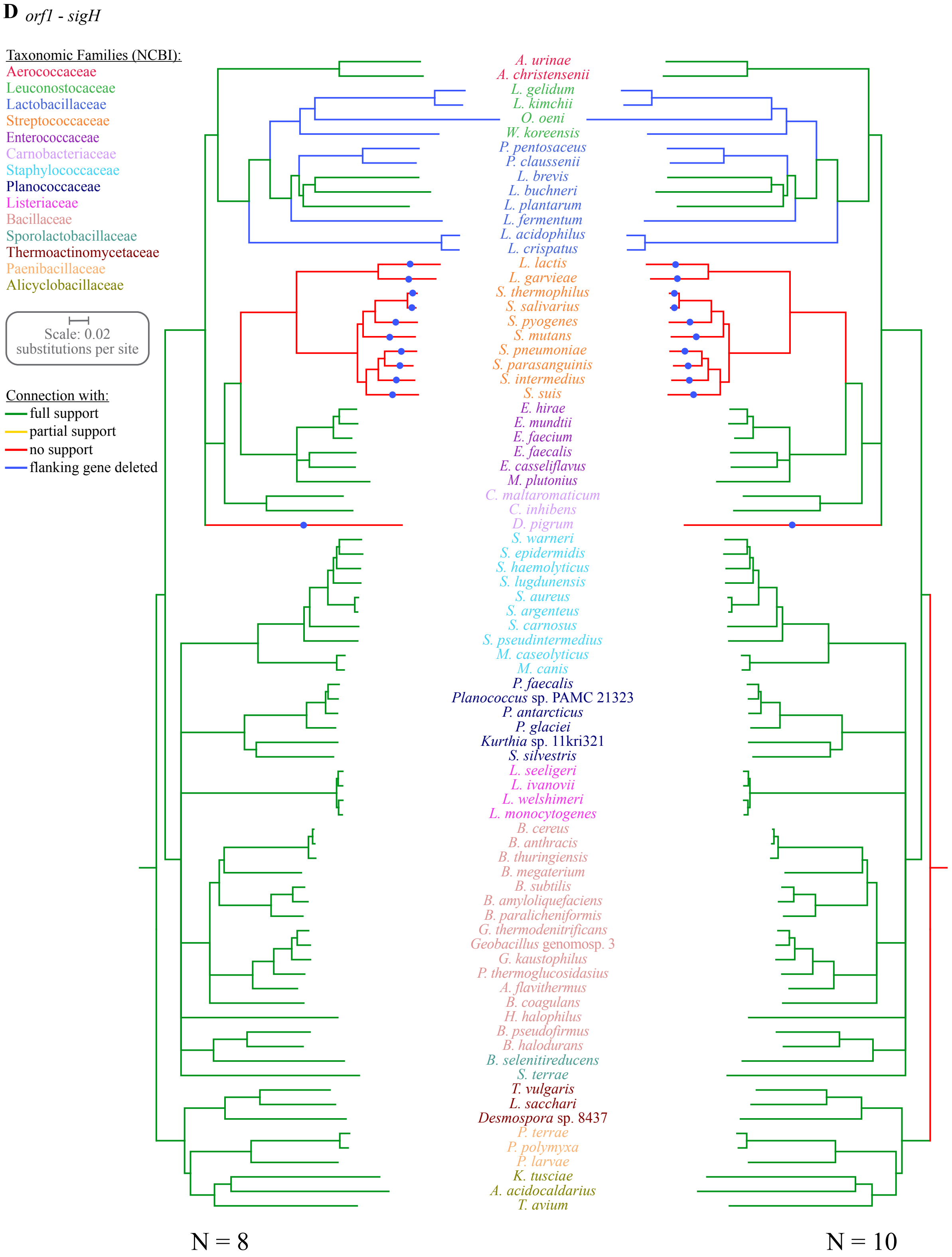
D orf1 - sigH
Taxonomic Families (NCBI):
Aerococcaceae
Leuconostocaceae
Lactobacillaceae
Streptococcaceae
Enterococcaceae
Carnobacteriaceae
Staphylococcaceae
Planococcaceae
Listeriaceae
Bacillaceae
Sporolactobacillaceae
Thermoactinomycetaceae
Paenibacillaceae
Alicyclobacillaceae
Scale: 0.02
substitutions per site
Connection with:
full support
partial support
no support
flanking gene deleted
A. urinae
A. christensenii
L. gelidum
L. kimchii
O. oeni
W. koreensis
P. pentosaceus
P. claussenii
L. brevis
L. buchneri
L. plantarum
L. fermentum
L. acidophilus
L. crispatus
L. lactis
L. garvieae
S. thermophilus
S. salivarius
S. pyogenes
S. mutans
S. pneumoniae
S. parasanguinis
S. intermedius
S. suis
E. hirae
E. mundtii
E. faecium
E. faecalis
E. casseliflavus
M. plutonius
C. maltaromaticum
C. inhibens
D. pigrum
S. warneri
S. epidermidis
S. haemolyticus
S. lugdunensis
S. aureus
S. argenteus
S. carnosus
S. pseudintermedius
M. caseolyticus
M. canis
P. faecalis
Planococcus sp. PAMC 21323
P. antarcticus
P. glaciei
Kurthia sp. 11kri321
S. silvestris
L. seeligeri
L. ivanovii
L. welshimeri
L. monocytogenes
B. cereus
B. anthracis
B. thuringiensis
B. megaterium
B. subtilis
B. amyloliquefaciens
B. paralicheniformis
G. thermodenitrificans
Geobacillus genomosp. 3
G. kaustophilus
P. thermoglucosidasius
A. flavithermus
B. coagulans
H. halophilus
B. pseudofirmus
B. halodurans
B. selenitireducens
S. terrae
T. vulgaris
L. sacchari
Desmospora sp. 8437
P. terrae
P. polymyxa
P. larvae
K. tusciae
A. acidocaldarius
T. avium
N = 8
N = 10

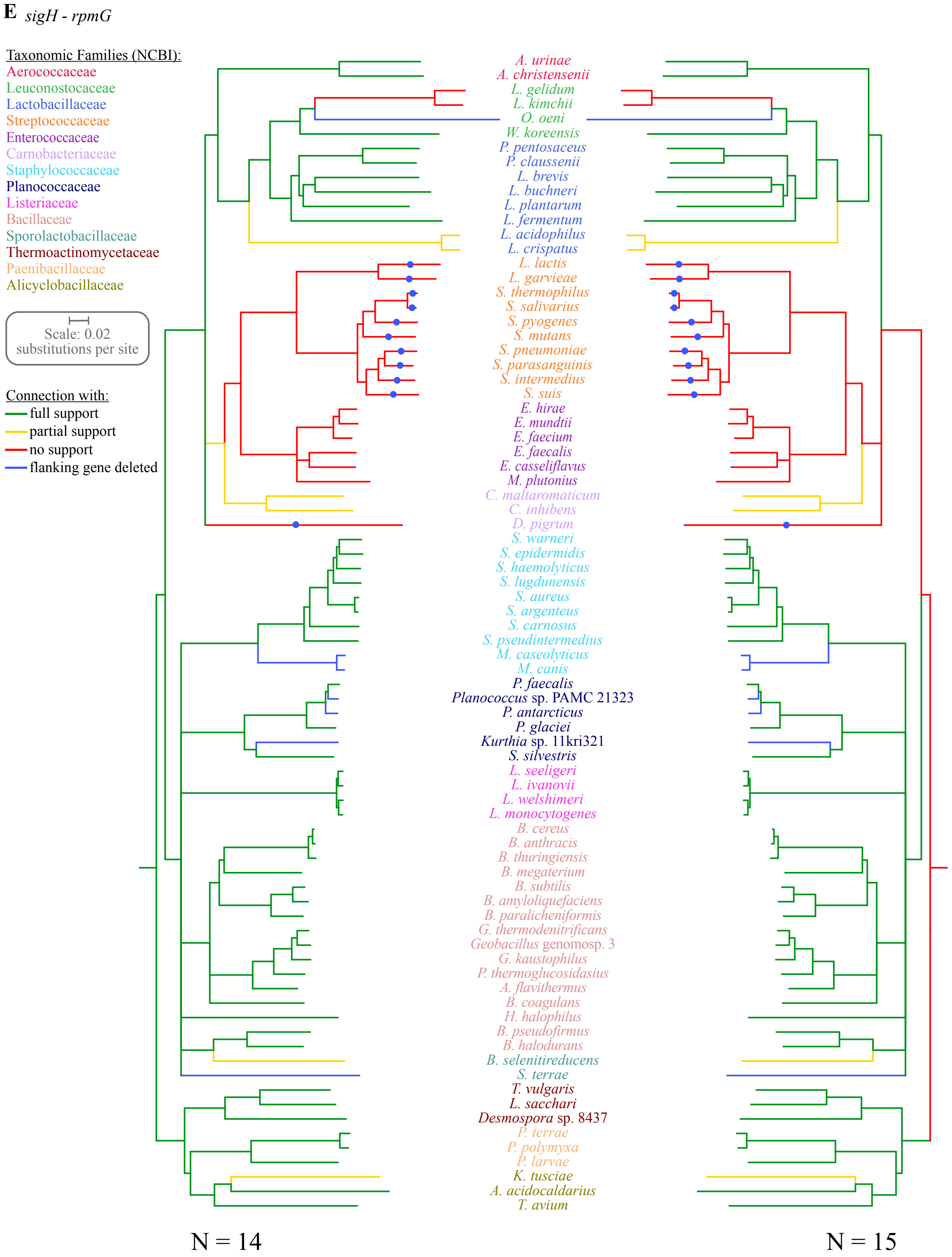
E sigH - rpmG
Taxonomic Families (NCBI):
Aerococcaceae
Leuconostocaceae
Lactobacillaceae
Streptococcaceae
Enterococcaceae
Carnobacteriaceae
Staphylococcaceae
Planococcaceae
Listeriaceae
Bacillaceae
Sporolactobacillaceae
Thermoactinomycetaceae
Paenibacillaceae
Alicyclobacillaceae
Scale: 0.02 substitutions per site
Connection with:
full support
partial support
no support
flanking gene deleted
A. urinae
A. christensenii
L. gelidum
L. kimchii
O. oeni
W. koreensis
P. pentosaceus
P. claussenii
L. brevis
L. buchneri
L. plantarum
L. fermentum
L. acidophilus
L. crispatus
L. lactis
L. garvieae
S. thermophilus
S. salivarius
S. pyogenes
S. mutans
S. pneumoniae
S. parasanguinis
S. intermedius
S. suis
E. hirae
E. mundtii
E. faecium
E. faecalis
E. casseliflavus
M. plutonius
C. maltaromaticum
C. inhibens
D. pigrum
S. warneri
S. epidermidis
S. haemolyticus
S. lugdunensis
S. aureus
S. argenteus
S. carnosus
S. pseudintermedius
M. caseolyticus
M. canis
P. faecalis
Planococcus sp. PAMC 21323
P. antarcticus
P. glaciei
Kurthia sp. 11kri321
S. silvestris
L. seeligeri
L. ivanovii
L. welshimeri
L. monocytogenes
B. cereus
B. anthracis
B. thuringiensis
B. megaterium
B. subtilis
B. amyloliquefaciens
B. paralicheniformis
G. thermodenitrificans
Geobacillus genomosp. 3
G. kaustophilus
P. thermoglucosidasius
A. flavithermus
B. coagulans
H. halophilus
B. pseudofirmus
B. halodurans
B. selenitireducens
S. terrae
T. vulgaris
L. sacchari
Desmospora sp. 8437
P. terrae
P. polymyxa
P. larvae
K. tusciae
A. acidocaldarius
T. avium
N = 14
N = 15

F *rpmG - secE*

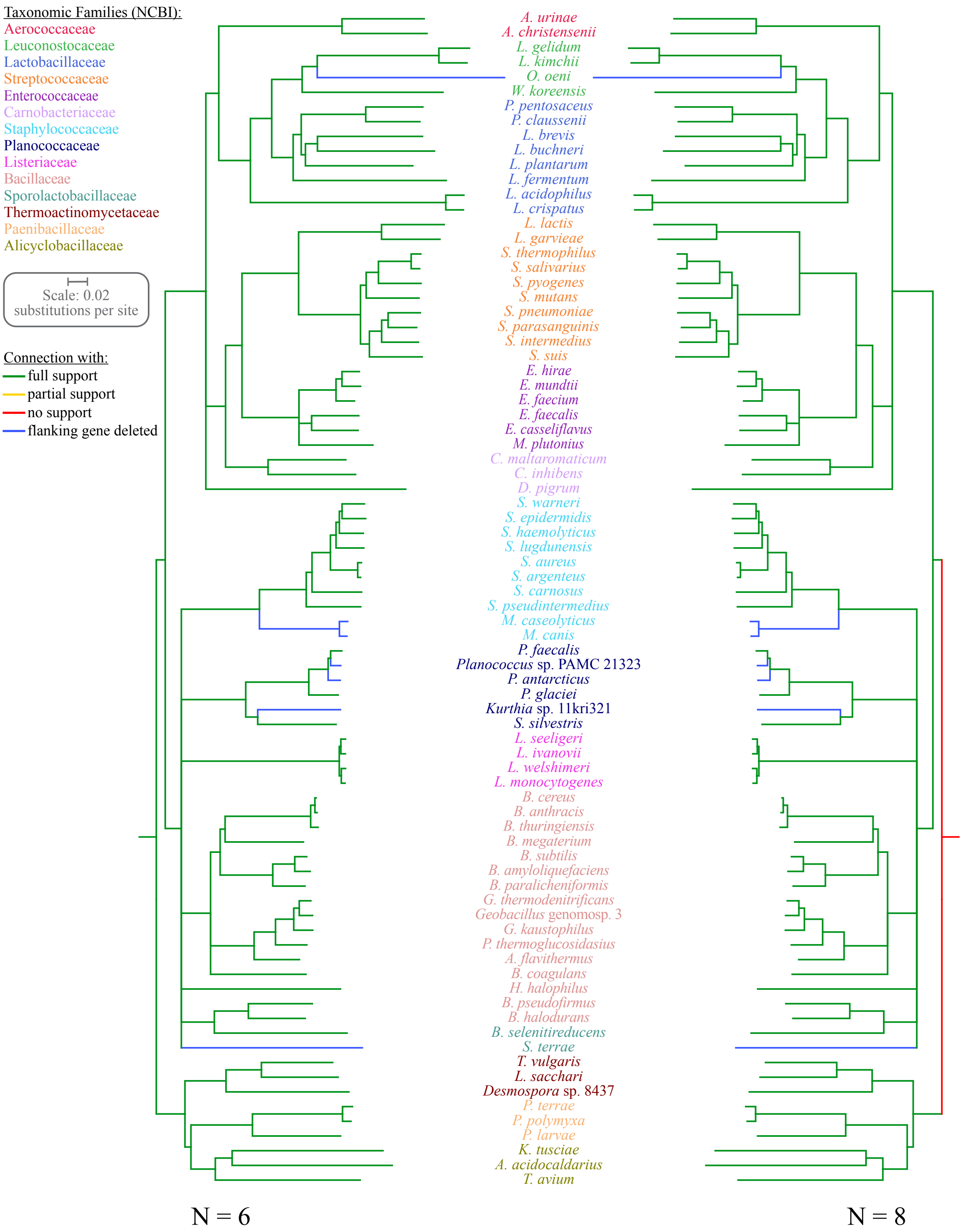

Taxonomic Families (NCBI):
Aerococcaceae
Leuconostocaceae
Lactobacillaceae
Streptococcaceae
Enterococcaceae
Carnobacteriaceae
Staphylococcaceae
Planococcaceae
Listeriaceae
Bacillaceae
Sporolactobacillaceae
Thermoactinomycetaceae
Paenibacillaceae
Alicyclobacillaceae
Scale: 0.02 substitutions per site
Connection with:
full support
partial support
no support
flanking gene deleted
A. urinae
A. christensenii
L. gelidum
L. kimchii
O. oeni
W. koreensis
P. pentosaceus
P. claussenii
L. brevis
L. buchneri
L. plantarum
L. fermentum
L. acidophilus
L. crispatus
L. lactis
L. garvieae
S. thermophilus
S. salivarius
S. pyogenes
S. mutans
S. pneumoniae
S. parasanguinis
S. intermedius
S. suis
E. hirae
E. mundtii
E. faecium
E. faecalis
E. casseliflavus
M. plutonius
C. maltaromaticum
C. inhibens
D. pigrum
S. warneri
S. epidermidis
S. haemolyticus
S. lugdunensis
S. aureus
S. argenteus
S. carnosus
S. pseudintermedius
M. caseolyticus
M. canis
P. faecalis
Planococcus sp. PAMC 21323
P. antarcticus
P. glaciei
Kurthia sp. 11kri321
S. silvestris
L. seeligeri
L. ivanovii
L. welshimeri
L. monocytogenes
B. cereus
B. anthracis
B. thuringiensis
B. megaterium
B. subtilis
B. amyloliquefaciens
B. paralicheniformis
G. thermodenitrificans
Geobacillus genomosp. 3
G. kaustophilus
P. thermoglucosidasius
A. flavithermus
B. coagulans
H. halophilus
B. pseudofirmus
B. halodurans
B. selenitireducens
S. terrae
T. vulgaris
L. sacchari
Desmospora sp. 8437
P. terrae
P. polymyxa
P. larvae
K. tusciae
A. acidocaldarius
T. avium
N = 6
N = 8

G *secE - nusG*

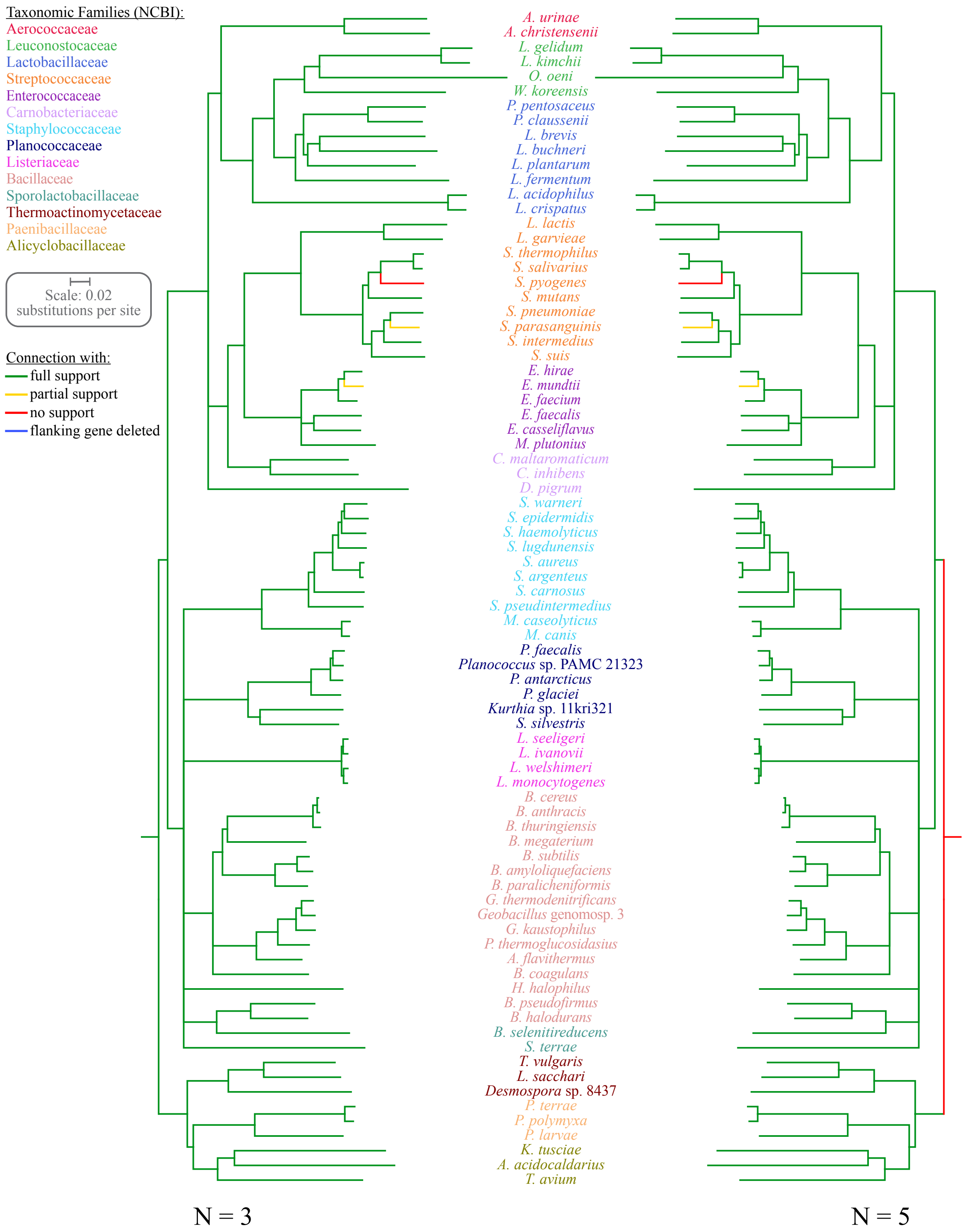
Taxonomic Families (NCBI):
Aerococcaceae
Leuconostocaceae
Lactobacillaceae
Streptococcaceae
Enterococcaceae
Carnobacteriaceae
Staphylococcaceae
Planococcaceae
Listeriaceae
Bacillaceae
Sporolactobacillaceae
Thermoactinomycetaceae
Paenibacillaceae
Alicyclobacillaceae
Scale: 0.02 substitutions per site
Connection with:
full support
partial support
no support
flanking gene deleted
A. urinae
A. christensenii
L. gelidum
L. kimchii
O. oeni
W. koreensis
P. pentosaceus
P. claussenii
L. brevis
L. buchneri
L. plantarum
L. fermentum
L. acidophilus
L. crispatus
L. lactis
L. garvieae
S. thermophilus
S. salivarius
S. pyogenes
S. mutans
S. pneumoniae
S. parasanguinis
S. intermedius
S. suis
E. hirae
E. mundtii
E. faecium
E. faecalis
E. casseliflavus
M. plutonius
C. maltaromaticum
C. inhibens
D. pigrum
S. warneri
S. epidermidis
S. haemolyticus
S. lugdunensis
S. aureus
S. argenteus
S. carnosus
S. pseudintermedius
M. caseolyticus
M. canis
P. faecalis
Planococcus sp. PAMC 21323
P. antarcticus
P. glaciei
Kurthia sp. 11kri321
S. silvestris
L. seeligeri
L. ivanovii
L. welshimeri
L. monocytogenes
B. cereus
B. anthracis
B. thuringiensis
B. megaterium
B. subtilis
B. amyloliquefaciens
B. paralicheniformis
G. thermodenitrificans
Geobacillus genomosp. 3
G. kaustophilus
P. thermoglucosidasius
A. flavithermus
B. coagulans
H. halophilus
B. pseudofirmus
B. halodurans
B. selenitireducens
S. terrae
T. vulgaris
L. sacchari
Desmospora sp. 8437
P. terrae
P. polymyxa
P. larvae
K. tusciae
A. acidocaldarius
T. avium
N = 3
N = 5

H *nusG - rplK*

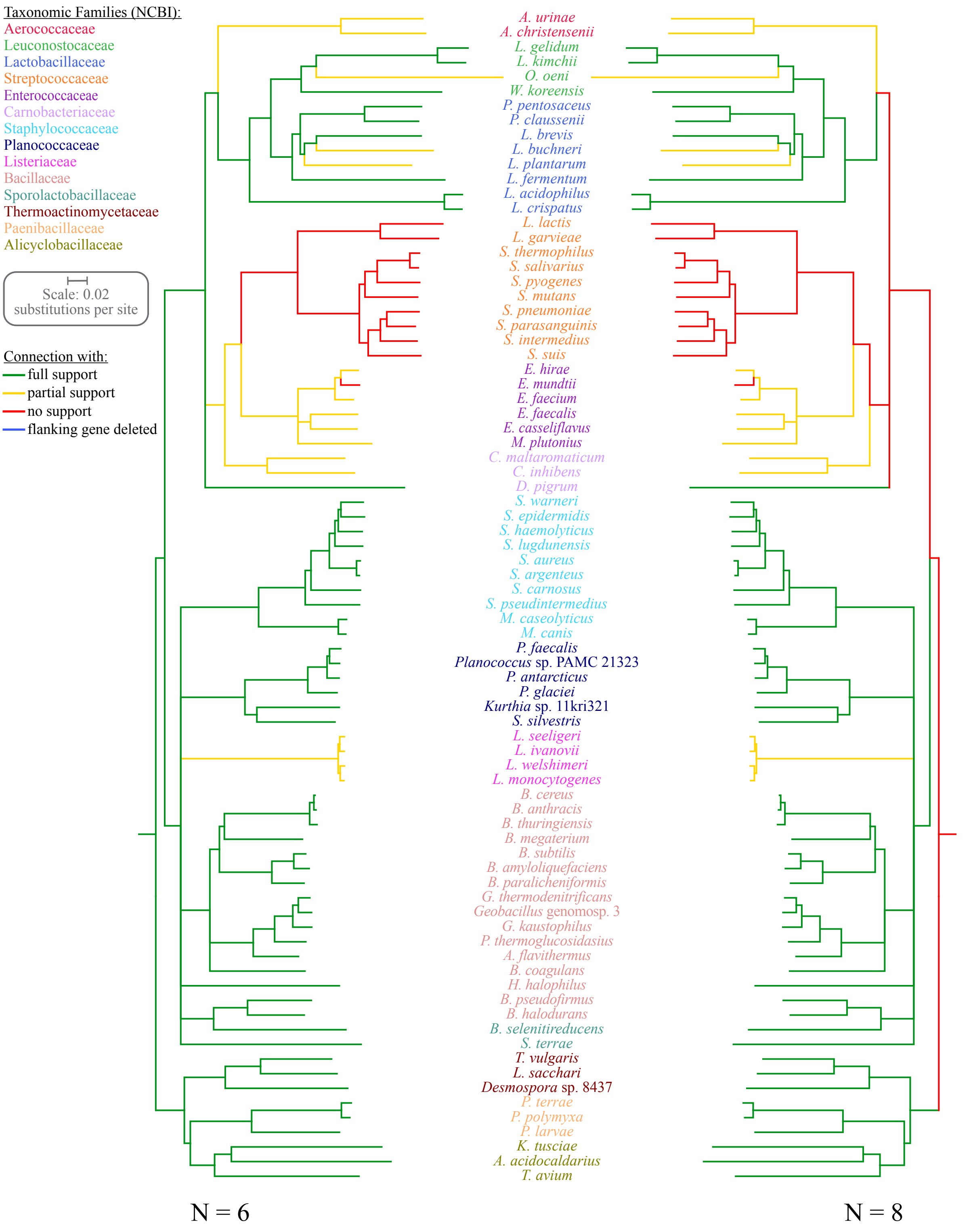
Taxonomic Families (NCBI):
Aerococcaceae
Leuconostocaceae
Lactobacillaceae
Streptococcaceae
Enterococcaceae
Carnobacteriaceae
Staphylococcaceae
Planococcaceae
Listeriaceae
Bacillaceae
Sporolactobacillaceae
Thermoactinomycetaceae
Paenibacillaceae
Alicyclobacillaceae
Scale: 0.02 substitutions per site
Connection with:
full support
partial support
no support
flanking gene deleted
A. urinae
A. christensenii
L. gelidum
L. kimchii
O. oeni
W. koreensis
P. pentosaceus
P. claussenii
L. brevis
L. buchneri
L. plantarum
L. fermentum
L. acidophilus
L. crispatus
L. lactis
L. garvieae
S. thermophilus
S. salivarius
S. pyogenes
S. mutans
S. pneumoniae
S. parasanguinis
S. intermedius
S. suis
E. hirae
E. mundtii
E. faecium
E. faecalis
E. casseliflavus
M. plutonius
C. maltaromaticum
C. inhibens
D. pigrum
S. warneri
S. epidermidis
S. haemolyticus
S. lugdunensis
S. aureus
S. argenteus
S. carnosus
S. pseudintermedius
M. caseolyticus
M. canis
P. faecalis
Planococcus sp. PAMC 21323
P. antarcticus
P. glaciei
Kurthia sp. 11kri321
S. silvestris
L. seeligeri
L. ivanovii
L. welshimeri
L. monocytogenes
B. cereus
B. anthracis
B. thuringiensis
B. megaterium
B. subtilis
B. amyloliquefaciens
B. paralicheniformis
G. thermodenitrificans
Geobacillus genomosp. 3
G. kaustophilus
P. thermoglucosidasius
A. flavithermus
B. coagulans
H. halophilus
B. pseudofirmus
B. halodurans
B. selenitireducens
S. terrae
T. vulgaris
L. sacchari
Desmospora sp. 8437
P. terrae
P. polymyxa
P. larvae
K. tusciae
A. acidocaldarius
T. avium
N = 6
N = 8

I *rplA - rplJ*

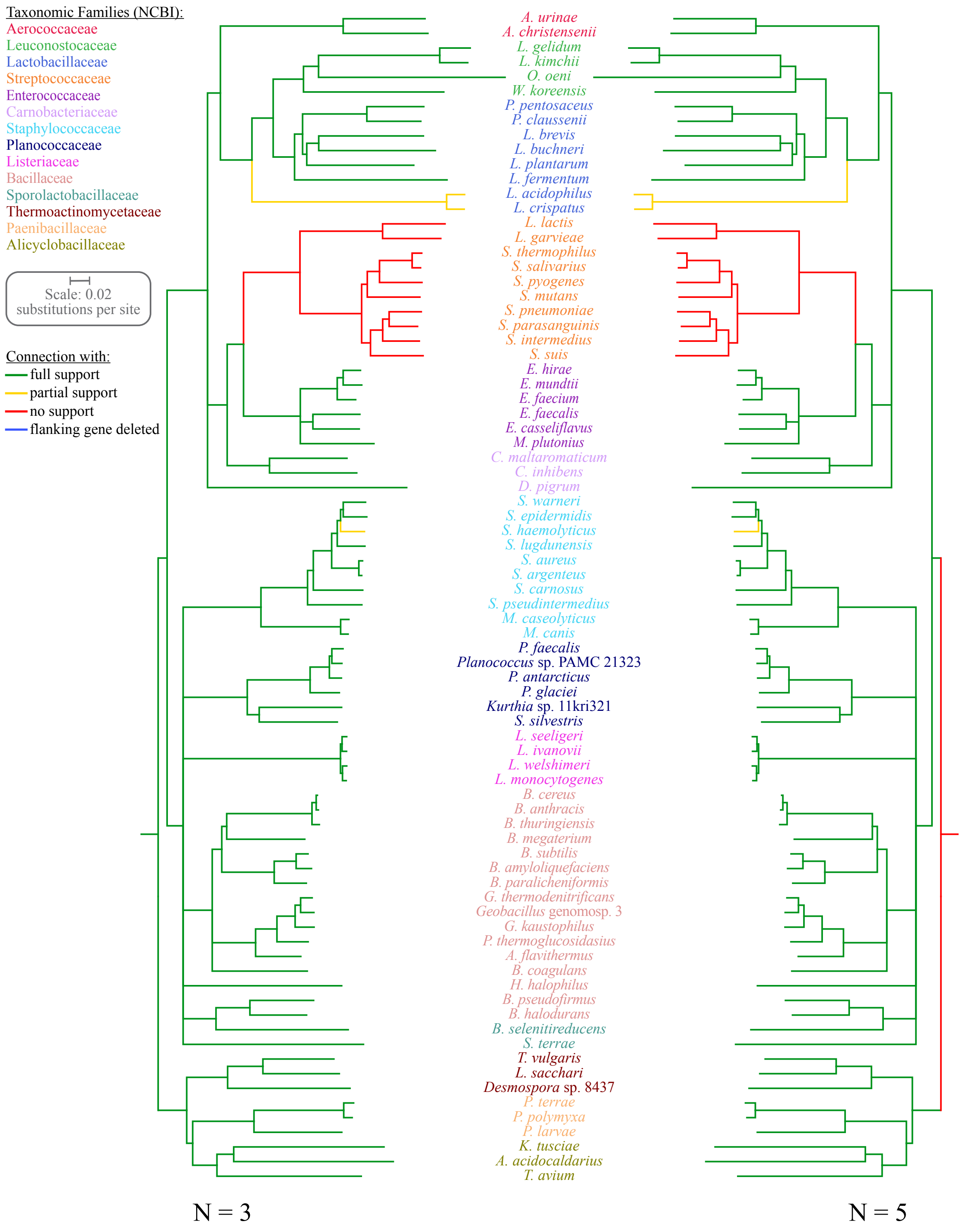

Taxonomic Families (NCBI):
Aerococcaceae
Leuconostocaceae
Lactobacillaceae
Streptococcaceae
Enterococcaceae
Carnobacteriaceae
Staphylococcaceae
Planococcaceae
Listeriaceae
Bacillaceae
Sporolactobacillaceae
Thermoactinomycetaceae
Paenibacillaceae
Alicyclobacillaceae
Scale: 0.02 substitutions per site
Connection with:
full support
partial support
no support
flanking gene deleted
A. urinae
A. christensenii
L. gelidum
L. kimchii
O. oeni
W. koreensis
P. pentosaceus
P. claussenii
L. brevis
L. buchneri
L. plantarum
L. fermentum
L. acidophilus
L. crispatus
L. lactis
L. garvieae
S. thermophilus
S. salivarius
S. pyogenes
S. mutans
S. pneumoniae
S. parasanguinis
S. intermedius
S. suis
E. hirae
E. mundtii
E. faecium
E. faecalis
E. casseliflavus
M. plutonius
C. maltaromaticum
C. inhibens
D. pigrum
S. warneri
S. epidermidis
S. haemolyticus
S. lugdunensis
S. aureus
S. argenteus
S. carnosus
S. pseudintermedius
M. caseolyticus
M. canis
P. faecalis
Planococcus sp. PAMC 21323
P. antarcticus
P. glaciei
Kurthia sp. 11kri321
S. silvestris
L. seeligeri
L. ivanovii
L. welshimeri
L. monocytogenes
B. cereus
B. anthracis
B. thuringiensis
B. megaterium
B. subtilis
B. amyloliquefaciens
B. paralicheniformis
G. thermodenitrificans
Geobacillus genomosp. 3
G. kaustophilus
P. thermoglucosidasius
A. flavithermus
B. coagulans
H. halophilus
B. pseudofirmus
B. halodurans
B. selenitireducens
S. terrae
T. vulgaris
L. sacchari
Desmospora sp. 8437
P. terrae
P. polymyxa
P. larvae
K. tusciae
A. acidocaldarius
T. avium
N = 3
N = 5

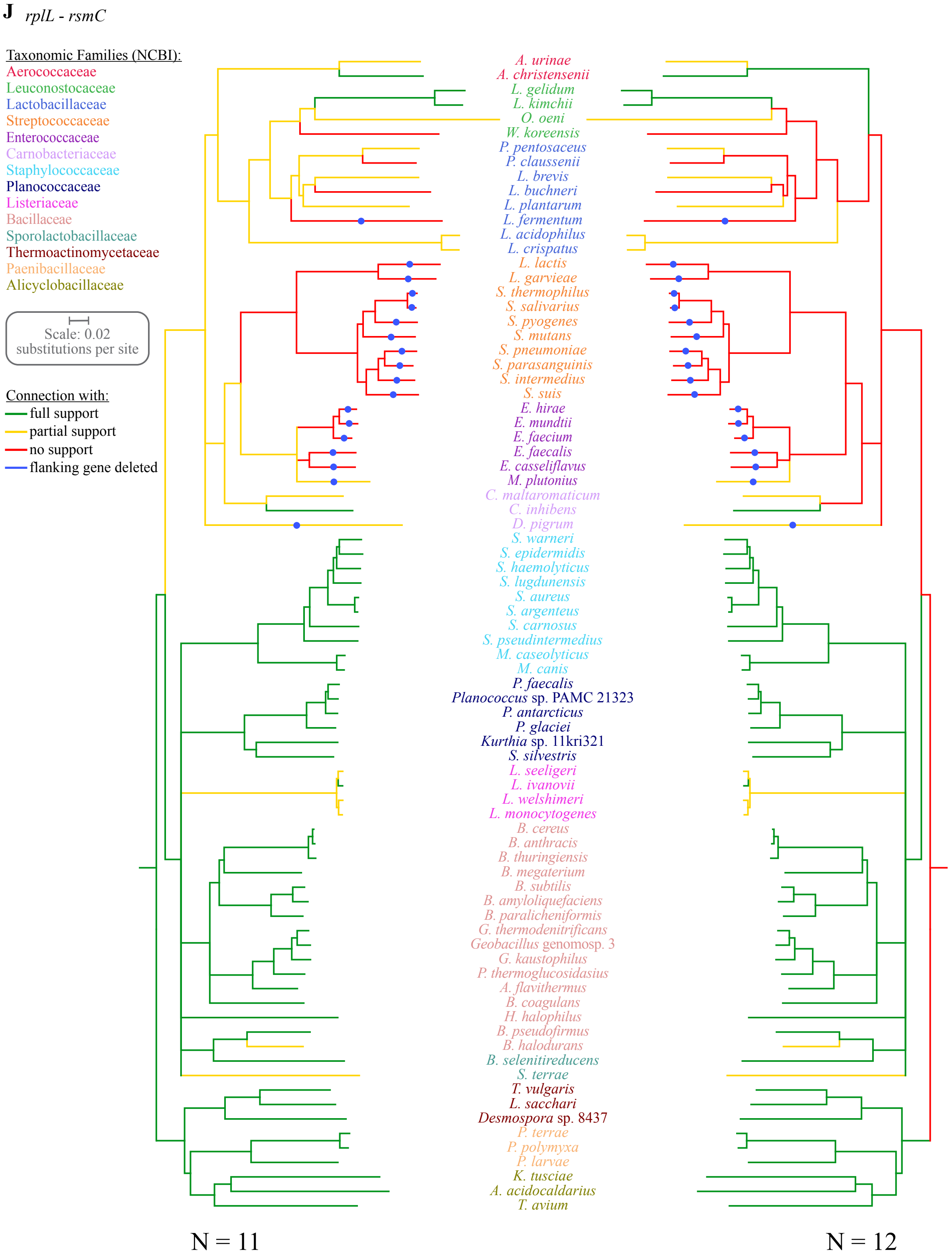
J rplL - rsmC
Taxonomic Families (NCBI):
Aerococcaceae
Leuconostocaceae
Lactobacillaceae
Streptococcaceae
Enterococcaceae
Carnobacteriaceae
Staphylococcaceae
Planococcaceae
Listeriaceae
Bacillaceae
Sporolactobacillaceae
Thermoactinomycetaceae
Paenibacillaceae
Alicyclobacillaceae
Scale: 0.02
substitutions per site
Connection with:
full support
partial support
no support
flanking gene deleted
A. urinae
A. christensenii
L. gelidum
L. kimchii
O. oeni
W. koreensis
P. pentosaceus
P. claussenii
L. brevis
L. buchneri
L. plantarum
L. fermentum
L. acidophilus
L. crispatus
L. lactis
L. garvieae
S. thermophilus
S. salivarius
S. pyogenes
S. mutans
S. pneumoniae
S. parasanguinis
S. intermedius
S. suis
E. hirae
E. mundtii
E. faecium
E. faecalis
E. casseliflavus
M. plutonius
C. maltaromaticum
C. inhibens
D. pigrum
S. warneri
S. epidermidis
S. haemolyticus
S. lugdunensis
S. aureus
S. argenteus
S. carnosus
S. pseudintermedius
M. caseolyticus
M. canis
P. faecalis
Planococcus sp. PAMC 21323
P. antarcticus
P. glaciei
Kurthia sp. 11kri321
S. silvestris
L. seeligeri
L. ivanovii
L. welshimeri
L. monocytogenes
B. cereus
B. anthracis
B. thuringiensis
B. megaterium
B. subtilis
B. amyloliquefaciens
B. paralicheniformis
G. thermodenitrificans
Geobacillus genomosp. 3
G. kaustophilus
P. thermoglucosidasius
A. flavithermus
B. coagulans
H. halophilus
B. pseudofirmus
B. halodurans
B. selenitireducens
S. terrae
T. vulgaris
L. sacchari
Desmospora sp. 8437
P. terrae
P. polymyxa
P. larvae
K. tusciae
A. acidocaldarius
T. avium
N = 11
N = 12

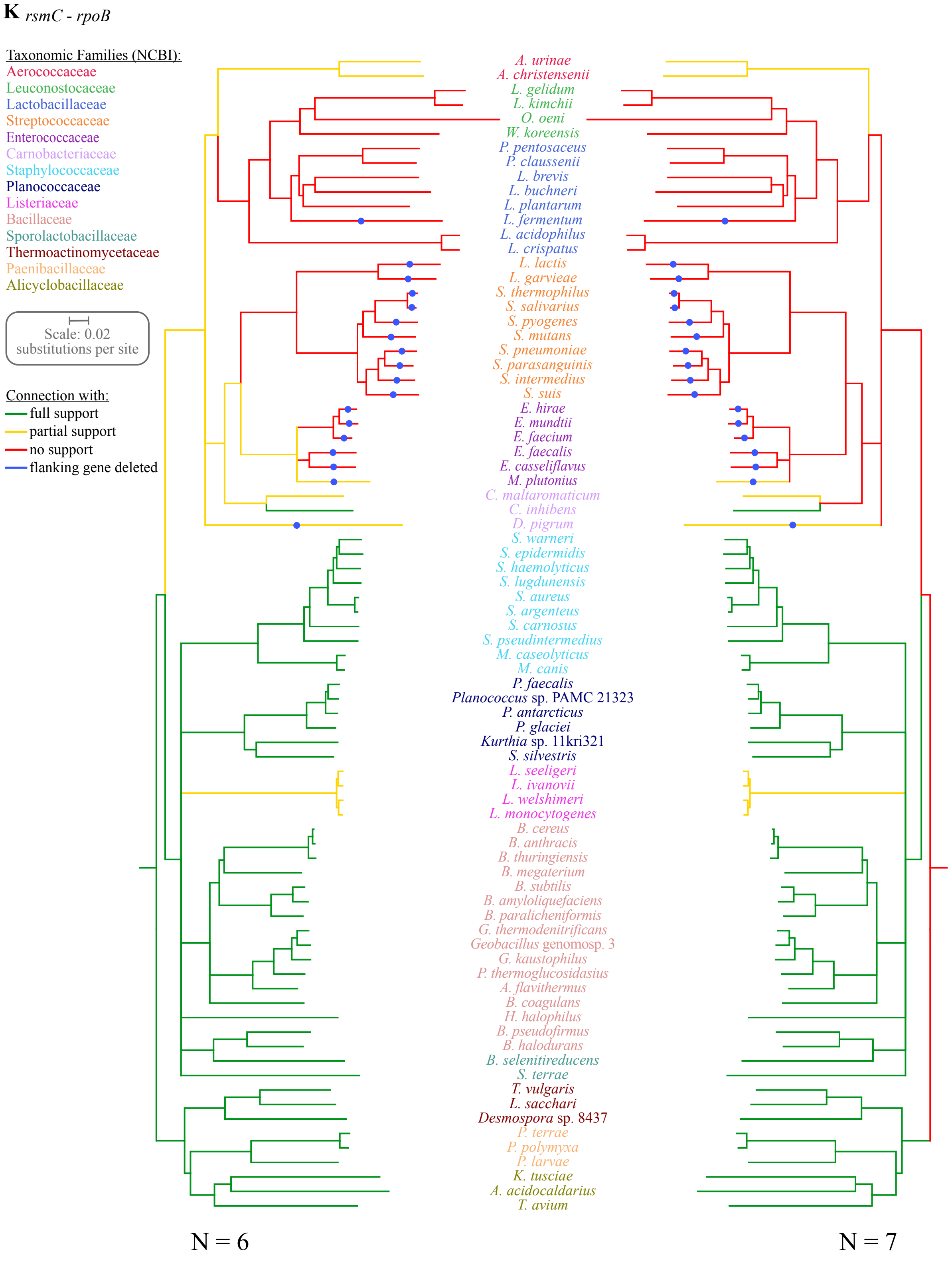
K rsmC - rpoB
Taxonomic Families (NCBI):
Aerococcaceae
Leuconostocaceae
Lactobacillaceae
Streptococcaceae
Enterococcaceae
Carnobacteriaceae
Staphylococcaceae
Planococcaceae
Listeriaceae
Bacillaceae
Sporolactobacillaceae
Thermoactinomycetaceae
Paenibacillaceae
Alicyclobacillaceae
Scale: 0.02
substitutions per site
Connection with:
full support
partial support
no support
flanking gene deleted
A. urinae
A. christensenii
L. gelidum
L. kimchii
O. oeni
W. koreensis
P. pentosaceus
P. claussenii
L. brevis
L. buchneri
L. plantarum
L. fermentum
L. acidophilus
L. crispatus
L. lactis
L. garvieae
S. thermophilus
S. salivarius
S. pyogenes
S. mutans
S. pneumoniae
S. parasanguinis
S. intermedius
S. suis
E. hirae
E. mundtii
E. faecium
E. faecalis
E. casseliflavus
M. plutonius
C. maltaromaticum
C. inhibens
D. pigrum
S. warneri
S. epidermidis
S. haemolyticus
S. lugdunensis
S. aureus
S. argenteus
S. carnosus
S. pseudintermedius
M. caseolyticus
M. canis
P. faecalis
Planococcus sp. PAMC 21323
P. antarcticus
P. glaciei
Kurthia sp. 11kri321
S. silvestris
L. seeligeri
L. ivanovii
L. welshimeri
L. monocytogenes
B. cereus
B. anthracis
B. thuringiensis
B. megaterium
B. subtilis
B. amyloliquefaciens
B. paralicheniformis
G. thermodenitrificans
Geobacillus genomosp. 3
G. kaustophilus
P. thermoglucosidasius
A. flavithermus
B. coagulans
H. halophilus
B. pseudofirmus
B. halodurans
B. selenitireducens
S. terrae
T. vulgaris
L. sacchari
Desmospora sp. 8437
P. terrae
P. polymyxa
P. larvae
K. tusciae
A. acidocaldarius
T. avium
N = 6
N = 7

L *rpoC - rpl7ae*

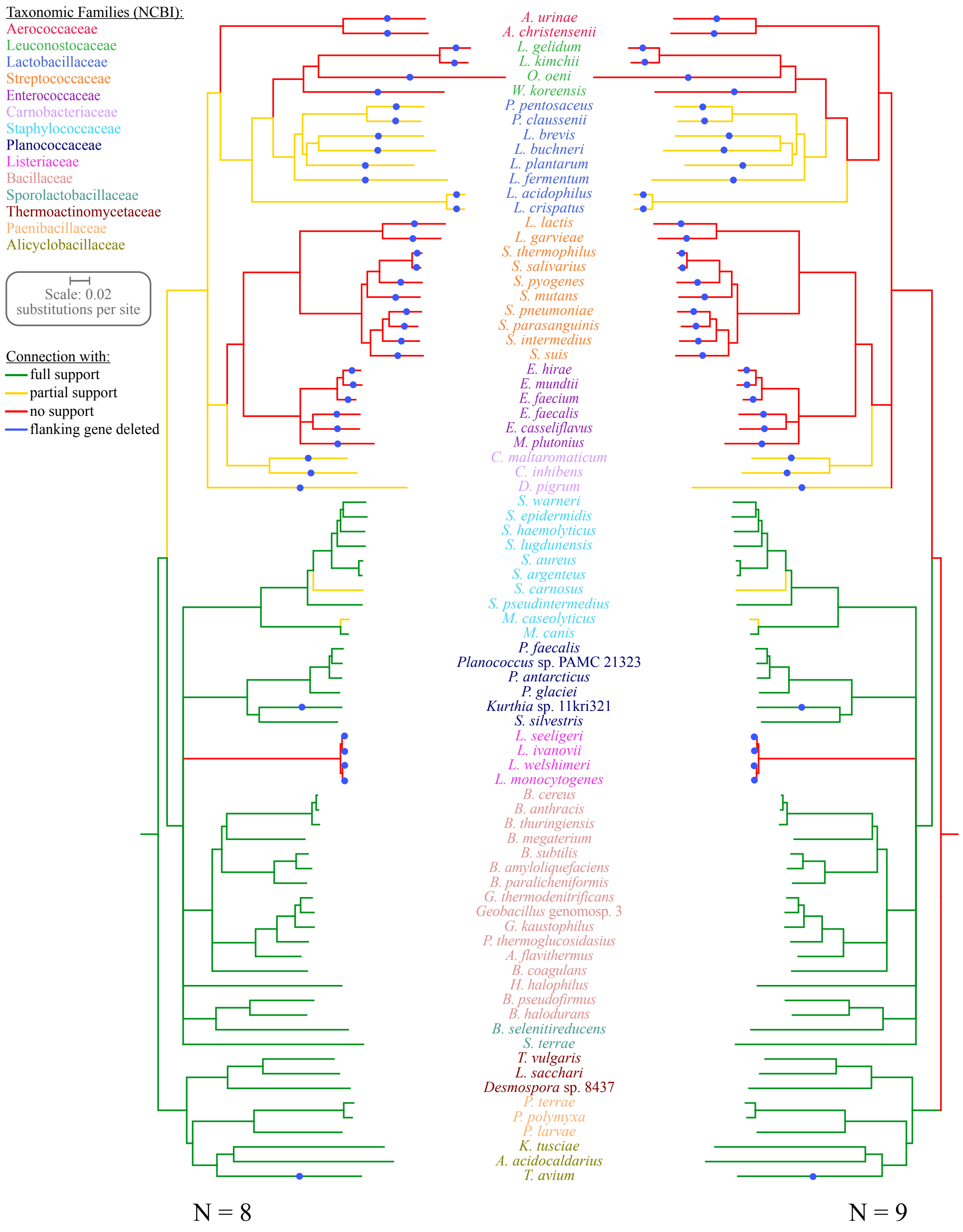

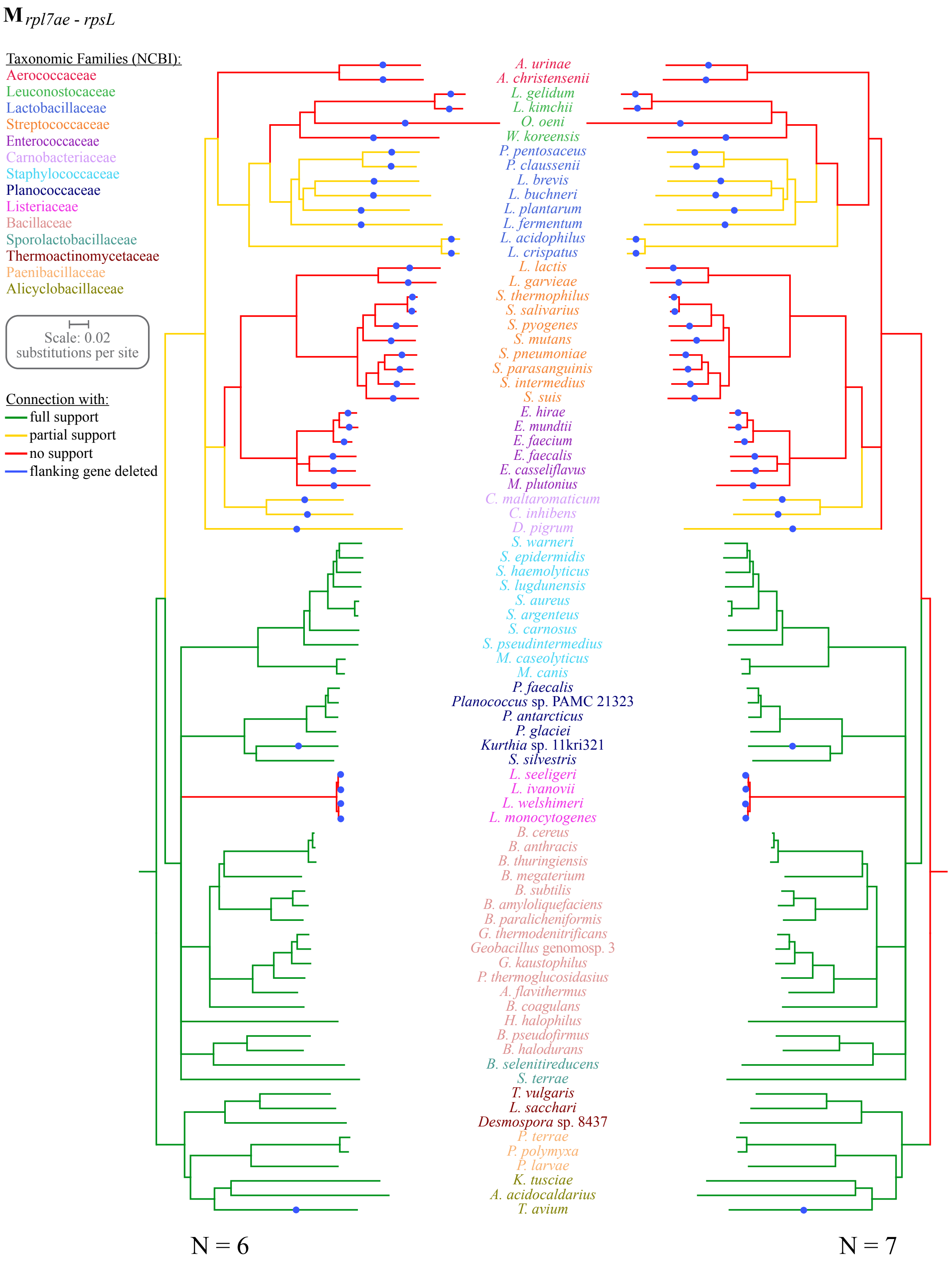
M rpl7ae - rpsL
Taxonomic Families (NCBI):
Aerococcaceae
Leuconostocaceae
Lactobacillaceae
Streptococcaceae
Enterococcaceae
Carnobacteriaceae
Staphylococcaceae
Planococcaceae
Listeriaceae
Bacillaceae
Sporolactobacillaceae
Thermoactinomycetaceae
Paenibacillaceae
Alicyclobacillaceae
Scale: 0.02
substitutions per site
Connection with:
full support
partial support
no support
flanking gene deleted
A. urinae
A. christensenii
L. gelidum
L. kimchii
O. oeni
W. koreensis
P. pentosaceus
P. claussenii
L. brevis
L. buchneri
L. plantarum
L. fermentum
L. acidophilus
L. crispatus
L. lactis
L. garvieae
S. thermophilus
S. salivarius
S. pyogenes
S. mutans
S. pneumoniae
S. parasanguinis
S. intermedius
S. suis
E. hirae
E. mundtii
E. faecium
E. faecalis
E. casseliflavus
M. plutonius
C. maltaromaticum
C. inhibens
D. pigrum
S. warneri
S. epidermidis
S. haemolyticus
S. lugdunensis
S. aureus
S. argenteus
S. carnosus
S. pseudintermedius
M. caseolyticus
M. canis
P. faecalis
Planococcus sp. PAMC 21323
P. antarcticus
P. glaciei
Kurthia sp. 11kri321
S. silvestris
L. seeligeri
L. ivanovii
L. welshimeri
L. monocytogenes
B. cereus
B. anthracis
B. thuringiensis
B. megaterium
B. subtilis
B. amyloliquefaciens
B. paralicheniformis
G. thermodenitrificans
Geobacillus genomosp. 3
G. kaustophilus
P. thermoglucosidasius
A. flavithermus
B. coagulans
H. halophilus
B. pseudofirmus
B. halodurans
B. selenitireducens
S. terrae
T. vulgaris
L. sacchari
Desmospora sp. 8437
P. terrae
P. polymyxa
P. larvae
K. tusciae
A. acidocaldarius
T. avium
N = 6
N = 7

N *rpsG - fusA*

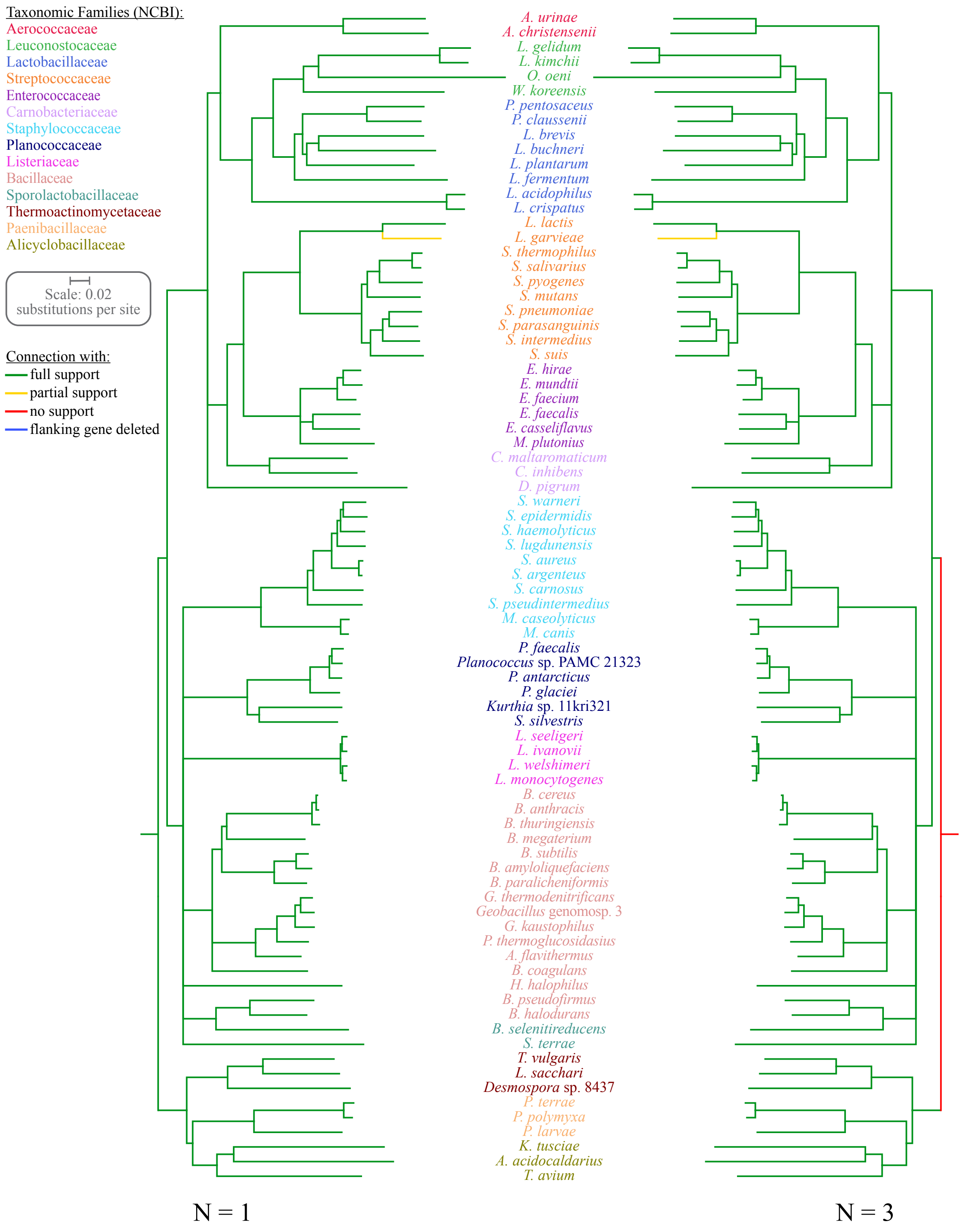
Taxonomic Families (NCBI):
Aerococcaceae
Leuconostocaceae
Lactobacillaceae
Streptococcaceae
Enterococcaceae
Carnobacteriaceae
Staphylococcaceae
Planococcaceae
Listeriaceae
Bacillaceae
Sporolactobacillaceae
Thermoactinomycetaceae
Paenibacillaceae
Alicyclobacillaceae
Scale: 0.02
substitutions per site
Connection with:
full support
partial support
no support
flanking gene deleted
A. urinae
A. christensenii
L. gelidum
L. kimchii
O. oeni
W. koreensis
P. pentosaceus
P. claussenii
L. brevis
L. buchneri
L. plantarum
L. fermentum
L. acidophilus
L. crispatus
L. lactis
L. garvieae
S. thermophilus
S. salivarius
S. pyogenes
S. mutans
S. pneumoniae
S. parasanguinis
S. intermedius
S. suis
E. hirae
E. mundtii
E. faecium
E. faecalis
E. casseliflavus
M. plutonius
C. maltaromaticum
C. inhibens
D. pigrum
S. warneri
S. epidermidis
S. haemolyticus
S. lugdunensis
S. aureus
S. argenteus
S. carnosus
S. pseudintermedius
M. caseolyticus
M. canis
P. faecalis
Planococcus sp. PAMC 21323
P. antarcticus
P. glaciei
Kurthia sp. 11kri321
S. silvestris
L. seeligeri
L. ivanovii
L. welshimeri
L. monocytogenes
B. cereus
B. anthracis
B. thuringiensis
B. megaterium
B. subtilis
B. amyloliquefaciens
B. paralicheniformis
G. thermodenitrificans
Geobacillus genomosp. 3
G. kaustophilus
P. thermoglucosidasius
A. flavithermus
B. coagulans
H. halophilus
B. pseudofirmus
B. halodurans
B. selenitireducens
S. terrae
T. vulgaris
L. sacchari
Desmospora sp. 8437
P. terrae
P. polymyxa
P. larvae
K. tusciae
A. acidocaldarius
T. avium
N = 1
N = 3

O *fusA - tufA*

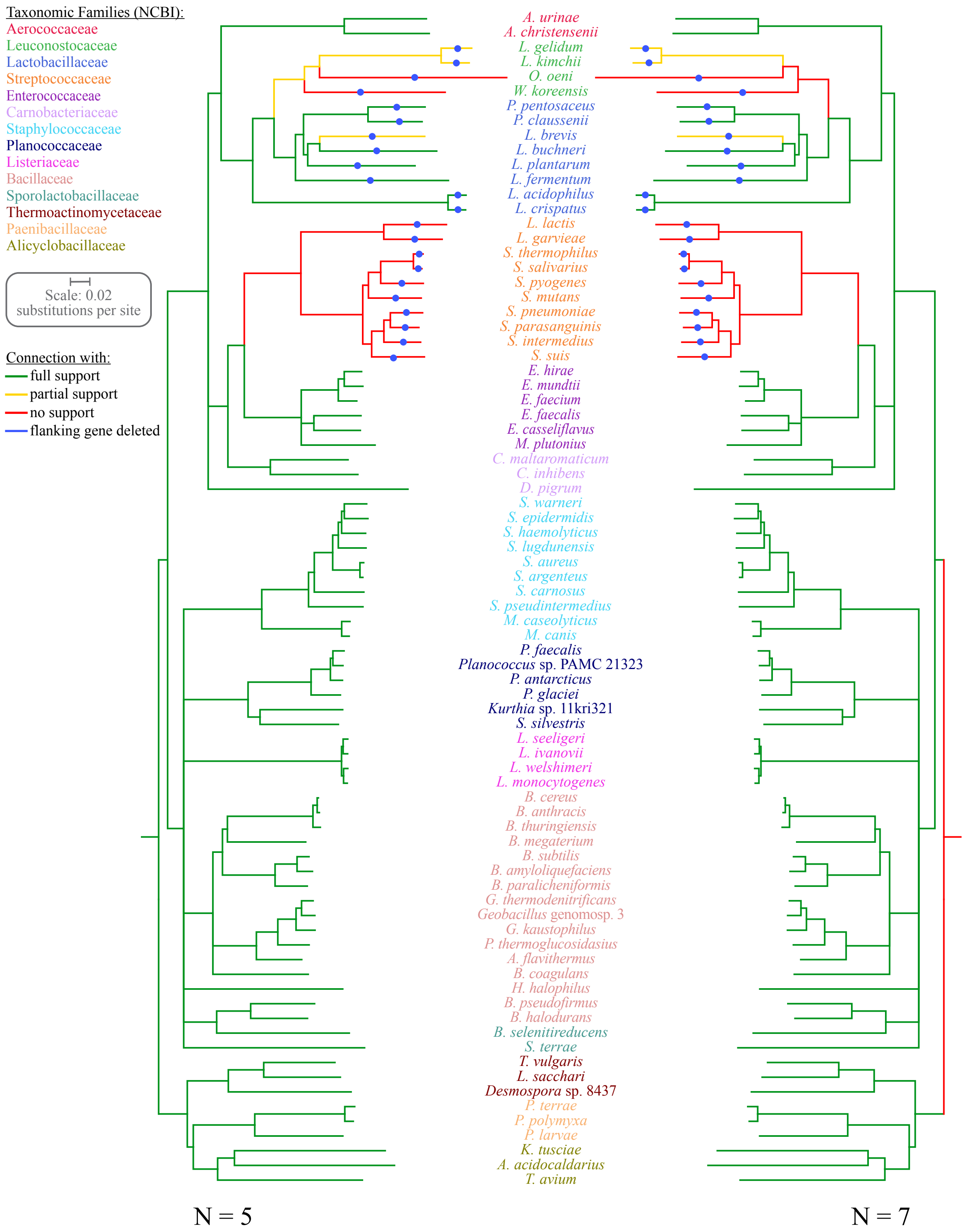

Taxonomic Families (NCBI):
Aerococcaceae
Leuconostocaceae
Lactobacillaceae
Streptococcaceae
Enterococcaceae
Carnobacteriaceae
Staphylococcaceae
Planococcaceae
Listeriaceae
Bacillaceae
Sporolactobacillaceae
Thermoactinomycetaceae
Paenibacillaceae
Alicyclobacillaceae
Scale: 0.02 substitutions per site
Connection with:
full support
partial support
no support
flanking gene deleted
A. urinae
A. christensenii
L. gelidum
L. kimchii
O. oeni
W. koreensis
P. pentosaceus
P. claussenii
L. brevis
L. buchneri
L. plantarum
L. fermentum
L. acidophilus
L. crispatus
L. lactis
L. garvieae
S. thermophilus
S. salivarius
S. pyogenes
S. mutans
S. pneumoniae
S. parasanguinis
S. intermedius
S. suis
E. hirae
E. mundtii
E. faecium
E. faecalis
E. casseliflavus
M. plutonius
C. maltaromaticum
C. inhibens
D. pigrum
S. warneri
S. epidermidis
S. haemolyticus
S. lugdunensis
S. aureus
S. argenteus
S. carnosus
S. pseudintermedius
M. caseolyticus
M. canis
P. faecalis
Planococcus sp. PAMC 21323
P. antarcticus
P. glaciei
Kurthia sp. 11kri321
S. silvestris
L. seeligeri
L. ivanovii
L. welshimeri
L. monocytogenes
B. cereus
B. anthracis
B. thuringiensis
B. megaterium
B. subtilis
B. amyloliquefaciens
B. paralicheniformis
G. thermodenitrificans
Geobacillus genomosp. 3
G. kaustophilus
P. thermoglucosidasius
A. flavithermus
B. coagulans
H. halophilus
B. pseudofirmus
B. halodurans
B. selenitireducens
S. terrae
T. vulgaris
L. sacchari
Desmospora sp. 8437
P. terrae
P. polymyxa
P. larvae
K. tusciae
A. acidocaldarius
T. avium
N = 5
N = 7

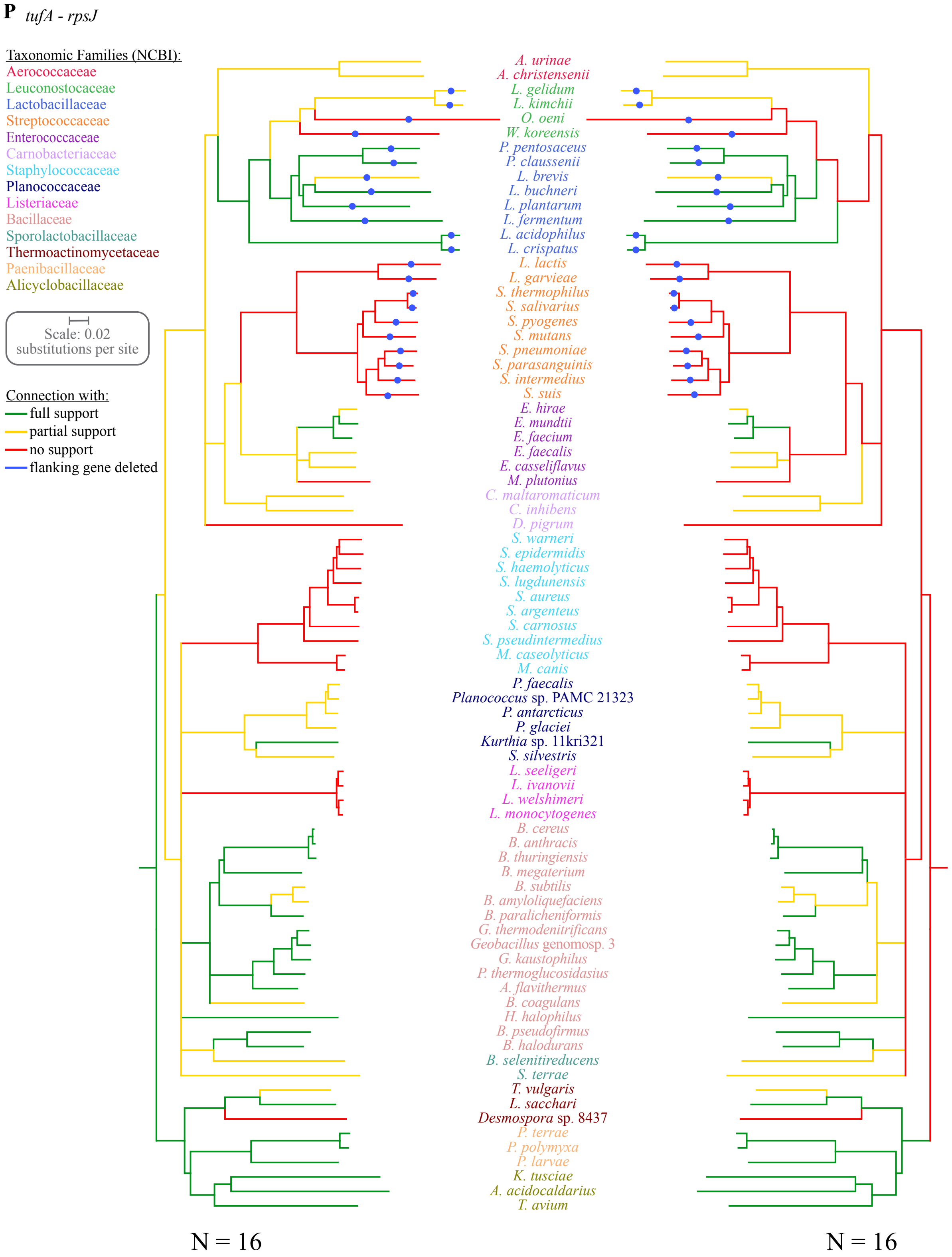
P
tufA - rpsJ
Taxonomic Families (NCBI):
Aerococcaceae
Leuconostocaceae
Lactobacillaceae
Streptococcaceae
Enterococcaceae
Carnobacteriaceae
Staphylococcaceae
Planococcaceae
Listeriaceae
Bacillaceae
Sporolactobacillaceae
Thermoactinomycetaceae
Paenibacillaceae
Alicyclobacillaceae
Scale: 0.02
substitutions per site
Connection with:
full support
partial support
no support
flanking gene deleted
A. urinae
A. christensenii
L. gelidum
L. kimchii
O. oeni
W. koreensis
P. pentosaceus
P. claussenii
L. brevis
L. buchneri
L. plantarum
L. fermentum
L. acidophilus
L. crispatus
L. lactis
L. garvieae
S. thermophilus
S. salivarius
S. pyogenes
S. mutans
S. pneumoniae
S. parasanguinis
S. intermedius
S. suis
E. hirae
E. mundtii
E. faecium
E. faecalis
E. casseliflavus
M. plutonius
C. maltaromaticum
C. inhibens
D. pigrum
S. warneri
S. epidermidis
S. haemolyticus
S. lugdunensis
S. aureus
S. argenteus
S. carnosus
S. pseudintermedius
M. caseolyticus
M. canis
P. faecalis
Planococcus sp. PAMC 21323
P. antarcticus
P. glaciei
Kurthia sp. 11kri321
S. silvestris
L. seeligeri
L. ivanovii
L. welshimeri
L. monocytogenes
B. cereus
B. anthracis
B. thuringiensis
B. megaterium
B. subtilis
B. amyloliquefaciens
B. paralicheniformis
G. thermodenitrificans
Geobacillus genomosp. 3
G. kaustophilus
P. thermoglucosidasius
A. flavithermus
B. coagulans
H. halophilus
B. pseudofirmus
B. halodurans
B. selenitireducens
S. terrae
T. vulgaris
L. sacchari
Desmospora sp. 8437
P. terrae
P. polymyxa
P. larvae
K. tusciae
A. acidocaldarius
T. avium
N = 16
N = 16

Q *rplE - rpsN*

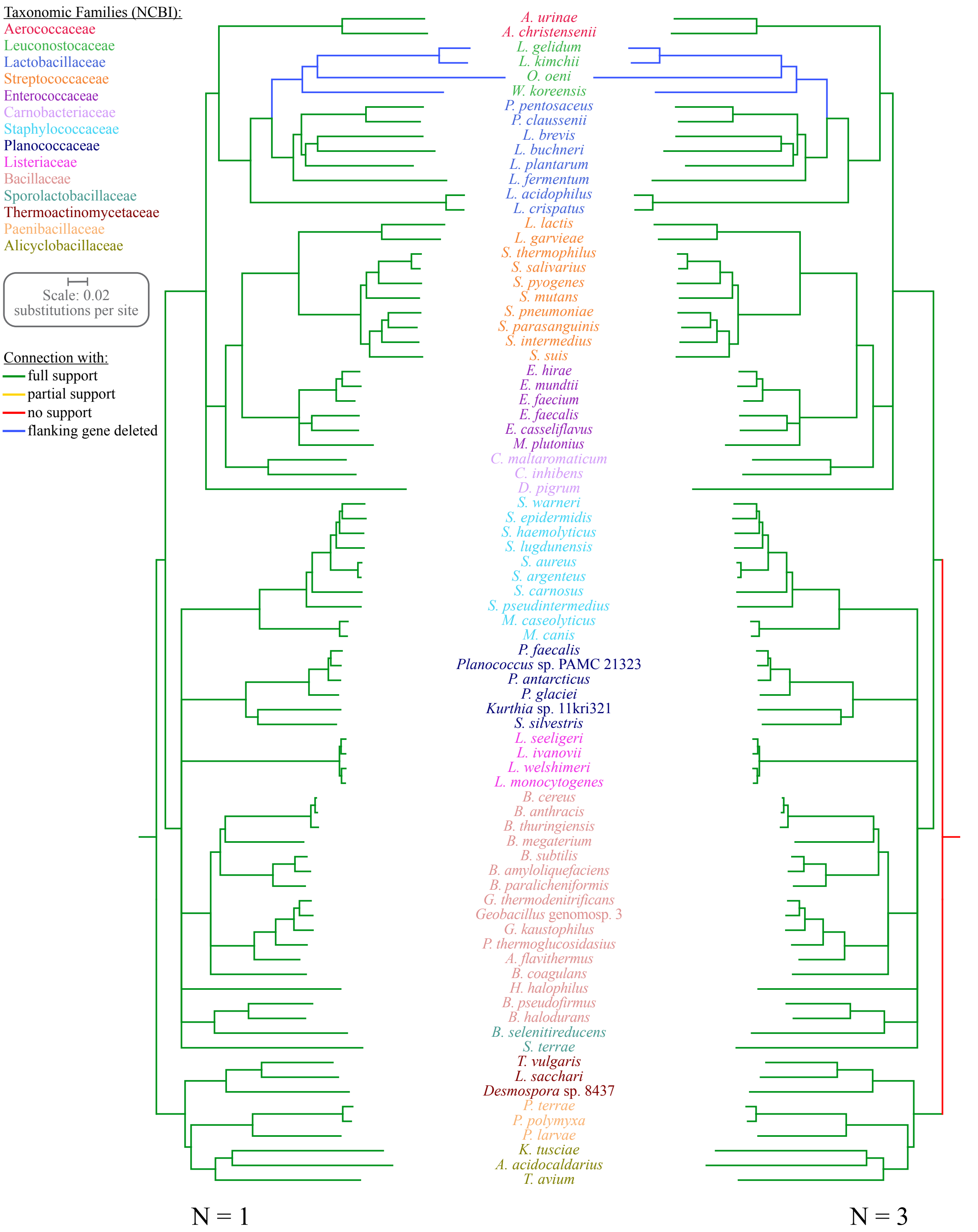

Taxonomic Families (NCBI):
Aerococcaceae
Leuconostocaceae
Lactobacillaceae
Streptococcaceae
Enterococcaceae
Carnobacteriaceae
Staphylococcaceae
Planococcaceae
Listeriaceae
Bacillaceae
Sporolactobacillaceae
Thermoactinomycetaceae
Paenibacillaceae
Alicyclobacillaceae
Scale: 0.02 substitutions per site
Connection with:
full support
partial support
no support
flanking gene deleted
A. urinae
A. christensenii
L. gelidum
L. kimchii
O. oeni
W. koreensis
P. pentosaceus
P. claussenii
L. brevis
L. buchneri
L. plantarum
L. fermentum
L. acidophilus
L. crispatus
L. lactis
L. garvieae
S. thermophilus
S. salivarius
S. pyogenes
S. mutans
S. pneumoniae
S. parasanguinis
S. intermedius
S. suis
E. hirae
E. mundtii
E. faecium
E. faecalis
E. casseliflavus
M. plutonius
C. maltaromaticum
C. inhibens
D. pigrum
S. warneri
S. epidermidis
S. haemolyticus
S. lugdunensis
S. aureus
S. argenteus
S. carnosus
S. pseudintermedius
M. caseolyticus
M. canis
P. faecalis
Planococcus sp. PAMC 21323
P. antarcticus
P. glaciei
Kurthia sp. 11kri321
S. silvestris
L. seeligeri
L. ivanovii
L. welshimeri
L. monocytogenes
B. cereus
B. anthracis
B. thuringiensis
B. megaterium
B. subtilis
B. amyloliquefaciens
B. paralicheniformis
G. thermodenitrificans
Geobacillus genomosp. 3
G. kaustophilus
P. thermoglucosidasius
A. flavithermus
B. coagulans
H. halophilus
B. pseudofirmus
B. halodurans
B. selenitireducens
S. terrae
T. vulgaris
L. sacchari
Desmospora sp. 8437
P. terrae
P. polymyxa
P. larvae
K. tusciae
A. acidocaldarius
T. avium
N = 1
N = 3

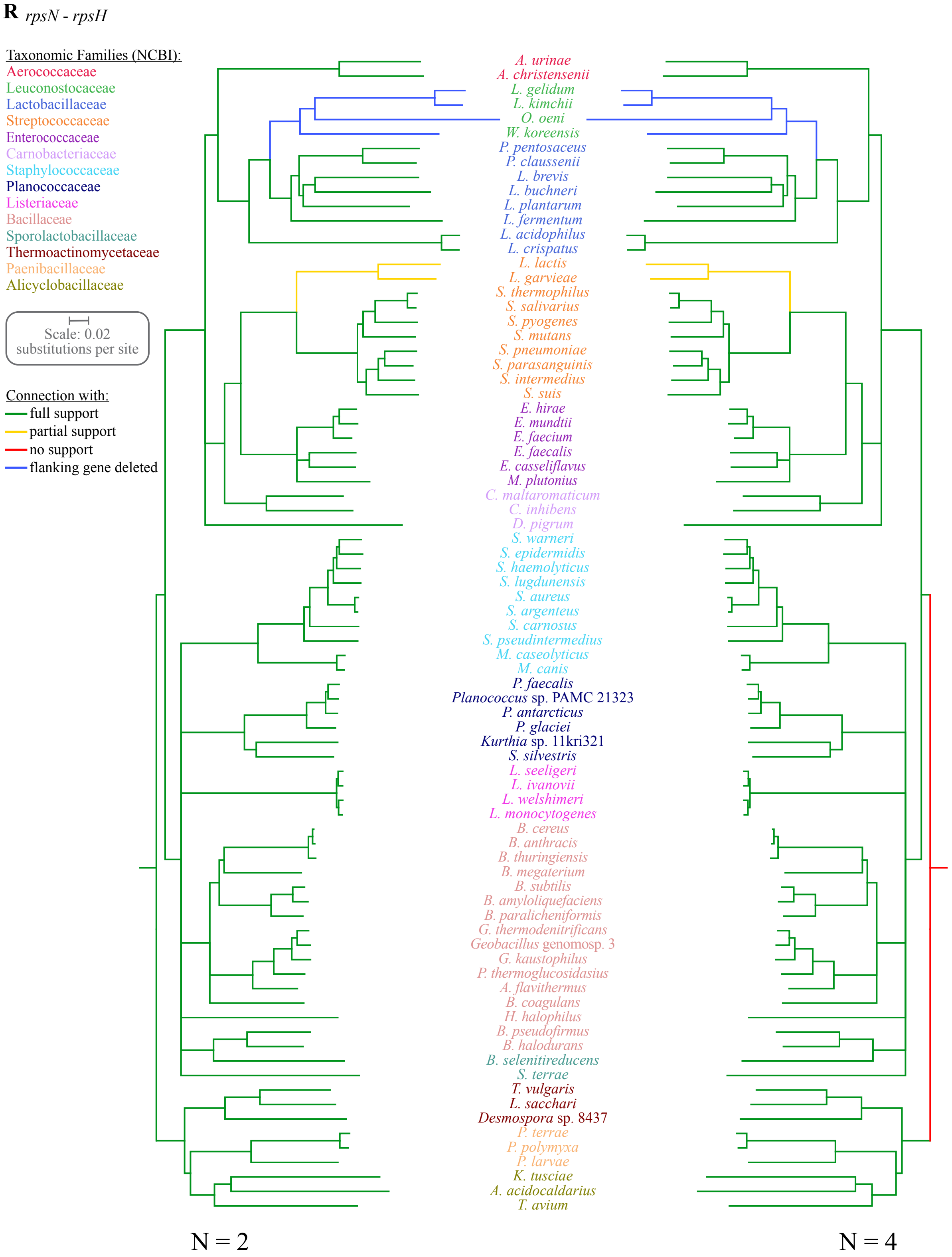
R rpsN - rpsH
Taxonomic Families (NCBI):
Aerococcaceae
Leuconostocaceae
Lactobacillaceae
Streptococcaceae
Enterococcaceae
Carnobacteriaceae
Staphylococcaceae
Planococcaceae
Listeriaceae
Bacillaceae
Sporolactobacillaceae
Thermoactinomycetaceae
Paenibacillaceae
Alicyclobacillaceae
Scale: 0.02 substitutions per site
Connection with:
full support
partial support
no support
flanking gene deleted
A. urinae
A. christensenii
L. gelidum
L. kimchii
O. oeni
W. koreensis
P. pentosaceus
P. claussenii
L. brevis
L. buchneri
L. plantarum
L. fermentum
L. acidophilus
L. crispatus
L. lactis
L. garvieae
S. thermophilus
S. salivarius
S. pyogenes
S. mutans
S. pneumoniae
S. parasanguinis
S. intermedius
S. suis
E. hirae
E. mundtii
E. faecium
E. faecalis
E. casseliflavus
M. plutonius
C. maltaromaticum
C. inhibens
D. pigrum
S. warneri
S. epidermidis
S. haemolyticus
S. lugdunensis
S. aureus
S. argenteus
S. carnosus
S. pseudintermedius
M. caseolyticus
M. canis
P. faecalis
Planococcus sp. PAMC 21323
P. antarcticus
P. glaciei
Kurthia sp. 11kri321
S. silvestris
L. seeligeri
L. ivanovii
L. welshimeri
L. monocytogenes
B. cereus
B. anthracis
B. thuringiensis
B. megaterium
B. subtilis
B. amyloliquefaciens
B. paralicheniformis
G. thermodenitrificans
Geobacillus genomosp. 3
G. kaustophilus
P. thermoglucosidasius
A. flavithermus
B. coagulans
H. halophilus
B. pseudofirmus
B. halodurans
B. selenitireducens
S. terrae
T. vulgaris
L. sacchari
Desmospora sp. 8437
P. terrae
P. polymyxa
P. larvae
K. tusciae
A. acidocaldarius
T. avium
N = 2
N = 4

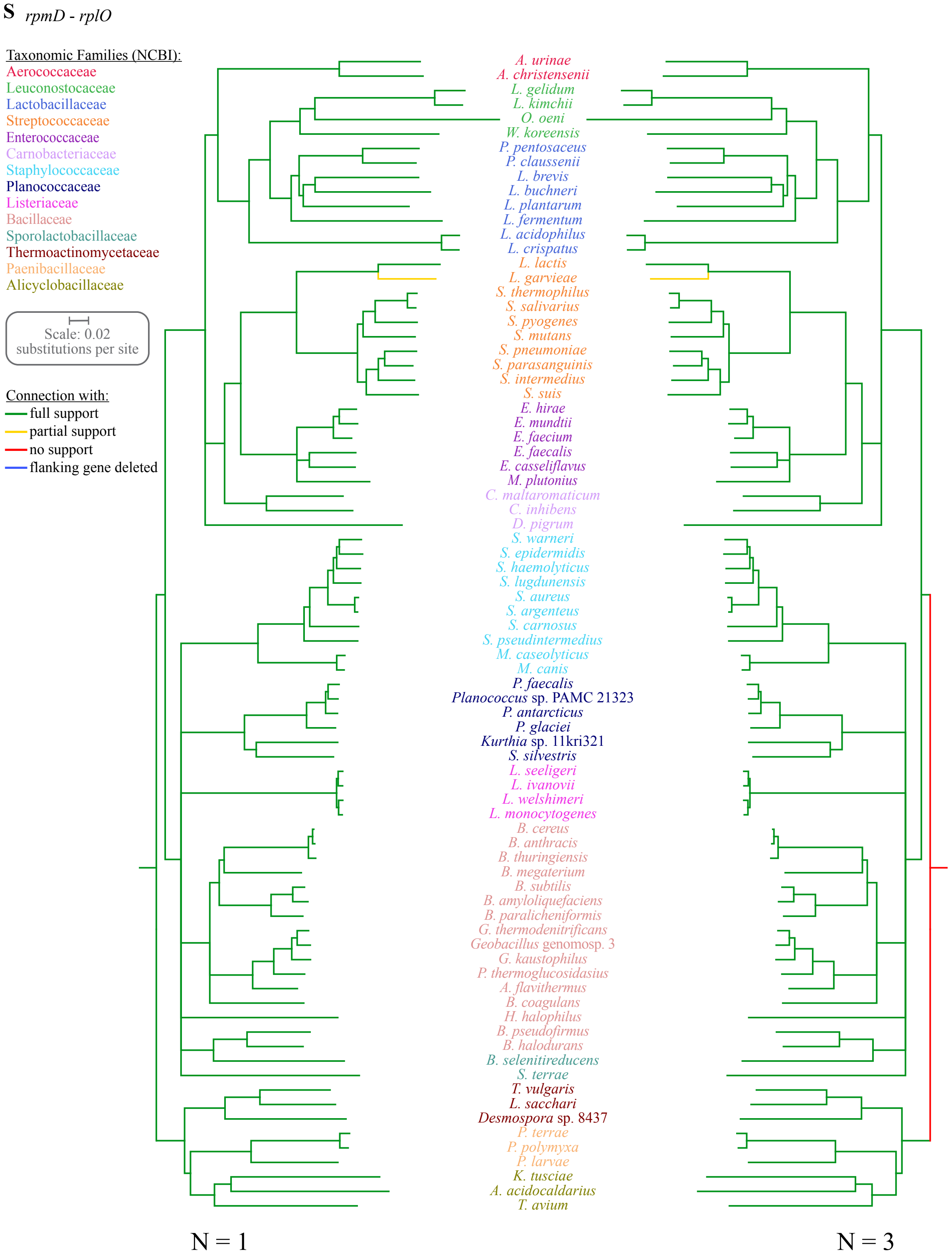

S rpmD - rplO
Taxonomic Families (NCBI):
Aerococcaceae
Leuconostocaceae
Lactobacillaceae
Streptococcaceae
Enterococcaceae
Carnobacteriaceae
Staphylococcaceae
Planococcaceae
Listeriaceae
Bacillaceae
Sporolactobacillaceae
Thermoactinomycetaceae
Paenibacillaceae
Alicyclobacillaceae
Scale: 0.02 substitutions per site
Connection with:
full support
partial support
no support
flanking gene deleted
A. urinae
A. christensenii
L. gelidum
L. kimchii
O. oeni
W. koreensis
P. pentosaceus
P. claussenii
L. brevis
L. buchneri
L. plantarum
L. fermentum
L. acidophilus
L. crispatus
L. lactis
L. garvieae
S. thermophilus
S. salivarius
S. pyogenes
S. mutans
S. pneumoniae
S. parasanguinis
S. intermedius
S. suis
E. hirae
E. mundtii
E. faecium
E. faecalis
E. casseliflavus
M. plutonius
C. maltaromaticum
C. inhibens
D. pigrum
S. warneri
S. epidermidis
S. haemolyticus
S. lugdunensis
S. aureus
S. argenteus
S. carnosus
S. pseudintermedius
M. caseolyticus
M. canis
P. faecalis
Planococcus sp. PAMC 21323
P. antarcticus
P. glaciei
Kurthia sp. 11kri321
S. silvestris
L. seeligeri
L. ivanovii
L. welshimeri
L. monocytogenes
B. cereus
B. anthracis
B. thuringiensis
B. megaterium
B. subtilis
B. amyloliquefaciens
B. paralicheniformis
G. thermodenitrificans
Geobacillus genomosp. 3
G. kaustophilus
P. thermoglucosidasius
A. flavithermus
B. coagulans
H. halophilus
B. pseudofirmus
B. halodurans
B. selenitireducens
S. terrae
T. vulgaris
L. sacchari
Desmospora sp. 8437
P. terrae
P. polymyxa
P. larvae
K. tusciae
A. acidocaldarius
T. avium
N = 1
N = 3

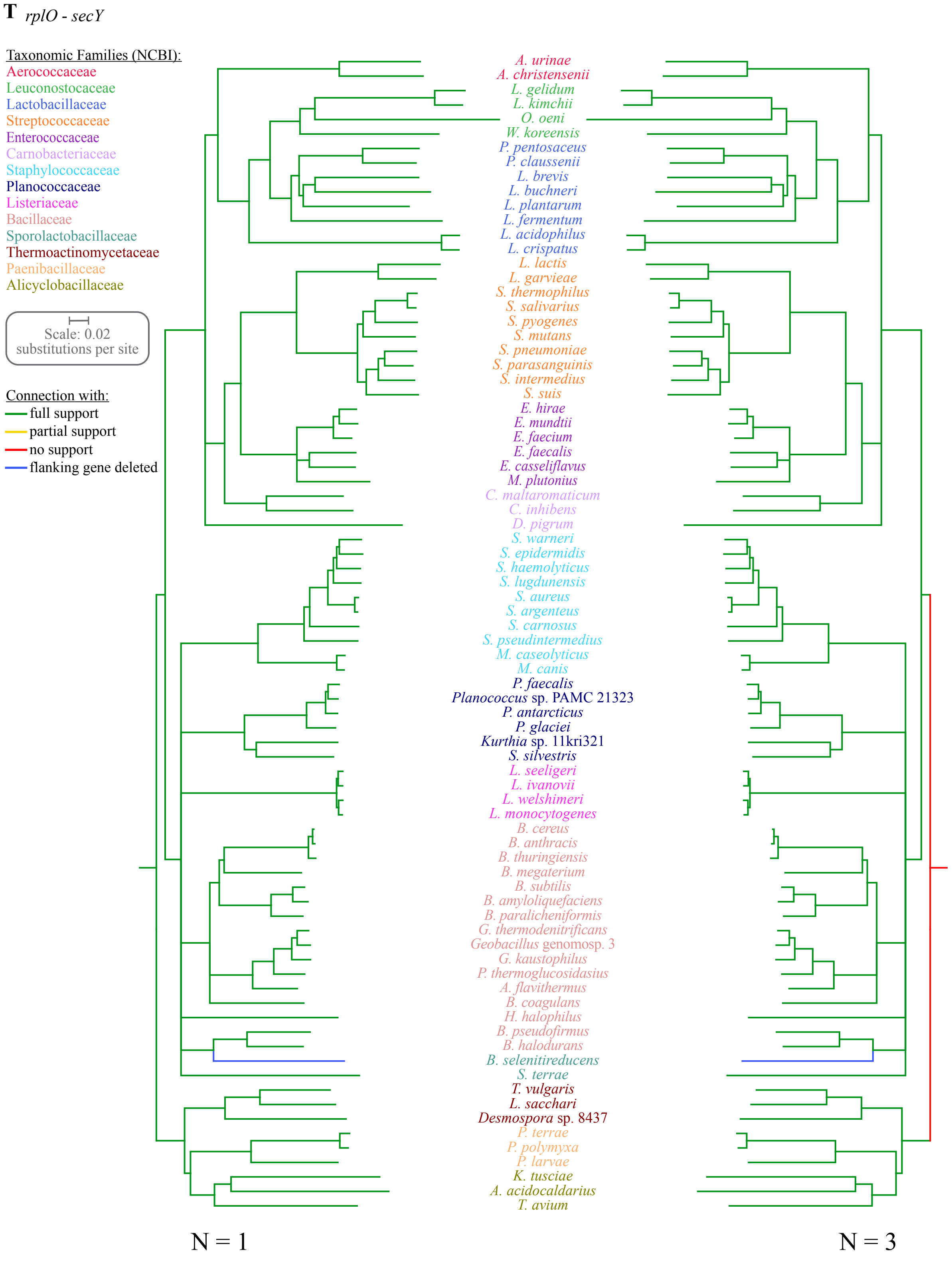
T rplO - secY
Taxonomic Families (NCBI):
Aerococcaceae
Leuconostocaceae
Lactobacillaceae
Streptococcaceae
Enterococcaceae
Carnobacteriaceae
Staphylococcaceae
Planococcaceae
Listeriaceae
Bacillaceae
Sporolactobacillaceae
Thermoactinomycetaceae
Paenibacillaceae
Alicyclobacillaceae
Scale: 0.02 substitutions per site
Connection with:
full support
partial support
no support
flanking gene deleted
A. urinae
A. christensenii
L. gelidum
L. kimchii
O. oeni
W. koreensis
P. pentosaceus
P. claussenii
L. brevis
L. buchneri
L. plantarum
L. fermentum
L. acidophilus
L. crispatus
L. lactis
L. garvieae
S. thermophilus
S. salivarius
S. pyogenes
S. mutans
S. pneumoniae
S. parasanguinis
S. intermedius
S. suis
E. hirae
E. mundtii
E. faecium
E. faecalis
E. casseliflavus
M. plutonius
C. maltaromaticum
C. inhibens
D. pigrum
S. warneri
S. epidermidis
S. haemolyticus
S. lugdunensis
S. aureus
S. argenteus
S. carnosus
S. pseudintermedius
M. caseolyticus
M. canis
P. faecalis
Planococcus sp. PAMC 21323
P. antarcticus
P. glaciei
Kurthia sp. 11kri321
S. silvestris
L. seeligeri
L. ivanovii
L. welshimeri
L. monocytogenes
B. cereus
B. anthracis
B. thuringiensis
B. megaterium
B. subtilis
B. amyloliquefaciens
B. paralicheniformis
G. thermodenitrificans
Geobacillus genomosp. 3
G. kaustophilus
P. thermoglucosidasius
A. flavithermus
B. coagulans
H. halophilus
B. pseudofirmus
B. halodurans
B. selenitireducens
S. terrae
T. vulgaris
L. sacchari
Desmospora sp. 8437
P. terrae
P. polymyxa
P. larvae
K. tusciae
A. acidocaldarius
T. avium
N = 1
N = 3

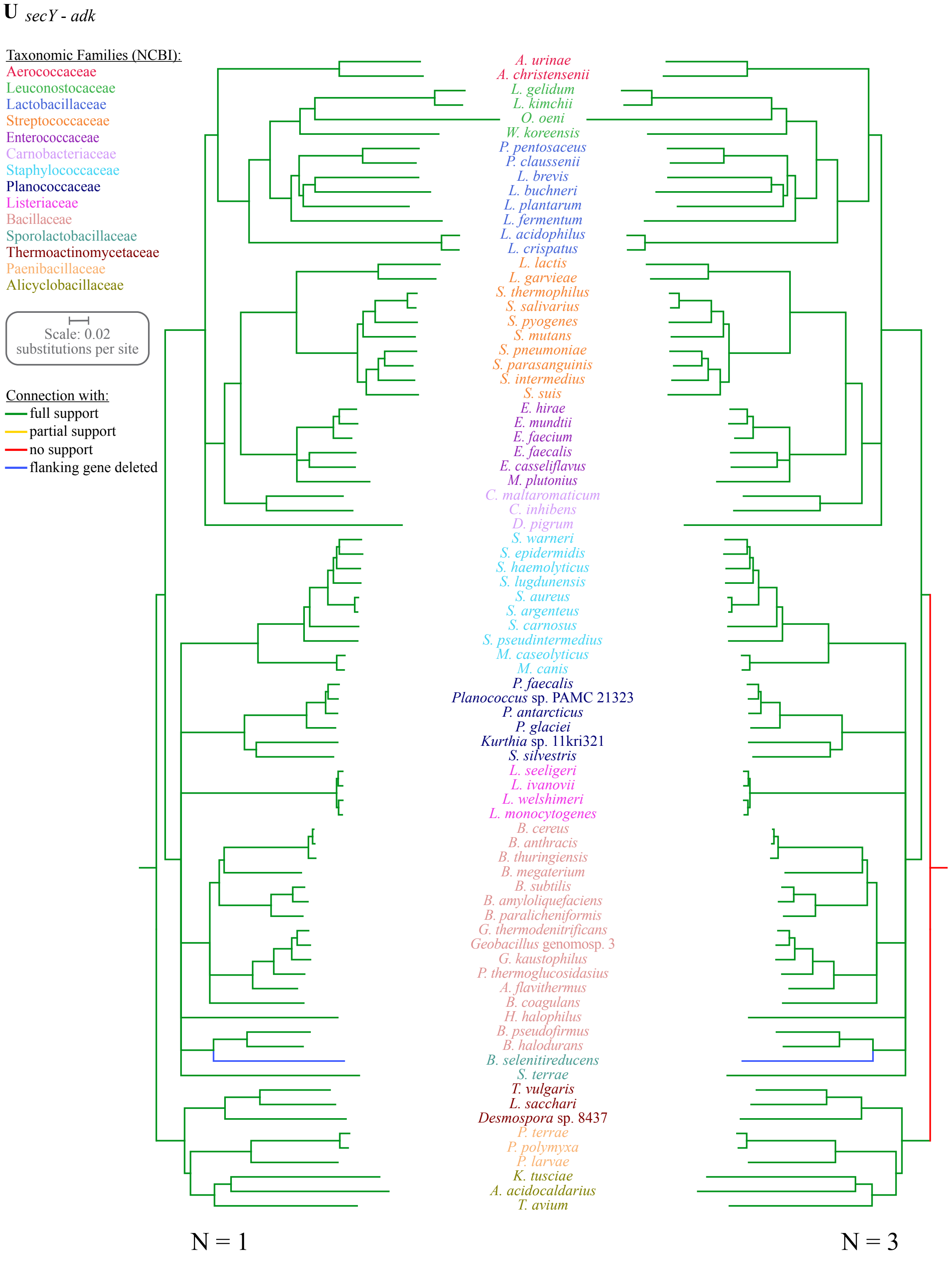
U secY - adk
Taxonomic Families (NCBI):
Aerococcaceae
Leuconostocaceae
Lactobacillaceae
Streptococcaceae
Enterococcaceae
Carnobacteriaceae
Staphylococcaceae
Planococcaceae
Listeriaceae
Bacillaceae
Sporolactobacillaceae
Thermoactinomycetaceae
Paenibacillaceae
Alicyclobacillaceae
Scale: 0.02 substitutions per site
Connection with:
full support
partial support
no support
flanking gene deleted
A. urinae
A. christensenii
L. gelidum
L. kimchii
O. oeni
W. koreensis
P. pentosaceus
P. claussenii
L. brevis
L. buchneri
L. plantarum
L. fermentum
L. acidophilus
L. crispatus
L. lactis
L. garvieae
S. thermophilus
S. salivarius
S. pyogenes
S. mutans
S. pneumoniae
S. parasanguinis
S. intermedius
S. suis
E. hirae
E. mundtii
E. faecium
E. faecalis
E. casseliflavus
M. plutonius
C. maltaromaticum
C. inhibens
D. pigrum
S. warneri
S. epidermidis
S. haemolyticus
S. lugdunensis
S. aureus
S. argenteus
S. carnosus
S. pseudintermedius
M. caseolyticus
M. canis
P. faecalis
Planococcus sp. PAMC 21323
P. antarcticus
P. glaciei
Kurthia sp. 11kri321
S. silvestris
L. seeligeri
L. ivanovii
L. welshimeri
L. monocytogenes
B. cereus
B. anthracis
B. thuringiensis
B. megaterium
B. subtilis
B. amyloliquefaciens
B. paralicheniformis
G. thermodenitrificans
Geobacillus genomosp. 3
G. kaustophilus
P. thermoglucosidasius
A. flavithermus
B. coagulans
H. halophilus
B. pseudofirmus
B. halodurans
B. selenitireducens
S. terrae
T. vulgaris
L. sacchari
Desmospora sp. 8437
P. terrae
P. polymyxa
P. larvae
K. tusciae
A. acidocaldarius
T. avium
N = 1
N = 3

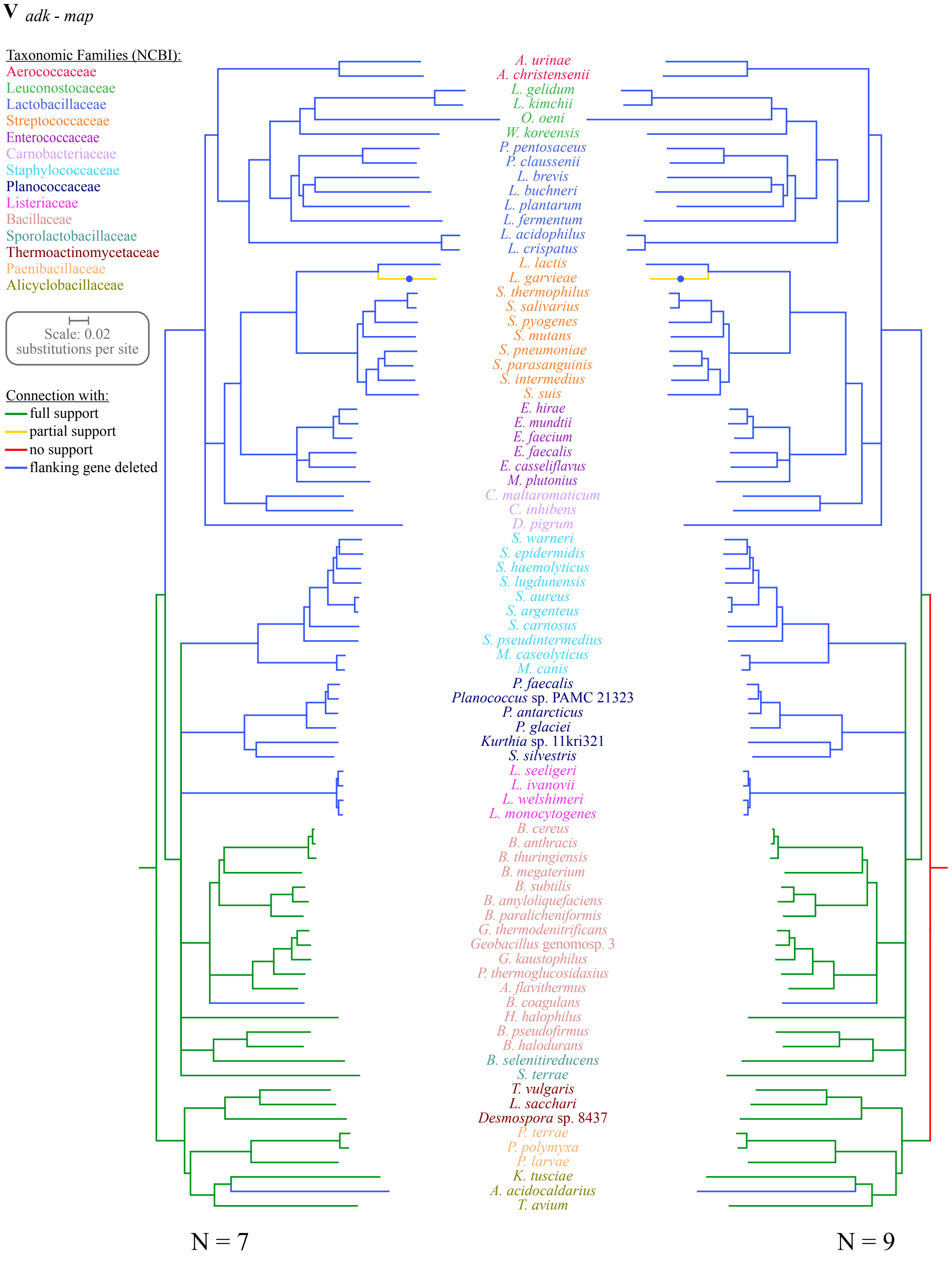
V adk - map
Taxonomic Families (NCBI):
Aerococcaceae
Leuconostocaceae
Lactobacillaceae
Streptococcaceae
Enterococcaceae
Carnobacteriaceae
Staphylococcaceae
Planococcaceae
Listeriaceae
Bacillaceae
Sporolactobacillaceae
Thermoactinomycetaceae
Paenibacillaceae
Alicyclobacillaceae
Scale: 0.02
substitutions per site
Connection with:
full support
partial support
no support
flanking gene deleted
A. urinae
A. christensenii
L. gelidum
L. kimchii
O. oeni
W. koreensis
P. pentosaceus
P. claussenii
L. brevis
L. buchneri
L. plantarum
L. fermentum
L. acidophilus
L. crispatus
L. lactis
L. garvieae
S. thermophilus
S. salivarius
S. pyogenes
S. mutans
S. pneumoniae
S. parasanguinis
S. intermedius
S. suis
E. hirae
E. mundtii
E. faecium
E. faecalis
E. casseliflavus
M. plutonius
C. maltaromaticum
C. inhibens
D. pigrum
S. warneri
S. epidermidis
S. haemolyticus
S. lugdunensis
S. aureus
S. argenteus
S. carnosus
S. pseudintermedius
M. caseolyticus
M. canis
P. faecalis
Planococcus sp. PAMC 21323
P. antarcticus
P. glaciei
Kurthia sp. 11kri321
S. silvestris
L. seeligeri
L. ivanovii
L. welshimeri
L. monocytogenes
B. cereus
B. anthracis
B. thuringiensis
B. megaterium
B. subtilis
B. amyloliquefaciens
B. paralicheniformis
G. thermodenitrificans
Geobacillus genomosp. 3
G. kaustophilus
P. thermoglucosidasius
A. flavithermus
B. coagulans
H. halophilus
B. pseudofirmus
B. halodurans
B. selenitireducens
S. terrae
T. vulgaris
L. sacchari
Desmospora sp. 8437
P. terrae
P. polymyxa
P. larvae
K. tusciae
A. acidocaldarius
T. avium
N = 7
N = 9

W *map - infA*

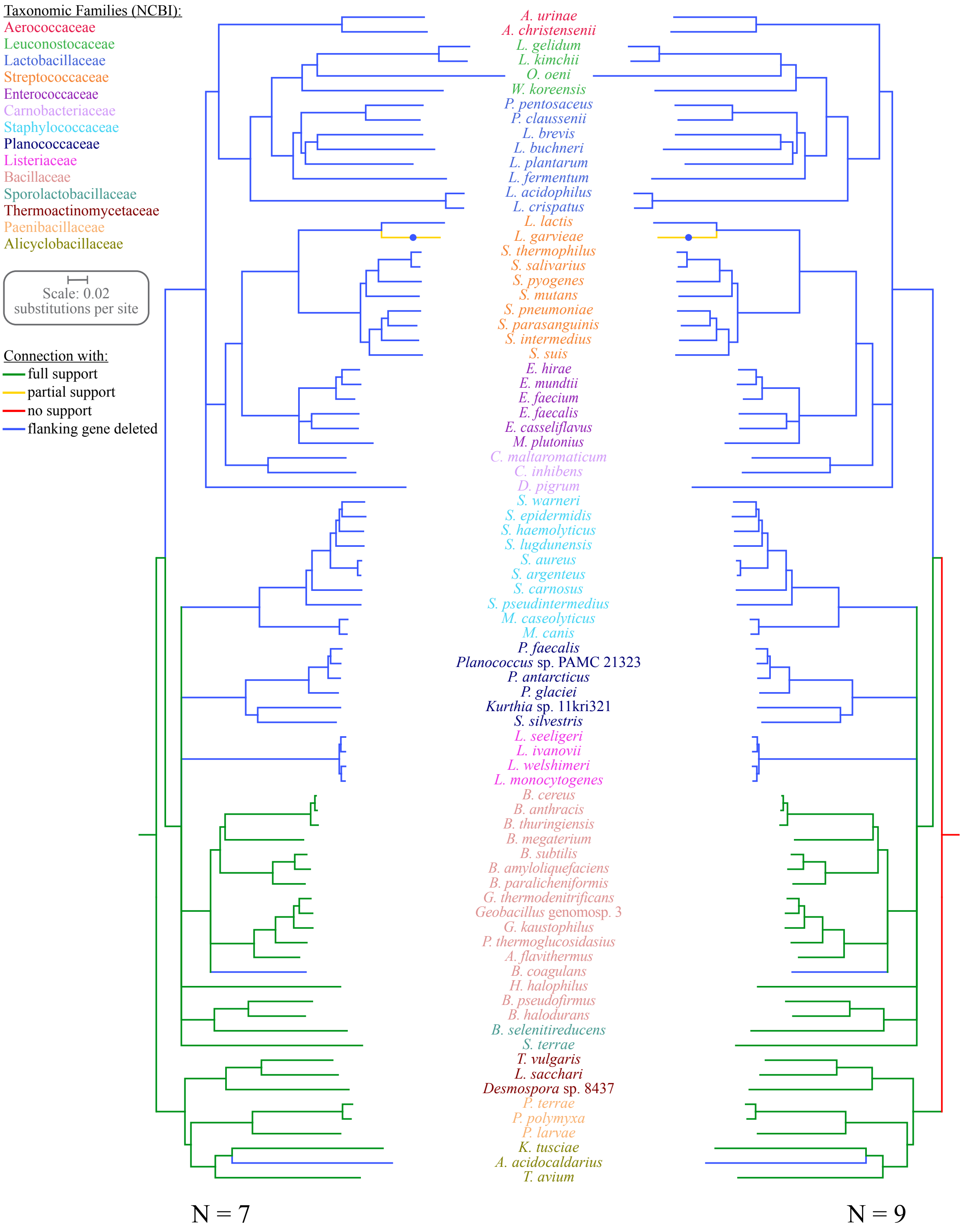
Taxonomic Families (NCBI):
Aerococcaceae
Leuconostocaceae
Lactobacillaceae
Streptococcaceae
Enterococcaceae
Carnobacteriaceae
Staphylococcaceae
Planococcaceae
Listeriaceae
Bacillaceae
Sporolactobacillaceae
Thermoactinomycetaceae
Paenibacillaceae
Alicyclobacillaceae
Scale: 0.02 substitutions per site
Connection with:
full support
partial support
no support
flanking gene deleted
A. urinae
A. christensenii
L. gelidum
L. kimchii
O. oeni
W. koreensis
P. pentosaceus
P. claussenii
L. brevis
L. buchneri
L. plantarum
L. fermentum
L. acidophilus
L. crispatus
L. lactis
L. garvieae
S. thermophilus
S. salivarius
S. pyogenes
S. mutans
S. pneumoniae
S. parasanguinis
S. intermedius
S. suis
E. hirae
E. mundtii
E. faecium
E. faecalis
E. casseliflavus
M. plutonius
C. maltaromaticum
C. inhibens
D. pigrum
S. warneri
S. epidermidis
S. haemolyticus
S. lugdunensis
S. aureus
S. argenteus
S. carnosus
S. pseudintermedius
M. caseolyticus
M. canis
P. faecalis
Planococcus sp. PAMC 21323
P. antarcticus
P. glaciei
Kurthia sp. 11kri321
S. silvestris
L. seeligeri
L. ivanovii
L. welshimeri
L. monocytogenes
B. cereus
B. anthracis
B. thuringiensis
B. megaterium
B. subtilis
B. amyloliquefaciens
B. paralicheniformis
G. thermodenitrificans
Geobacillus genomosp. 3
G. kaustophilus
P. thermoglucosidasius
A. flavithermus
B. coagulans
H. halophilus
B. pseudofirmus
B. halodurans
B. selenitireducens
S. terrae
T. vulgaris
L. sacchari
Desmospora sp. 8437
P. terrae
P. polymyxa
P. larvae
K. tusciae
A. acidocaldarius
T. avium
N = 7
N = 9

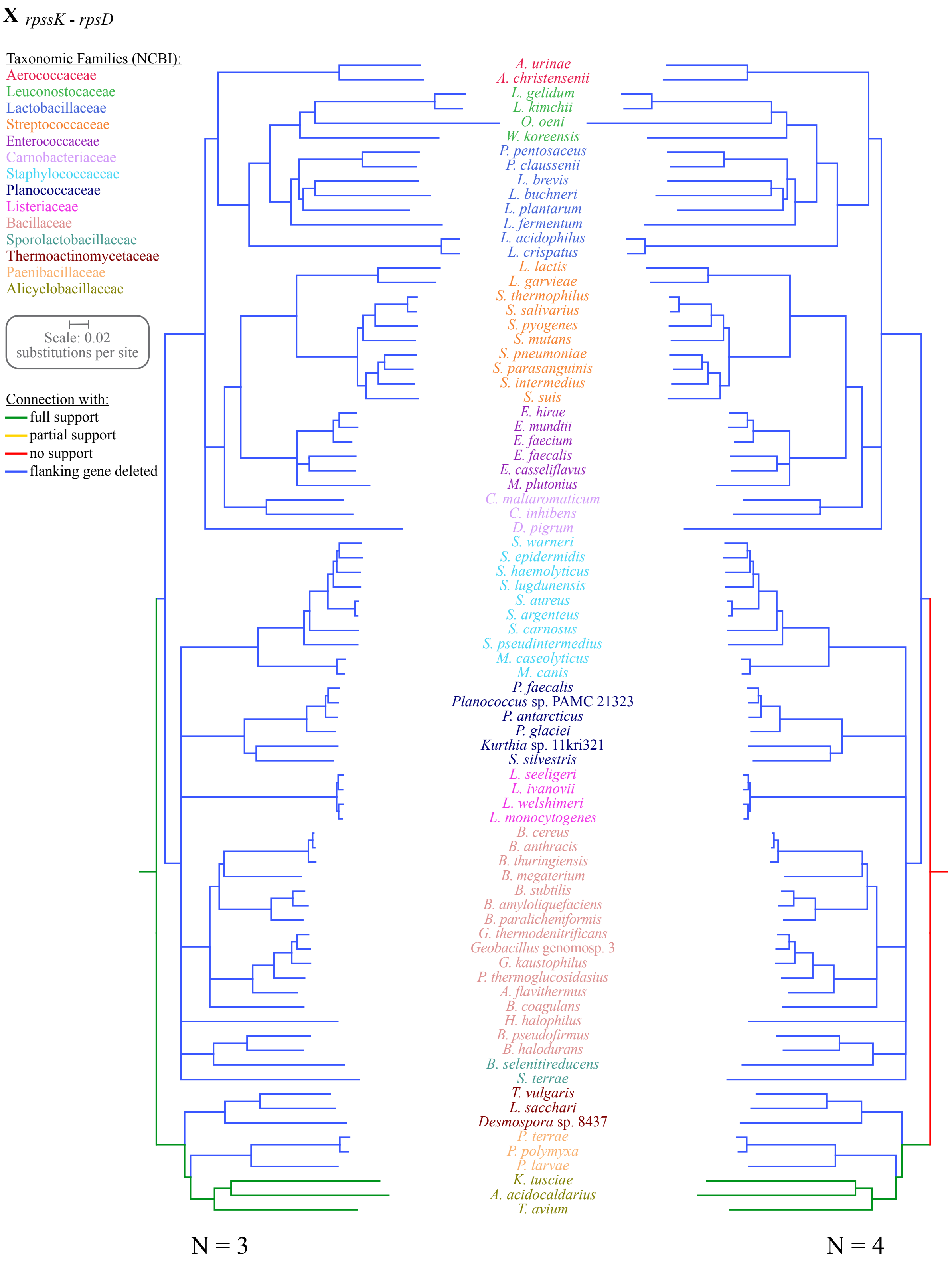
X rpssK - rpsD
Taxonomic Families (NCBI):
Aerococcaceae
Leuconostocaceae
Lactobacillaceae
Streptococcaceae
Enterococcaceae
Carnobacteriaceae
Staphylococcaceae
Planococcaceae
Listeriaceae
Bacillaceae
Sporolactobacillaceae
Thermoactinomycetaceae
Paenibacillaceae
Alicyclobacillaceae
Scale: 0.02 substitutions per site
Connection with:
full support
partial support
no support
flanking gene deleted
A. urinae
A. christensenii
L. gelidum
L. kimchii
O. oeni
W. koreensis
P. pentosaceus
P. claussenii
L. brevis
L. buchneri
L. plantarum
L. fermentum
L. acidophilus
L. crispatus
L. lactis
L. garvieae
S. thermophilus
S. salivarius
S. pyogenes
S. mutans
S. pneumoniae
S. parasanguinis
S. intermedius
S. suis
E. hirae
E. mundtii
E. faecium
E. faecalis
E. casseliflavus
M. plutonius
C. maltaromaticum
C. inhibens
D. pigrum
S. warneri
S. epidermidis
S. haemolyticus
S. lugdunensis
S. aureus
S. argenteus
S. carnosus
S. pseudintermedius
M. caseolyticus
M. canis
P. faecalis
Planococcus sp. PAMC 21323
P. antarcticus
P. glaciei
Kurthia sp. 11kri321
S. silvestris
L. seeligeri
L. ivanovii
L. welshimeri
L. monocytogenes
B. cereus
B. anthracis
B. thuringiensis
B. megaterium
B. subtilis
B. amyloliquefaciens
B. paralicheniformis
G. thermodenitrificans
Geobacillus genomosp. 3
G. kaustophilus
P. thermoglucosidasius
A. flavithermus
B. coagulans
H. halophilus
B. pseudofirmus
B. halodurans
B. selenitireducens
S. terrae
T. vulgaris
L. sacchari
Desmospora sp. 8437
P. terrae
P. polymyxa
P. larvae
K. tusciae
A. acidocaldarius
T. avium
N = 3
N = 4

Y *rpsD - rpoA*

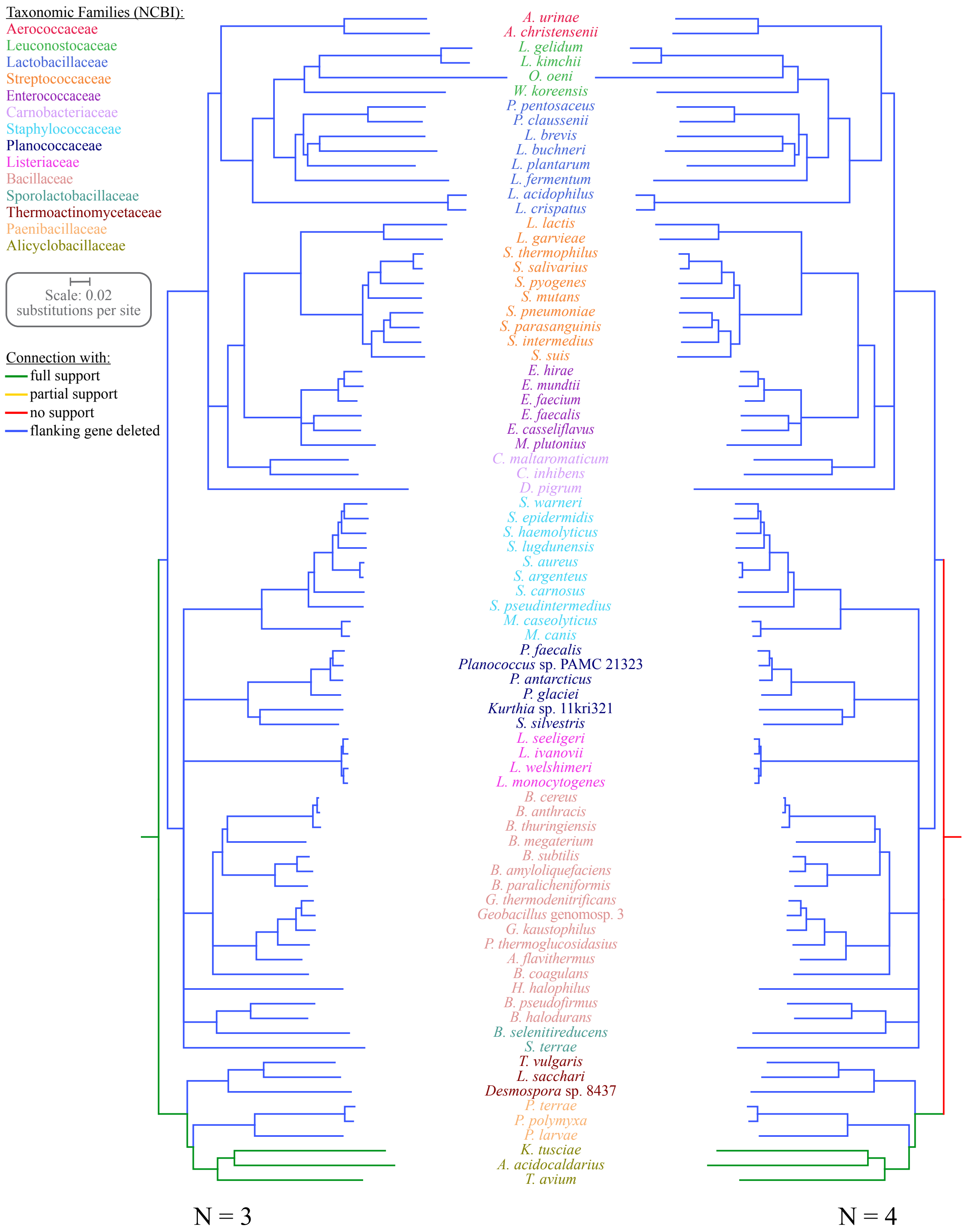

Taxonomic Families (NCBI):
Aerococcaceae
Leuconostocaceae
Lactobacillaceae
Streptococcaceae
Enterococcaceae
Carnobacteriaceae
Staphylococcaceae
Planococcaceae
Listeriaceae
Bacillaceae
Sporolactobacillaceae
Thermoactinomycetaceae
Paenibacillaceae
Alicyclobacillaceae
Scale: 0.02 substitutions per site
Connection with:
full support
partial support
no support
flanking gene deleted
A. urinae
A. christensenii
L. gelidum
L. kimchii
O. oeni
W. koreensis
P. pentosaceus
P. claussenii
L. brevis
L. buchneri
L. plantarum
L. fermentum
L. acidophilus
L. crispatus
L. lactis
L. garvieae
S. thermophilus
S. salivarius
S. pyogenes
S. mutans
S. pneumoniae
S. parasanguinis
S. intermedius
S. suis
E. hirae
E. mundtii
E. faecium
E. faecalis
E. casseliflavus
M. plutonius
C. maltaromaticum
C. inhibens
D. pigrum
S. warneri
S. epidermidis
S. haemolyticus
S. lugdunensis
S. aureus
S. argenteus
S. carnosus
S. pseudintermedius
M. caseolyticus
M. canis
P. faecalis
Planococcus sp. PAMC 21323
P. antarcticus
P. glaciei
Kurthia sp. 11kri321
S. silvestris
L. seeligeri
L. ivanovii
L. welshimeri
L. monocytogenes
B. cereus
B. anthracis
B. thuringiensis
B. megaterium
B. subtilis
B. amyloliquefaciens
B. paralicheniformis
G. thermodenitrificans
Geobacillus genomosp. 3
G. kaustophilus
P. thermoglucosidasius
A. flavithermus
B. coagulans
H. halophilus
B. pseudofirmus
B. halodurans
B. selenitireducens
S. terrae
T. vulgaris
L. sacchari
Desmospora sp. 8437
P. terrae
P. polymyxa
P. larvae
K. tusciae
A. acidocaldarius
T. avium
N = 3
N = 4

Z *rplQ - ecfA1*

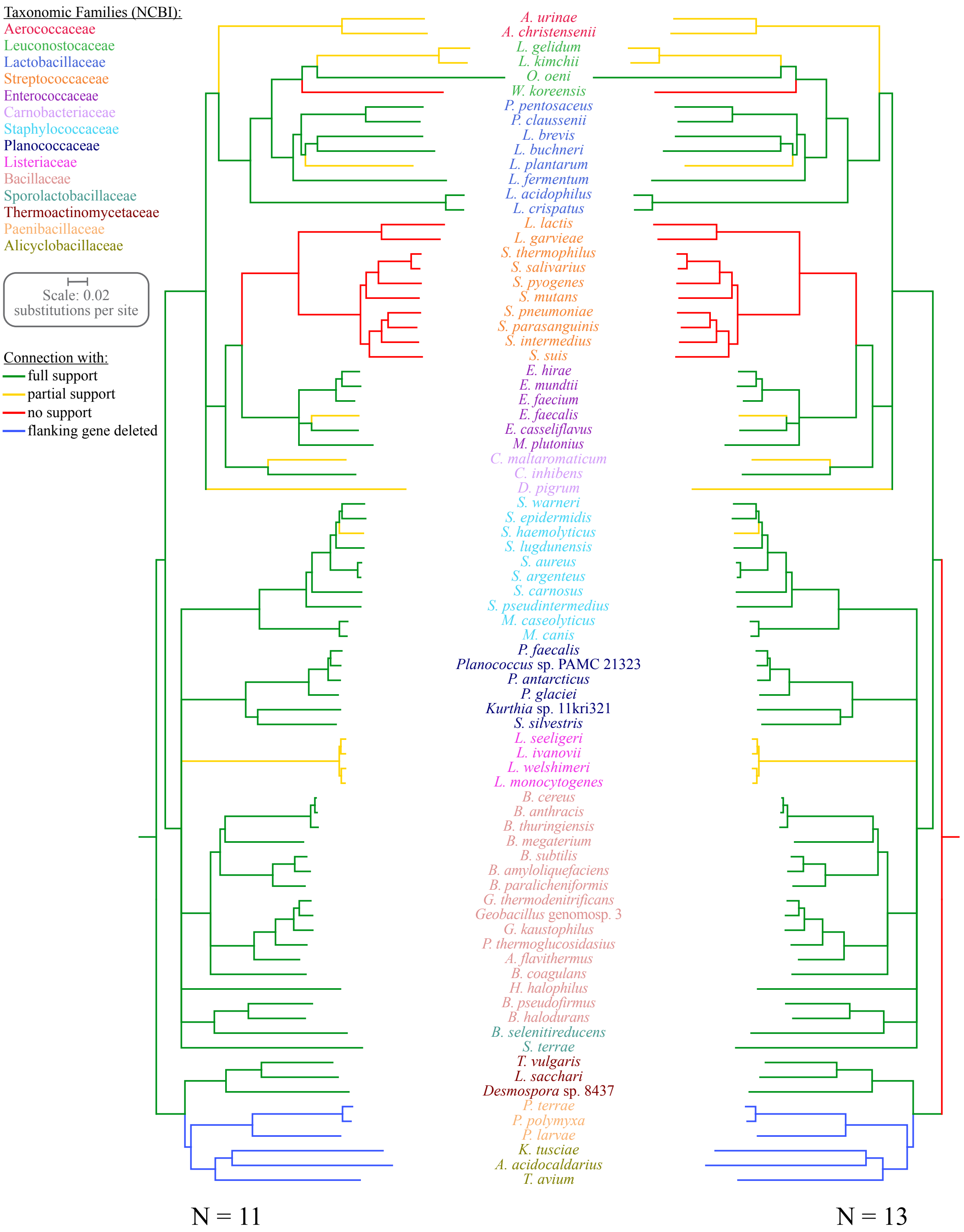
Taxonomic Families (NCBI):
Aerococcaceae
Leuconostocaceae
Lactobacillaceae
Streptococcaceae
Enterococcaceae
Carnobacteriaceae
Staphylococcaceae
Planococcaceae
Listeriaceae
Bacillaceae
Sporolactobacillaceae
Thermoactinomycetaceae
Paenibacillaceae
Alicyclobacillaceae
Scale: 0.02 substitutions per site
Connection with:
full support
partial support
no support
flanking gene deleted
A. urinae
A. christensenii
L. gelidum
L. kimchii
O. oeni
W. koreensis
P. pentosaceus
P. claussenii
L. brevis
L. buchneri
L. plantarum
L. fermentum
L. acidophilus
L. crispatus
L. lactis
L. garvieae
S. thermophilus
S. salivarius
S. pyogenes
S. mutans
S. pneumoniae
S. parasanguinis
S. intermedius
S. suis
E. hirae
E. mundtii
E. faecium
E. faecalis
E. casseliflavus
M. plutonius
C. maltaromaticum
C. inhibens
D. pigrum
S. warneri
S. epidermidis
S. haemolyticus
S. lugdunensis
S. aureus
S. argenteus
S. carnosus
S. pseudintermedius
M. caseolyticus
M. canis
P. faecalis
Planococcus sp. PAMC 21323
P. antarcticus
P. glaciei
Kurthia sp. 11kri321
S. silvestris
L. seeligeri
L. ivanovii
L. welshimeri
L. monocytogenes
B. cereus
B. anthracis
B. thuringiensis
B. megaterium
B. subtilis
B. amyloliquefaciens
B. paralicheniformis
G. thermodenitrificans
Geobacillus genomosp. 3
G. kaustophilus
P. thermoglucosidasius
A. flavithermus
B. coagulans
H. halophilus
B. pseudofirmus
B. halodurans
B. selenitireducens
S. terrae
T. vulgaris
L. sacchari
Desmospora sp. 8437
P. terrae
P. polymyxa
P. larvae
K. tusciae
A. acidocaldarius
T. avium
N = 11
N = 13

AA *ecfT* - *truA*

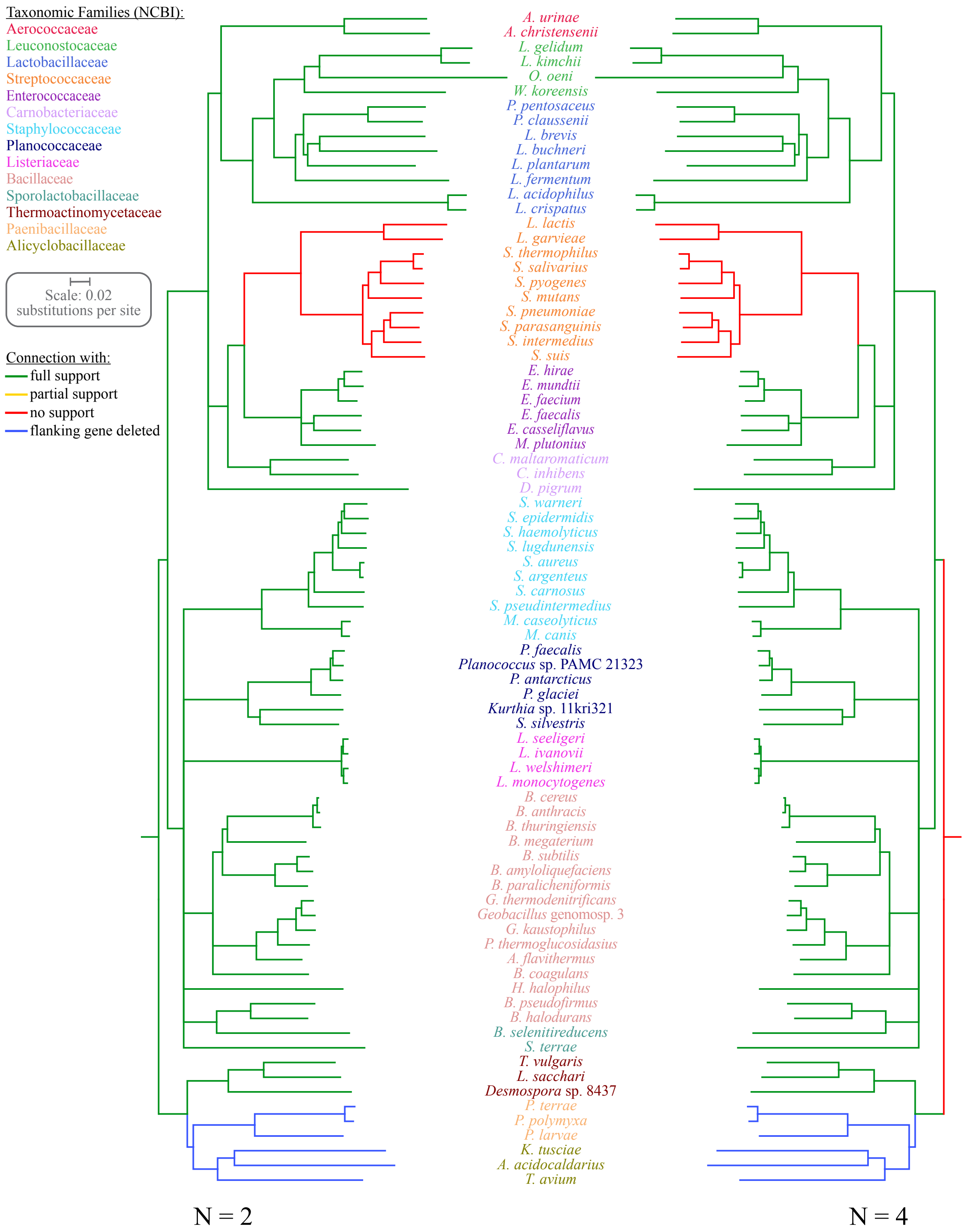
Taxonomic Families (NCBI):
Aerococcaceae
Leuconostocaceae
Lactobacillaceae
Streptococcaceae
Enterococcaceae
Carnobacteriaceae
Staphylococcaceae
Planococcaceae
Listeriaceae
Bacillaceae
Sporolactobacillaceae
Thermoactinomycetaceae
Paenibacillaceae
Alicyclobacillaceae
Scale: 0.02 substitutions per site
Connection with:
full support
partial support
no support
flanking gene deleted
A. urinae
A. christensenii
L. gelidum
L. kimchii
O. oeni
W. koreensis
P. pentosaceus
P. claussenii
L. brevis
L. buchneri
L. plantarum
L. fermentum
L. acidophilus
L. crispatus
L. lactis
L. garvieae
S. thermophilus
S. salivarius
S. pyogenes
S. mutans
S. pneumoniae
S. parasanguinis
S. intermedius
S. suis
E. hirae
E. mundtii
E. faecium
E. faecalis
E. casseliflavus
M. plutonius
C. maltaromaticum
C. inhibens
D. pigrum
S. warneri
S. epidermidis
S. haemolyticus
S. lugdunensis
S. aureus
S. argenteus
S. carnosus
S. pseudintermedius
M. caseolyticus
M. canis
P. faecalis
Planococcus sp. PAMC 21323
P. antarcticus
P. glaciei
Kurthia sp. 11kri321
S. silvestris
L. seeligeri
L. ivanovii
L. welshimeri
L. monocytogenes
B. cereus
B. anthracis
B. thuringiensis
B. megaterium
B. subtilis
B. amyloliquefaciens
B. paralicheniformis
G. thermodenitrificans
Geobacillus genomosp. 3
G. kaustophilus
P. thermoglucosidasius
A. flavithermus
B. coagulans
H. halophilus
B. pseudofirmus
B. halodurans
B. selenitireducens
S. terrae
T. vulgaris
L. sacchari
Desmospora sp. 8437
P. terrae
P. polymyxa
P. larvae
K. tusciae
A. acidocaldarius
T. avium
N = 2
N = 4

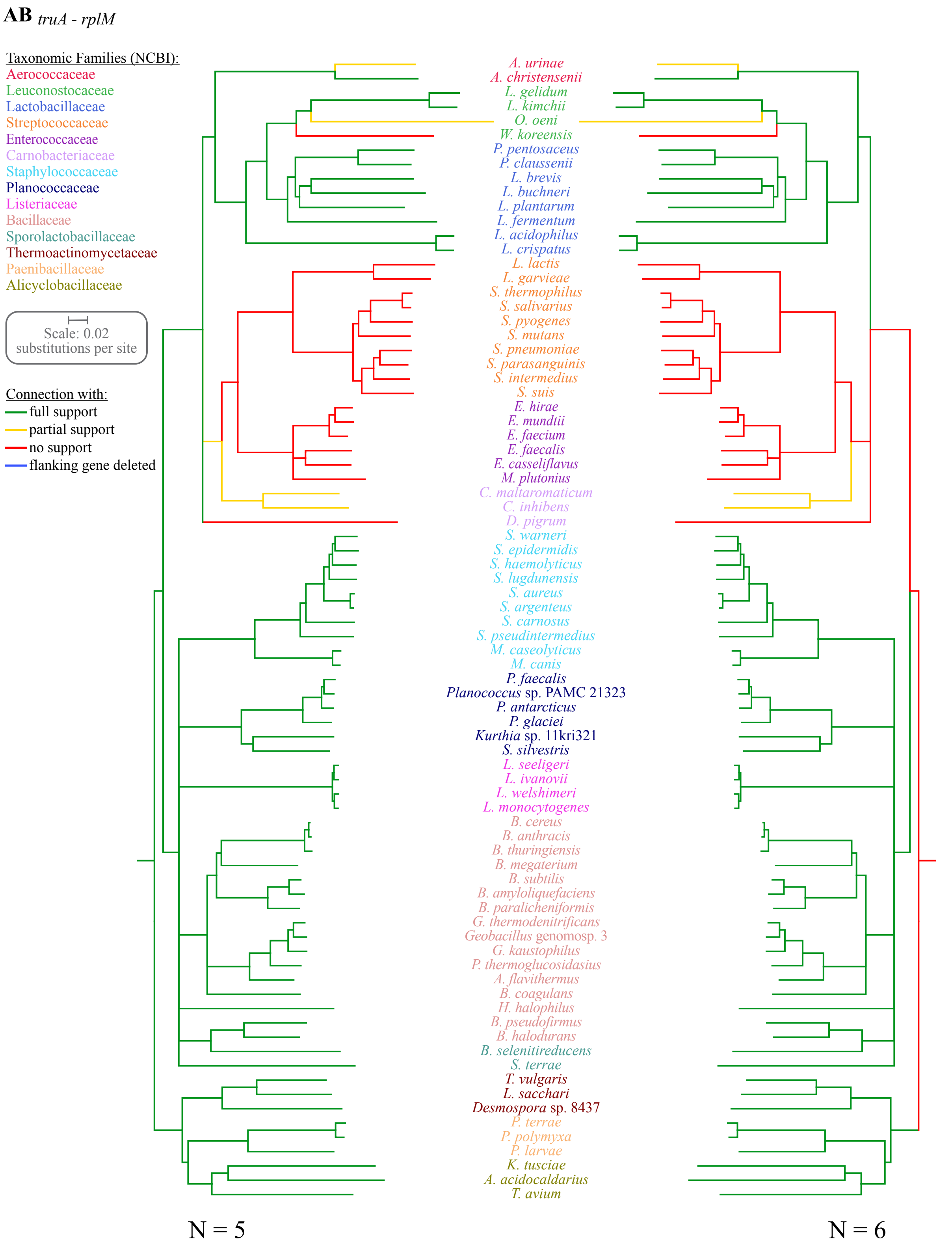
AB truA - rplM
Taxonomic Families (NCBI):
Aerococcaceae
Leuconostocaceae
Lactobacillaceae
Streptococcaceae
Enterococcaceae
Carnobacteriaceae
Staphylococcaceae
Planococcaceae
Listeriaceae
Bacillaceae
Sporolactobacillaceae
Thermoactinomycetaceae
Paenibacillaceae
Alicyclobacillaceae
Scale: 0.02
substitutions per site
Connection with:
full support
partial support
no support
flanking gene deleted
A. urinae
A. christensenii
L. gelidum
L. kimchii
O. oeni
W. koreensis
P. pentosaceus
P. claussenii
L. brevis
L. buchneri
L. plantarum
L. fermentum
L. acidophilus
L. crispatus
L. lactis
L. garvieae
S. thermophilus
S. salivarius
S. pyogenes
S. mutans
S. pneumoniae
S. parasanguinis
S. intermedius
S. suis
E. hirae
E. mundtii
E. faecium
E. faecalis
E. casseliflavus
M. plutonius
C. maltaromaticum
C. inhibens
D. pigrum
S. warneri
S. epidermidis
S. haemolyticus
S. lugdunensis
S. aureus
S. argenteus
S. carnosus
S. pseudintermedius
M. caseolyticus
M. canis
P. faecalis
Planococcus sp. PAMC 21323
P. antarcticus
P. glaciei
Kurthia sp. 11kri321
S. silvestris
L. seeligeri
L. ivanovii
L. welshimeri
L. monocytogenes
B. cereus
B. anthracis
B. thuringiensis
B. megaterium
B. subtilis
B. amyloliquefaciens
B. paralicheniformis
G. thermodenitrificans
Geobacillus genomosp. 3
G. kaustophilus
P. thermoglucosidasius
A. flavithermus
B. coagulans
H. halophilus
B. pseudofirmus
B. halodurans
B. selenitireducens
S. terrae
T. vulgaris
L. sacchari
Desmospora sp. 8437
P. terrae
P. polymyxa
P. larvae
K. tusciae
A. acidocaldarius
T. avium
N = 5
N = 6

FIG. S15. Decision trees used in the reconstruction of the ancestral operon cluster for all gene pairs that are not fully conserved throughout the *Bacilli* used in this study. The number of minimal state changes (N) of each gene pair was determined for the case that the gene pair was connected (left tree) or disconnected (right tree) within the last common ancestor of the displayed species. The definition of potential state changes is shown in supplementary fig. S13.

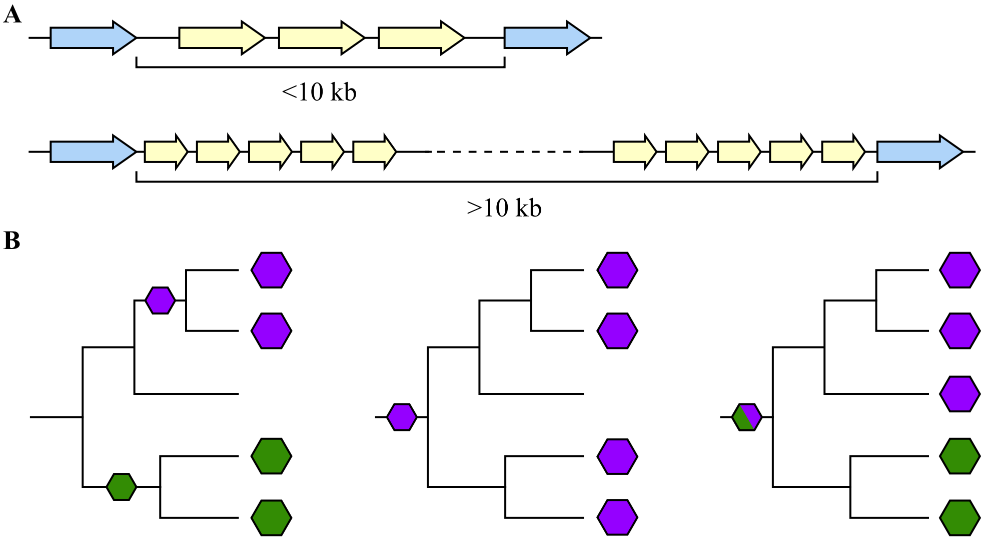

FIG. S16. Gene synteny analysis and identification of evolutionary events. (**A**) Layout for the genes included in the gene synteny analysis (yellow arrows) for interrupted gene pairs of the operon cluster (blue arrows). (**B**) Layout for identification process of evolutionary events. Novel gene neighbourhoods in modern species are depicted by purple and green hexagons. Evolutionary events that were identified to result in the specified outcome are depicted within the tree.

Table S1. Overview over genomes included in the analysis.

Species	Accession number
[Bacillus] selenitireducens MLS10	NC_014219
Acholeplasma laidlawii PG-8A	NC_010163
Acidaminococcus fermentans DSM 20731	NC_013740
Acidithiobacillus caldus SM-1	NC_015850
Acidithiobacillus ferrooxidans ATCC 23270	NC_011761
Acidobacterium capsulatum ATCC 51196	NC_012483
Acinetobacter baumannii ATCC 17978	CP000521
Acinetobacter sp. ADP1	NC_005966
Actinobacillus equuli str. 19392	CP007715
Actinobacillus indolicus str. AIFJ1607	CP038145
Actinobacillus pleuropneumoniae L20	NC_009053
Actinobacillus porcitonsillarum str. 9953L55	CP029206
Actinobacillus succinogenes 130Z	NC_009655
Actinobacillus suis H91-0380	NC_018690
Aerococcus christensenii str. CCUG28831	CP014159
Aerococcus urinae ACS-120-V-Col10a	NC_015278
Aeromonas hydrophila ATCC 7966	NC_008570
Aeromonas veronii B565	NC_015424
Alcanivorax borkumensis SK2	NC_008260
Alicyclobacillus acidocaldarius DSM 446	NC_013205
Alkalilimnicola ehrlichii MLHE-1	NC_008340
Alteromonas macleodii ATCC 27126	NC_018632
Anaerococcus prevotii DSM 20548	NC_013171
Anoxybacillus flavithermus WK1	NC_011567
Bacillus amyloliquefaciens CC178	NC_022653
Bacillus anthracis str. H9401	NC_017729
Bacillus cereus NC7401	NC_016771
Bacillus coagulans 36D1	NC_016023
Bacillus halodurans C-125	NC_002570
Bacillus megaterium WSH-002	NC_017138
Bacillus paralicheniformis ATCC 9945a	NC_021362
Bacillus pseudofirmus OF4	NC_013791
Bacillus subtilis PY79	NC_022898
Bacillus thuringiensis str. IS5056	NC_020376
Beggiatoa leptomitoformis str. D-401	CP018889
Budvicia aquatica str. FDAARGOS_387	NZ_PDDX01000001
Campylobacter jejuni 269.97	NC_009707
Candidatus Koribacter versatilis Ellin345	NC_008009
Cardiobacterium hominis str. NCTC10426	LR134365
Carnobacterium inhibens subsp. *gilichinskyi* str. WN1359	NC_022606
Carnobacterium maltaromaticum LMA28	NC_019425
Chromobacterium violaceum ATCC 12472	NC_005085
Chromohalobacter salexigens DSM 3043	NC_007963
Citrobacter koseri ATCC BAA-89	NC_009792
Clostridium perfringens ATCC 13124	NC_008261
Colwellia psychrerythraea 34H	NC_003910
Congregibacter litoralis KT71	NZ_CM002299

Coxiella burnetii str. RSA439	NZ_CP040059
Desmospora sp. 8437	GL892032
Dichelobacter nodosus VCS1703A	NC_009446
Dickeya zeae Ech586	NC_013592
Dolosigranulum pigrum str. 83VPs-KB5	CP041626
Edwardsiella tarda EIB202	NC_013508
Enterobacter sp. 638	NC_009436
Enterococcus casseliflavus EC20	NC_020995
Enterococcus faecalis D32	NC_018221
Enterococcus faecium Aus0085	NC_021994
Enterococcus hirae ATCC 9790	NC_018081
Enterococcus mundtii QU 25	NC_022878
Erwinia pyrifoliae DSM 12163	NC_017390
Erysipelothrix rhusiopathiae SY1027	NC_021354
Escherichia coli K12	NC_000913
Finegoldia magna ATCC 29328	NC_010376
Francisella tularensis SCHU S4	NC_006570
Frischella perrara str. PEB0191	CP009056
Geobacillus genomosp. 3 str. JF8	NC_022080
Geobacillus kaustophilus HTA426	NC_006510
Geobacillus thermodenitrificans NG80-2	NC_009328
Geobacter sulfurreducens KN400	NC_017454
Gilliamella apicola str. wkB1	CP007445
Haemophilus ducreyi 35000HP	NC_002940
Haemophilus influenzae Rd KW20	NC_000907
Haemophilus parainfluenzae T3T1	NC_015964
Haemophilus somnus 129PT	NC_008309
Hafnia paralvei str. FDAARGOS_158	CP014031
Hahella chejuensis KCTC 2396	NC_007645
Halobacillus halophilus DSM 2266	NC_017668
Halorhodospira halophila SL1	NC_008789
Helicobacter pylori J99	NC_000921
Idiomarina loihiensis L2TR	NC_006512
Klebsiella pneumoniae str. NCTC 418	CP028915
Kurthia sp. 11kri321	CP013217
Kyrpidia tusciae DSM 2912	NC_014098
Laceyella sacchari str. FBKL4.010	CP025943
Lactobacillus acidophilus La-14	NC_021181
Lactobacillus brevis ATCC 367	NC_008497
Lactobacillus buchneri NRRL B-30929	NC_015428
Lactobacillus crispatus ST1	NC_014106
Lactobacillus fermentum CECT 5716	NC_017465
Lactobacillus plantarum JDM1	NC_012984
Lactococcus garvieae ATCC 49156	NC_015930
Lactococcus lactis subsp. *cremoris* NZ9000	NC_017949
Leclercia sp. LSNIH3	CP026387
Legionella pneumophila str. Philadelphia 1	NC_002942
Leuconostoc gelidum JB7	NC_018631
Leuconostoc kimchii IMSNU 11154	NC_014136
Listeria ivanovii PAM 55	NC_016011

Listeria monocytogenes M7	NC_017537
Listeria seeligeri serovar 1/2b str. SLCC3954	NC_013891
Listeria welshimeri serovar 6b str. SLCC5334	NC_008555
Macrococcus canis str. KM0218	CP035309
Macrococcus caseolyticus JCSC5402	NC_011999
Mahella australiensis 50-1 BON	NC_015520
Mannheimia succiniciproducens MBEL55E	NC_006300
Marinobacter aquaeolei VT8	NC_008740
Marinomonas sp. MWYL1	NC_009654
Melissococcus plutonius ATCC 35311	NC_015516
Methylococcus capsulatus str. Bath	NC_002977
Methylomonas methanica MC09	NC_015572
Moritella yayanosii str. DB21MT	LS483250
Myxococcus macrosporus str. HW-1	NC_015711
Nitrococcus mobilis Nb-231	CH672427
Nitrosococcus oceani ATCC 19707	CP000127
Oceanimonas sp. GK1	NC_016745
Oenococcus oeni PSU-1	NC_008528
Paenibacillus larvae DSM 25430	NC_023134
Paenibacillus polymyxa SC2	NC_014622
Paenibacillus terrae HPL-003	NC_016641
Pantoea vagans C9-1	NC_014562
Parageobacillus thermoglucosidasius C56-YS93	NC_015660
Pasteurella multocida str. Pm70	NC_002663
Pectobacterium atrosepticum SCRI1043	NC_004547
Pediococcus claussenii ATCC BAA-344	NC_016605
Pediococcus pentosaceus SL4	NC_022780
Photobacterium profundum SS9	NC_006370
Photorhabdus luminescens TTO1	BX470251
Planococcus antarcticus DSM 14505	CP016534
Planococcus faecalis str. AJ003	CP019401
Planococcus sp. PAMC 21323	CP009129
Planomicrobium glaciei str. 46093	CP041323
Proteus mirabilis str. HI4320	NC_010554
Pseudoalteromonas atlantica T6c	NC_008228
Pseudoalteromonas haloplanktis str. TAC125	NC_007481
Pseudoalteromonas tunicata str. D2	CP031961
Pseudomonas aeruginosa PAO1	NC_002516
Pseudomonas entomophila str. L48	NC_008027
Pseudomonas fluorescens Pf0-*1*	NC_007492
Pseudomonas mendocina ymp	NC_009439
Pseudomonas putida KT2440	NC_002947
Pseudomonas stutzeri A1501	NC_009434
Pseudomonas syringae str. DC3000	NC_004578
Psychrobacter cryohalolentis K5	NC_007969
Psychrobacter sp. PRwf-1	NC_009524
Psychromonas ingrahamii 37	NC_008709
Psychromonas sp. CNPT3	NC_020802
Ralstonia solanacearum GMI1000	NC_003295
Rhodospirillum rubrum ATCC 11170	NC_007643

Rickettsiella viridis str. Ap-RA04	AP018005
Ruthia magnifica str. Cm	NC_008610
Saccharophagus degradans 2-40	NC_007912
Salinisphaera sp. LB1	CP029488
Salmonella enterica Typhi str. CT18	NC_003198
Salmonella enterica Typhimurium LT2	NC_003197
Serratia proteamaculans 568	NC_009832
Shewanella amazonensis SB2B	NC_008700
Shewanella baltica OS678	NC_016901
Shewanella denitrificans OS217	NC_007954
Shewanella frigidimarina NCIMB 400	NC_008345
Shewanella loihica PV-4	NC_009092
Shewanella oneidensis MR-1	AE014299
Shewanella pealeana ATCC 700345	NC_009901
Shewanella putrefaciens CN-32	NC_009438
Shewanella woodyi ATCC 51908	NC_010506
Sinorhizobium meliloti 1021	NC_003047
Sodalis glossinidius str. 'morsitans'	NC_007712
Solibacillus silvestris StLB046	NC_018065
Solimonas sp. K1W22B-7	CP031704
Sporolactobacillus terrae str. DRG1	CP025689
Staphylococcus argenteus str. B3-25B	CP042286
Staphylococcus aureus M1	NC_021059
Staphylococcus carnosus TM300	NC_012121
Staphylococcus epidermidis RP62A	NC_002976
Staphylococcus haemolyticus JCSC1435	NC_007168
Staphylococcus lugdunensis N920143	NC_017353
Staphylococcus pseudintermedius ED99	NC_017568
Staphylococcus warneri SG1	NC_020164
Stenotrophomonas maltophilia R551-3	NC_011071
Steroidobacter denitrificans str. DSM 18526	CP011971
Streptococcus intermedius C270	NC_022237
Streptococcus mutans LJ23	NC_017768
Streptococcus parasanguinis FW213	NC_017905
Streptococcus pneumoniae D39	NC_008533
Streptococcus pyogenes M1	NC_020540
Streptococcus salivarius JIM8777	NC_017595
Streptococcus suis A7	NC_017622
Streptococcus thermophilus MN-ZLW-002	NC_017927
Thermoactinomyces vulgaris str. CDF	CP036487
Thiomicrospira crunogena XCL-2	NC_007520
Tolumonas auensis DSM 9187	NC_012691
Tumebacillus avium str. AR23208	CP021434
Turicibacter sp. H121	CP013476
Veillonella parvula DSM 2008	NC_013520
Vesicomyosocius okutanii HA	NC_009465
Vibrio alginolyticus NBRC 15630	NC_022349
Vibrio cholerae O1 str. N16961	NC_002505
Vibrio fischeri ES114	NC_006840
Vibrio parahaemolyticus BB22OP	NC_019955

Vibrio vulnificus YJ016	NC_005139
Weissella koreensis KACC 15510	NC_015759
Xanthomonas axonopodis Xac29-1	NC_020800
Xanthomonas campestris str. ATCC 33913	NC_003902
Xenorhabdus bovienii SS-2004	NC_013892
Xylella fastidiosa 9a5c	NC_002488
Yersinia pestis CO92	NC_003143

Table S2. Proteins part of the phylogenetic analysis of the *Proteobacteria* and *Acidobacteria*.

Gene	Product	Mean length (aa)
FusA	Translation elongation factor G	702
NusG	Transcription termination factor NusG	183
RplA	50S ribosomal subunit protein L1	233
RplB	50S ribosomal subunit protein L2	275
RplC	50S ribosomal subunit protein L3	214
RplD	50S ribosomal subunit protein L4	203
RplE	50S ribosomal subunit protein L5	181
RplF	50S ribosomal subunit protein L6	178
RplJ	50S ribosomal subunit protein L10	169
RplK	50S ribosomal subunit protein L11	143
RplL	50S ribosomal subunit protein L12	124
RplN	50S ribosomal subunit protein L14	123
RplO	50S ribosomal subunit protein L15	146
RplP	50S ribosomal subunit protein L16	138
RplQ	50S ribosomal subunit protein L17	131
RplR	50S ribosomal subunit protein L18	118
RplV	50S ribosomal subunit protein L22	113
RplW	50S ribosomal subunit protein L23	101
RplX	50S ribosomal subunit protein L24	105
RpmC	50S ribosomal subunit protein L29	65
RpmD	50S ribosomal subunit protein L30	64
RpoA	RNA polymerase subunit alpha	332
RpoB	RNA polymerase subunit beta	1357
RpoC	RNA polymerase subunit beta-prime	1410
RpsC	30S ribosomal subunit protein S3	233
RpsD	30S ribosomal subunit protein S4	208
RpsE	30S ribosomal subunit protein S5	169
RpsG	30S ribosomal subunit protein S7	158
RpsH	30S ribosomal subunit protein S8	131
RpsJ	30S ribosomal subunit protein S10	104
RpsK	30S ribosomal subunit protein S11	131
RpsL	30S ribosomal subunit protein S12	125
RpsM	30S ribosomal subunit protein S13	120
RpsN	30S ribosomal subunit protein S14	99
RpsQ	30S ribosomal subunit protein S15	87
RpsS	30S ribosomal subunit protein S19	92
SecE	Sec translocon subunit SecE	125
SecY	Sec translocon subunit SecY	443
TufA	Translation elongation factor Tu	396

Table S3. Proteins part of the phylogenetic analysis of the *Firmicutes* and *Tenericutes*.

Gene	Product	Mean length (aa)
Adk	Adenylate kinase	215
CysS	Cysteine-tRNA ligase	466
FusA	Translation elongation factor G	695
InfA	Translation initiation factor IF-1	73
NusG	Transcription termination factor NusG	182
RlmB	23S rRNA 2'-O-ribose G2251 methyltransferase	255
RplA	50S ribosomal subunit protein L1	232
RplB	50S ribosomal subunit protein L2	278
RplC	50S ribosomal subunit protein L3	213
RplD	50S ribosomal subunit protein L4	208
RplE	50S ribosomal subunit protein L5	181
RplF	50S ribosomal subunit protein L6	179
RplJ	50S ribosomal subunit protein L10	170
RplK	50S ribosomal subunit protein L11	143
RplL	50S ribosomal subunit protein L12	122
RplM	50S ribosomal subunit protein L13	147
RplN	50S ribosomal subunit protein L14	122
RplO	50S ribosomal subunit protein L15	147
RplP	50S ribosomal subunit protein L16	144
RplQ	50S ribosomal subunit protein L17	126
RplR	50S ribosomal subunit protein L18	120
RplV	50S ribosomal subunit protein L22	116
RplW	50S ribosomal subunit protein L23	96
RplX	50S ribosomal subunit protein L24	105
RpmC	50S ribosomal subunit protein L29	67
RpmJ	50S ribosomal subunit protein L36	38
RpoA	RNA polymerase subunit alpha	315
RpoB	RNA polymerase subunit beta	1198
RpoC	RNA polymerase subunit beta-prime	1214
RpsC	30S ribosomal subunit protein S3	222
RpsE	30S ribosomal subunit protein S5	167
RpsG	30S ribosomal subunit protein S7	157
RpsH	30S ribosomal subunit protein S8	133
RpsI	30S ribosomal subunit protein S9	131
RpsJ	30S ribosomal subunit protein S10	103
RpsK	30S ribosomal subunit protein S11	131
RpsL	30S ribosomal subunit protein S12	138
RpsM	30S ribosomal subunit protein S13	122
RpsQ	30S ribosomal subunit protein S15	88
RpsS	30S ribosomal subunit protein S19	93
SecE	Sec translocon subunit SecE	63
SecY	Sec translocon subunit SecY	432
TruA	tRNA pseudouridine(38-40) synthase	255
TufA	Translation elongation factor Tu	397

Table S6. Overview over evolutionary events.

Class	Event	Operon	Description
Gammaproteobacteria	P1	*secE*	Δ*tRNA-Trp*
Gammaproteobacteria	P2	*tufB*/*str*	Inversion: *tufA*/*tufB*
Gammaproteobacteria	P3	*tufB*/*secE*	Disconnection: *tufB*/*secE*
Gammaproteobacteria	P4	*tufB*/*secE*	Disconnection: *tufB*/*secE*
Gammaproteobacteria	P5	*tufB*/*secE*	Δ*tufB*, Δ*tRNA-Trp*
Gammaproteobacteria	P6	*secE*	Δ*tRNA-Trp*
Gammaproteobacteria	P7	*tufB*	Δ*tufB*
Gammaproteobacteria	P8	*tufB*/*secE*	Insertion: *tufB*/*tRNA-Trp*
Gammaproteobacteria	P9	*tufB*/*secE*	Disconnection: *tufB*/*tRNA-Trp*
Gammaproteobacteria	P10	*tufB*	Δ*tufB*
Gammaproteobacteria	P11	*tufB*	Δ*tufB*
Gammaproteobacteria	P12	*tufB*	Δ*tufB*
Gammaproteobacteria	P13	*tufB*	Δ*tufB*
Gammaproteobacteria	P14	*tufB*/*str*	Inversion: *tufA*/*tufB*
Gammaproteobacteria	P15	*tufB*	Δ*tufB*
Gammaproteobacteria	P16	*secE*/*rpoBC*	Disconnection: *nusG*/*rplK*
Gammaproteobacteria	P17	*secE*/*rpoBC*	Disconnection: *nusG*/*rplK*
Gammaproteobacteria	P18	*secE*/*rpoBC*	Insertion: *nusG*/*rplK*
Gammaproteobacteria	P19	*secE*/*rpoBC*	Disconnection: *nusG*/*rplK*
Gammaproteobacteria	P20	*secE*/*rpoBC*	Insertion: *nusG*/*rplK*
Gammaproteobacteria	P21	*rpoBC*	Insertion: *rplA*/*rplJ*
Gammaproteobacteria	P22	*rpoBC*	Disconnection: *rplA*/*rplJ*
Gammaproteobacteria	P23	*rpoBC*	Translocation: *rplJ*, *rplL*
Gammaproteobacteria	P24	*rpoBC*	Disconnection: *rplA*/*rplJ*
Gammaproteobacteria	P25	*rpoBC*	Disconnection: *rplL*/*rpoB*
Gammaproteobacteria	P26	*rpoBC*	Insertion: *rplL*/*rpoB*
Gammaproteobacteria	P27	*rpoBC*	Disconnection: *rplL*/*rpoB*
Gammaproteobacteria	P28	*rpoBC*	Insertion: *rplL*/*rpoB*
Gammaproteobacteria	P29	*rpoBC*/*str*	Insertion: *rpoC*/*rpsL*
Gammaproteobacteria	P30	*rpoBC*/*str*	Insertion: *rpoC*/*rpsL*
Gammaproteobacteria	P31	*rpoBC*/*str*	Disconnection: *rpoC*/*rpsL*
Gammaproteobacteria	P32	*rpoBC*/*str*	Disconnection: *rpoC*/*rpsL*
Gammaproteobacteria	P33	*rpoBC*/*str*	Insertion: *rpoC*/*rpsL*
Gammaproteobacteria	P34	*rpoBC*/*str*	Disconnection: *rpoC*/*rpsL*
Gammaproteobacteria	P35	*rpoBC*/*str*	Disconnection: *rpoC*/*rpsL*
Gammaproteobacteria	P36	*rpoBC*/*str*	Disconnection: *rpoC*/*rpsL*
Gammaproteobacteria	P37	*str*	Δ*tufA*
Gammaproteobacteria	P38	*str*/*S10*	Insertion: *tufA*/*rpsJ*
Gammaproteobacteria	P39	*str*/*S10*	Disconnection: *tufA*/*rpsJ*
Gammaproteobacteria	P40	*str*/*S10*	Insertion: *tufA*/*rpsJ*
Gammaproteobacteria	P41	*str*/*S10*	Disconnection: *tufA*/*rpsJ*
Gammaproteobacteria	P42	*str*/*S10*	Disconnection: *tufA*/*rpsJ*
Gammaproteobacteria	P43	*S10*/*spc*	Insertion: *rpsQ*/*rplN*
Gammaproteobacteria	P44	*S10*/*spc*	Insertion: *rpsQ*/*rplN*
Gammaproteobacteria	P45	*S10*/*spc*	Insertion: *rpsQ*/*rplN*
Gammaproteobacteria	P46	*S10*/*spc*	Transposon insertion: *rpsQ*/*rplN*
Gammaproteobacteria	P47	*S10*/*spc*	Disconnection: *rpsQ*/*rplN*
Gammaproteobacteria	P48	*S10*/*spc*	Insertion: *rpsQ*/*rplN*

Gammaproteobacteria	P49	*S10*/*spc*	Insertion: *rpsQ*/*rplN*
Gammaproteobacteria	P50	*spc*	Δ*rpmJ*
Gammaproteobacteria	P51	*spc*	Δ*rpmJ*
Gammaproteobacteria	P52	*spc*	Δ*rpmJ*
Bacilli	F1	*cysS*	Disconnection: *cysS*/*rnc*
Bacilli	F2	*cysS*	Δ*rnc*
Bacilli	F3	*cysS*	Insertion: *rnc*/*rlmB*
Bacilli	F4	*cysS*	Δ*orf1*
Bacilli	F5	*cysS*	Δ*orf1*
Bacilli	F6	*cysS*	Δ*orf1*
Bacilli	F7	*cysS*	Δ*orf1*
Bacilli	F8	*cysS*/*secE*	Insertion: *sigH*/*rpmG*
Bacilli	F9	*cysS*	Disconnection: *rlmB*/*rpmG*
Bacilli	F10	*cysS*	Δ*orf1*, Δ*sigH*
Bacilli	F11	*cysS*	Δ*orf1*, Δ*sigH*
Bacilli	F12	*cysS*/*secE*	Insertion: *sigH*/*rpmG*
Bacilli	F13	*secE*	Δ*rpmG*
Bacilli	F14	*cysS*/*secE*	Insertion: *sigH*/*rpmG*
Bacilli	F15	*secE*	Δ*rpmG*
Bacilli	F16	*secE*	Δ*rpmG*
Bacilli	F17	*secE*	Δ*rpmG*
Bacilli	F18	*secE*	Δ*rpmG*
Bacilli	F19	*cysS*/*secE*	Insertion: *sigH*/*rpmG*
Bacilli	F20	*secE*	Δ*rpmG*
Bacilli	F21	*cysS*/*secE*	Insertion: *sigH*/*rpmG*
Bacilli	F22	*secE*	Disconnection: *secE*/*nusG*
Bacilli	F23	*secE*	Transposon insertion: *secE*/*nusG*
Bacilli	F24	*secE*	Insertion: *secE*/*nusG*
Bacilli	F25	*secE*/*rpoBC*	Insertion: *nusG*/rplK
Bacilli	F26	*secE*/*rpoBC*	Insertion: *nusG*/rplK
Bacilli	F27	*secE*/*rpoBC*	Insertion: *nusG*/rplK
Bacilli	F28	*secE*/*rpoBC*	Insertion: *nusG*/rplK
Bacilli	F29	*secE*/*rpoBC*	Insertion: *nusG*/rplK
Bacilli	F30	*secE*/*rpoBC*	Insertion: *nusG*/rplK
Bacilli	F31	*rpoBC*	Insertion: *rplA*/*rplJ*
Bacilli	F32	*rpoBC*	Disconnection: *rplA*/*rplJ*
Bacilli	F33	*rpoBC*	Transposon insertion: *rplA*/*rplJ*
Bacilli	F34	*rpoBC*	Insertion: *rplL*/*rsmC*
Bacilli	F35	*rpoBC*	Insertion: *rplL*/*rsmC*
Bacilli	F36	*rpoBC*	Disconnection: *rplL*/*rsmC*
Bacilli	F37	*rpoBC*	Insertion: *rplL*/*rsmC*
Bacilli	F38	*rpoBC*	Insertion: *rsmC*/*rpoB*
Bacilli	F39	*rpoBC*	Δ*rsmC*
Bacilli	F40	*rpoBC*	Insertion: *rplL*/*rsmC*
Bacilli	F41	*rpoBC*	Insertion: *rplL*/*rpoB*
Bacilli	F42	*rpoBC*	Δ*rsmC*
Bacilli	F43	*rpoBC*	Insertion: *rplL*/*rsmC*
Bacilli	F44	*rpoBC*	Insertion: *rplL*/*rpoB*
Bacilli	F45	*rpoBC*	Δ*rsmC*
Bacilli	F46	*rpoBC*	Insertion: *rplL*/*rsmC*

Bacilli	F47	*rpoBC*	Transposon insertion: *rplL*/*rsmC*
Bacilli	F48	*rpoBC*	Insertion: *rplL*/rsmC
Bacilli	F49	*rpoBC*	Insertion: *rsmC*/*rpoB*
Bacilli	F50	*rpoBC*	Insertion: *rsmC*/*rpoB*
Bacilli	F51	*rpoBC*/*str*	Insertion: *rpoC*/*rpsL*
Bacilli	F52	*str*	Δ*rpl7ae*
Bacilli	F53	*rpoBC*/*str*	Insertion: *rpoC*/*rpl7ae*
Bacilli	F54	*rpoBC*/*str*	Insertion: *rpoC*/*rpl7ae*
Bacilli	F55	*str*	Δ*rpl7ae*
Bacilli	F56	*rpoBC/str*	Disconnection: *rpoC*/*rpsL*
Bacilli	F57	*str*	Δ*rpl7ae*
Bacilli	F58	*str*	Δ*rpl7ae*
Bacilli	F59	*str*	Insertion: *rpsG*/*fusA*
Bacilli	F60	*str*/*S10*	Insertion: *fusA*/*rpsJ*
Bacilli	F61	*str*	Translocation: *tufA*
Bacilli	F62	*str*/*S10*	Insertion: *fusA*/*rpsJ*
Bacilli	F63	*str*/*S10*	Disconnection: *fusA*/*rpsJ*
Bacilli	F64	*str*	Translocation: *tufA*
Bacilli	F65	*str*/*S10*	Insertion: *tufA*/*rpsJ*
Bacilli	F66	*str*/*S10*	Insertion: *tufA*/*rpsJ*
Bacilli	F67	*str*/*S10*	Insertion: *tufA*/*rpsJ*
Bacilli	F68	*str*/*S10*	Disconnection: *tufA*/*rpsJ*
Bacilli	F69	*str*/*S10*	Insertion: *tufA*/*rpsJ*
Bacilli	F70	*str*/*S10*	Disconnection: *tufA*/*rpsJ*
Bacilli	F71	*str*/*S10*	Disconnection: *tufA*/*rpsJ*
Bacilli	F72	*str*/*S10*	Insertion: *tufA*/*rpsJ*
Bacilli	F73	*str*/*S10*	Insertion: *tufA*/*rpsJ*
Bacilli	F74	*str*/*S10*	Disconnection: *tufA*/*rpsJ*
Bacilli	F75	*str*/*S10*	Insertion: *tufA*/*rpsJ*
Bacilli	F76	*str*/*S10*	Transposon insertion: *tufA*/*rpsJ*
Bacilli	F77	*str*/*S10*	Insertion: *tufA*/*rpsJ*
Bacilli	F78	*str*/*S10*	Insertion: *tufA*/*rpsJ*
Bacilli	F79	*str*/*S10*	Insertion: *tufA*/*rpsJ*
Bacilli	F80	*str*/*S10*	Disconnection: *tufA*/*rpsJ*
Bacilli	F81	*spc*	Translocation: *rpsN*
Bacilli	F82	*spc*	Insertion: *rpsN*/*rpsH*
Bacilli	F83	*spc*	Insertion: *rpmD*/*rplO*
Bacilli	F84	*spc*	Δ*secY*
Bacilli	F85	*spc*	Δ*map*
Bacilli	F86	*spc*	Insertion: *secY*/*infA*
Bacilli	F87	*spc*	Δ*map*
Bacilli	F88	*spc*	Δ*map*
Bacilli	F89	*spc*	Δ*map*
Bacilli	F90	*spc*	Δ*map*
Bacilli	F91	*spc*	Δ*map*
Bacilli	F92	*alpha*	Δ*rpsD*
Bacilli	F93	*alpha*	Δ*rpsD*
Bacilli	F94	*alpha*	Δ*rpsD*
Bacilli	F95	*alpha*/*ecf*	Insertion: *rplQ*/*ecfA1*
Bacilli	F96	*alpha*/*ecf*	Insertion: *rplQ*/*ecfA1*

Bacilli	F97	*alpha*/*ecf*	Disconnection: *rplQ*/*ecfA1*
Bacilli	F98	*alpha*/*ecf*	Insertion: *rplQ*/*ecfA1*
Bacilli	F99	*alpha*/*ecf*	Disconnection: *rplQ*/*ecfA1*
Bacilli	F100	*alpha*/*ecf*	Insertion: *rplQ*/*ecfA1*
Bacilli	F101	*alpha*/*ecf*	Insertion: *rplQ*/*ecfA1*
Bacilli	F102	*alpha*/*ecf*	Insertion: *rplQ*/*ecfA1*
Bacilli	F103	*alpha*/*ecf*	Transposon insertion: *rplQ*/*ecfA1*
Bacilli	F104	*alpha*/*ecf*	Insertion: *rplQ*/*ecfA1*
Bacilli	F105	*ecf*	Δ*ecfA1A2T*
Bacilli	F106	*ecf*	Disconnection: *ecfT*/*truA*
Bacilli	F107	*ecf*	Insertion: *truA*/*rplM*
Bacilli	F108	*ecf*	Insertion: *truA*/*rplM*
Bacilli	F109	*ecf*	Disconnection: *truA*/*rplM*
Bacilli	F110	*ecf*	Insertion: *truA*/*rplM*
Bacilli	F111	*ecf*	Disconnection: *truA*/*rplM*

Table S7. List of *Proteobacteria* and *Acidobacteria* that carry two copies of the *rpmJ* gene within their chromosomes.

Species	Order
Actinobacillus equuli str. 19392	*Pasteurellales*
Actinobacillus indolicus str. AIFJ1607	*Pasteurellales*
Actinobacillus pleuropneumoniae L20	*Pasteurellales*
Actinobacillus porcitonsillarum str. 9953L55	*Pasteurellales*
Actinobacillus suis H91-0380	*Pasteurellales*
Aeromonas hydrophila ATCC 7966	*Aeromonadales*
Aeromonas veronii B565	*Aeromonadales*
Budvicia aquatica str. FDAARGOS_387	*Enterobacterales*
Cardiobacterium hominis str. NCTC10426	*Cardiobacteriales*
Citrobacter koseri ATCC BAA-89	*Enterobacterales*
Dickeya zeae Ech586	*Enterobacterales*
Edwardsiella tarda EIB202	*Enterobacterales*
Enterobacter sp. 638	*Enterobacterales*
Erwinia pyrifoliae DSM 12163	*Enterobacterales*
Haemophilus ducreyi 35000HP	*Pasteurellales*
Haemophilus parainfluenzae T3T1	*Pasteurellales*
Hafnia paralvei str. FDAARGOS_158	*Enterobacterales*
Klebsiella pneumoniae str. NCTC 418	*Enterobacterales*
Leclercia sp. LSNIH3	*Enterobacterales*
Pantoea vagans C9-1	*Enterobacterales*
Pasteurella multocida str. Pm70	*Pasteurellales*
Pectobacterium atrosepticum SCRI1043	*Enterobacterales*
Photobacterium profundum SS9	*Vibrionales*
Photorhabdus luminescens TTO1	*Enterobacterales*
Proteus mirabilis str. HI4320	*Enterobacterales*
Pseudomonas aeruginosa PAO1	*Pseudomonadales*
Psychrobacter sp. PRwf-1	*Pseudomonadales*
Psychromonas ingrahamii 37	*Alteromonadales*
Psychromonas sp. CNPT3	*Alteromonadales*
Salmonella enterica Typhi str. CT18	*Enterobacterales*
Salmonella enterica Typhimurium LT2	*Enterobacterales*
Serratia proteamaculans 568	Enterobacterales
Sodalis glossinidius str. 'morsitans'	Enterobacterales
Tolumonas auensis DSM 9187	Aeromonadales
Vibrio alginolyticus NBRC 15630	Vibrionales
Vibrio cholerae O1 str. N16961	Vibrionales
Vibrio parahaemolyticus BB22OP	Vibrionales
Vibrio vulnificus YJ016	Vibrionales
Xenorhabdus bovienii SS-2004	Enterobacterales
Yersinia pestis CO92	Enterobacterales

Table S8. List of *Firmicutes* and *Tenericutes* that carry two copies of the *rpsN* gene within their chromosomes.

Species	Order
[Bacillus] selenitireducens MLS10	*Bacillales*
Bacillus amyloliquefaciens CC178	*Bacillales*
Bacillus coagulans 36D1	*Bacillales*
Bacillus megaterium WSH-002	*Bacillales*
Bacillus paralicheniformis ATCC 9945a	*Bacillales*
Bacillus pseudofirmus OF4	*Bacillales*
Bacillus subtilis PY79	*Bacillales*
Carnobacterium maltaromaticum LMA28	*Lactobacillales*
Enterococcus faecalis D32	*Lactobacillales*
Enterococcus faecium Aus0085	*Lactobacillales*
Enterococcus hirae ATCC 9790	*Lactobacillales*
Enterococcus mundtii QU 25	*Lactobacillales*
Halobacillus halophilus DSM 2266	*Bacillales*
Lactobacillus acidophilus La-14	*Lactobacillales*
Lactobacillus brevis ATCC 367	*Lactobacillales*
Lactobacillus buchneri NRRL B-30929	*Lactobacillales*
Lactobacillus crispatus ST1	*Lactobacillales*
Lactobacillus fermentum CECT 5716	*Lactobacillales*
Lactobacillus plantarum JDM1	*Lactobacillales*
Listeria ivanovii PAM 55	*Bacillales*
Listeria monocytogenes M7	*Bacillales*
Listeria seeligeri serovar 1/2b str. SLCC3954	*Bacillales*
Listeria welshimeri serovar 6b str. SLCC5334	*Bacillales*
Macrococcus canis str. KM0218	*Bacillales*
Macrococcus caseolyticus JCSC5402	*Bacillales*
Mahella australiensis 50-1 BON	*Thermoanaerobacterales*
Paenibacillus polymyxa SC2	*Bacillales*
Paenibacillus terrae HPL-003	*Bacillales*
Pediococcus claussenii ATCC BAA-344	*Lactobacillales*
Pediococcus pentosaceus SL4	*Lactobacillales*
Planococcus antarcticus DSM 14505	*Bacillales*
Planococcus faecalis str. AJ003	*Bacillales*
Planococcus sp. PAMC 21323	*Bacillales*
Planomicrobium glaciei str. 46093	*Bacillales*
Sporolactobacillus terrae str. DRG1	*Bacillales*
Staphylococcus argenteus str. B3-25B	*Bacillales*
Staphylococcus aureus M1	*Bacillales*
Staphylococcus carnosus TM300	Bacillales
Staphylococcus epidermidis RP62A	Bacillales
Staphylococcus haemolyticus JCSC1435	Bacillales
Staphylococcus lugdunensis N920143	Bacillales
Staphylococcus pseudintermedius ED99	Bacillales
Staphylococcus warneri SG1	Bacillales
Streptococcus mutans LJ23	Lactobacillales
Streptococcus pyogenes M1	Lactobacillales
Streptococcus suis A7	Lactobacillales

Table S9. Number of operon cluster alteration events per branch.

Number of alteration events	Number of branches
0	228
1	64
2	16
3	12
4	2
5	0
6	0
7	2
8	1

Table S10. Operon alteration events per amino acid substitution within the orders of the *Gammaproteobacteria*.

Taxonomic order	Alteration events per 1,000 aa substitutions
Enterobacterales	0.28
Orbales	0.61
Pasteurellales	3.24
Vibrionales	0
Aeromonadales	0
Alteromonadales	0.70
Cellvibrionales	0.71
Pseudomonadales	0.29
Oceanospirillales	0.13
Thiotrichales	0.30
Legionellales	0.16
unclassified sulfur-oxidizing symbionts	0.19
Methylococcales	0.16
Chromatiales	0
Salinisphaerales	0
Nevskiales	0
Xanthomondales	0.36
Cardiobacteriales	0.18
